# Supplementary material for: Population health impact, cost-effectiveness, and affordability of community-based HIV treatment and monitoring in South Africa: A health economics modelling study
Source: PLOS Glob Public Health. 2023 Sep 5;3(9):e0000610. doi: 10.1371/journal.pgph.0000610 (PMC10479912; doi:10.1371/journal.pgph.0000610)
Supplement: S1 Appendix — (PDF) [file pgph.0000610.s001.pdf]

Supplementary appendix

Mathematical Modeling Analysis

I. Model overview
II. Model modules and parameter values
  a. Demography
  b. Sexual behavior
  c. HIV Natural history
  d. Transmission probabilities
  e. Historical interventions
    i. HIV antiretroviral therapy
    ii. Condoms
    iii. Circumcision
  f. Future assumptions and interventions
    i. Demography
    ii. HIV antiretroviral therapy
    iii. Home HIV testing and counseling campaigns
    iv. Circumcision
III. Model calibration and validation
  a. Calibration
    i. Phase 0
    ii. Bayesian calibration approach
    iii. Phase 1
    iv. Phase 2
    v. Approximate Bayesian Computation- Sequential Monte Carlo (ABC-SMC) algorithm
    vi. Likelihood calculation
  b. Validation
IV. Model differential equations
  a. Demography
  b. Sexual behavior
  c. Transmission probabilities
  d. HIV natural history and interventions
V. Scenario descriptions
VI. Outcomes by gender and age
VII. Additional modeling scenarios
  a. Impact of home HIV testing and counseling campaigns
  b. Circumcision scale-up sensitivity analysis

Cost-Effectiveness Analysis

VIII. Costs
IX. Total costs for each HIV intervention scenario
X. DALY and QALY calculation
XI. Cost-effectiveness sensitivity analyses

References

Mathematical Modeling Analysis

(I.) Model overview

The *DRIVE (Data-driven Recommendations for Interventions against Viral infEction)* model is a deterministic, compartmental model parameterized to represent human immunodeficiency virus (HIV) and human papillomavirus (HPV) transmission and disease progression in KwaZulu-Natal, South Africa (KZN), a region with high HIV prevalence(1, 2). The model was adapted from a previously published model fit to this context(3). Since the present analysis focuses on HIV outcomes and we do not model an effect of HPV infection on HIV acquisition or disease progression, and the number cervical cancer deaths is small relative to the total population size, we exclude detailed description of HPV natural history from this appendix. However, we highlight aspects where HPV or cervical cancer dynamics influenced model development.

The primary objective of our HIV module is to predict the impact of prevention and treatment interventions on disease outcomes. The population is stratified by age, gender, and sexual risk group and engages in heterosexual transmission of HIV infection beginning in 1980. HIV progression is tracked by both CD4+ T-cell (CD4) count and HIV RNA concentration (viral load), and infected individuals may achieve viral suppression with initiation of antiretroviral therapy (ART), beginning in 2004. The model is calibrated to reproduce observed population-level HIV dynamics using maximum-likelihood estimation.

Model dynamics are governed by a system of differential equations that are solved in MATLAB using a 4th-order Runge-Kutta numerical method. Simulation begins in 1925 to allow HPV transmission dynamics and cervical cancer incidence to equilibrate prior to the introduction of HIV infection in 1980. The model calculates events in discrete time with two-month intervals. At each time step, differential equations are evaluated to estimate population demographics and the number of persons in each infection, disease, or treatment state for the following time step.

This work was facilitated through the use of advanced computational, storage, and networking infrastructure provided by the Hyak supercomputer system at the University of Washington.

(II.) Modules and parameter values

(II.a.) Demography

The model represents a dynamic, open population of men and women aged 0 to 79 in KwaZulu-Natal. To allow HPV transmission dynamics to equilibrate prior to the introduction of HIV in 1980, the model is initialized in 1925. Because data on population demographics are not available prior to 1950, we projected backwards from more recent estimates. The United Nations Population Division estimates the South African population by gender and age from 1950 to 2020, and Statistics South Africa calculates the proportion of the population residing in KZN in the years 2002 to 2019(4, 5). KZN's share of the total population decreased linearly during this period, and we assumed the trend was slightly steeper between 1925 and 2002 to provide an initial population size large enough to absorb HIV-associated deaths and match observed data for KZN in 2002. In this way, we derived the KZN population in 1950 by gender and age from national data, and then fit exponential distributions for each gender and age group to project backwards to 1925 (Table A).

At each two-month time step, we calculate the number of births, natural deaths, and persons aging to the next five-year group. Total fertility rates determine how many male and female newborns enter the population over time. We model a 56% linear decline in fertility from 1960 to 2000, followed by a ten-year stabilization until 2010, and then a second 24% linear decrease from 2010 values by 2020 consistent with literature, United Nations Population Division total fertility estimates, and KZN population estimates(4-7). These rates are applied to women aged 15-49, stratified by age and by CD4 cell count in women with HIV(8, 9) (Table B). The proportion of births from mothers with HIV that result in perinatal infection decreases linearly from 34% in 2004 to 29.2% by 2005, and to 7.1% by 2008, capturing improvements in services for pregnant women living with HIV(10-12) (Table C). We assume a 1:1 gender ratio at birth and that infected newborns are born into the acute stage of HIV.

To age the population, one-fifth of each compartment moves to the next age group annually. Upon aging to the next five-year group, individuals are re-distributed into the closest unfilled risk group to match observed data on the age distribution of low, moderate, and high-risk individuals (defined by annual number of sexual partnerships; see section II.b. on Sexual Behavior). All compartments, except for the youngest 0 to 4 age group thus experience inflows from the prior age group and outflows into the next age group.

Persons leave the population due to death or aging past age 79. To model deaths, we apply South African age- and sex-specific mortality rates (Table D). Before 1950, we use United Nations 2019 Population Prospects abridged life tables from the period of 1950 to 1955(4). We then assume that background mortality decreases linearly to 1985 Population Prospects values. After the start of the generalized HIV epidemic in 1980,

we account for both background and HIV-specific mortality. To estimate background mortality rates, we subtract HIV-specific mortality from all-cause mortality estimates from the IHME Global Burden of Disease Study (13), and we assume linear changes in background mortality between 1985 and 2000 and between 2000 and 2020.

Table A. Initial population size by age and gender. The population distribution by gender and 5-year age groups was backward projected from observed data on the total South Africa population and proportion residing in KZN assuming exponential growth between 1925 and 1950(4, 5).

Age group	Initial population size	
	Men	Women
**0 – 4**	232193	234484
**5 – 9**	159981	163174
**10 – 14**	130604	133156
**15 – 19**	116664	114322
**20 – 24**	105385	99000
**25 – 29**	95487	87233
**30 – 39**	88296	80039
**35 – 39**	81236	71998
**40 – 44**	73180	65881
**45 – 49**	62270	56376
**50 – 54**	49095	47787
**55 – 59**	38061	38751
**60 – 64**	27479	32112
**65 – 69**	18576	23810
**70 – 74**	12335	15545
**75 – 79**	6595	7570
**TOTAL**	1297437	1271238

Table B. Baseline fertility rates by age and HIV status. Fertility rates before 1960(8, 9). Linear decreases in 1960 and 2010 are applied to this matrix.

Age group	Annual fertility rates before 1960					
	HIV-uninfected or WLHIV on ART	Acute	CD4 >500	CD4 350-500	CD4 200-350	CD4 <200
**0 – 14**	0	0	0	0	0	0
**15 – 19**	0.1555	0.1555	0.1555	0.0902	0.0902	0.0638
**20 – 24**	0.3058	0.3058	0.3058	0.1774	0.1774	0.1254
**25 – 29**	0.3120	0.3120	0.3120	0.1809	0.1809	0.1279
**30 – 34**	0.2323	0.2323	0.2323	0.1347	0.1347	0.0953
**35 – 39**	0.1483	0.1483	0.1483	0.0860	0.0860	0.0608
**40 – 44**	0.0596	0.0596	0.0596	0.0346	0.0346	0.0244
**45 – 49**	0.0194	0.0194	0.0194	0.0112	0.0112	0.0079
**50 – 79**	0	0	0	0	0	0

Table C. Proportion of births from women living with HIV that result in mother-to-child transmission. The mother to child transmission (MTCT) rate decreases linearly from 2004 to 2005 and from 2005 to 2008(10-12).

Year	Proportion of births with MTCT
**Before 2004**	0.340
**By 2005**	0.292
**After 2008**	0.071

Table D. Background mortality rates by age and gender. The population follows the background mortality rate of South Africa. Before 1950, we use United Nations data from the period of 1950 to 1955(4). We then assume that background mortality decreases linearly until 1985, the start of the generalized HIV epidemic. Background mortality changes linearly between 1985 and 2000 and between 2000 and 2020 according to estimates from the IHME Global Burden of Disease Study(13).

	**Annual background mortality rates**							
**Age group**	**1925 to 1950**		**By 1985**		**By 2000**		**2020 onward**	
	**Men**	**Women**	**Men**	**Women**	**Men**	**Women**	**Men**	**Women**
**0 – 4**	0.1901	0.1628	0.0570	0.0474	0.0144	0.0118	0.0054	0.0046
**5 – 9**	0.0087	0.0074	0.0025	0.0019	0.0013	0.0010	0.0004	0.0003
**10 – 14**	0.0048	0.0044	0.0016	0.0012	0.0010	0.0008	0.0002	0.0001
**15 – 19**	0.0060	0.0049	0.0025	0.0017	0.0022	0.0018	0.0007	0.0005
**20 – 24**	0.0085	0.0056	0.0038	0.0024	0.0044	0.0050	0.0021	0.0010
**25 – 29**	0.0094	0.0065	0.00438	0.0031	0.0079	0.0087	0.0030	0.0018
**30 – 34**	0.0105	0.0073	0.0052	0.0038	0.0110	0.0093	0.0040	0.0026
**35 – 39**	0.0122	0.0083	0.0064	0.0046	0.0131	0.0095	0.0049	0.0030
**40 – 44**	0.0144	0.0096	0.0080	0.0057	0.0141	0.0086	0.0055	0.0033
**45 – 49**	0.0166	0.0105	0.0101	0.0067	0.0172	0.0094	0.0074	0.0045
**50 – 54**	0.0206	0.0136	0.0140	0.0090	0.0227	0.0120	0.0108	0.0059
**55 – 59**	0.0255	0.0180	0.0186	0.0120	0.0282	0.0153	0.0161	0.0086
**60 - 64**	0.0352	0.0270	0.0278	0.0185	0.0378	0.0218	0.0230	0.0117
**65- 69**	0.0506	0.0420	0.0428	0.0304	0.0466	0.0303	0.0321	0.0165
**70 - 74**	0.0771	0.0674	0.0685	0.0517	0.0680	0.0452	0.0405	0.0231
**75 - 79**	0.1190	0.1070	0.1120	0.0881	0.0839	0.0559	0.0570	0.0353

(II.b.) Sexual behavior

In our model, sexual activity begins in the 10-14 age group. In each sexually active age group, the population is divided into three risk groups with variable rates of partnership formation (Table E). The distribution of these risk groups by age was derived from Africa Centre cohort partnership data(14), calculated as the percentage of the cohort in age groups 15-19 through 45-49 who reported 0-1 recent partners (low risk), 2-4 recent partners (moderate risk), or 5 or more recent partners (high risk). In age groups for which no respondents reported 5 or more recent partners, we borrowed information from neighboring age groups to estimate the proportion we would expect to be high-risk given that the sample size for some age groups was small. Individuals aged 10-14 are assumed to be predominately low-risk, and the risk distribution for ages 45-49 is extrapolated up to age 79. The risk distribution derived from male partner data is used for both men and women for simplicity. However, as described below, the rates of partner change and coital frequency within risk and age groups are calibrated and allowed to vary by gender.

Risk groups are distinguished by the number of sexual partners each person is expected to have per year. While data are available on the distribution of partnership counts from the Africa Centre cohort data(14), we calibrate the yearly partner change rates for each risk group and gender using prior ranges informed by these data (Table N). In addition to uncertainty in reported estimates due to reporting biases, this approach is intended to partially account for the effects of concurrency on increasing transmission. While this does not fully capture the effects of concurrency on transmission dynamics, our compartmental model structure is not equipped to represent concurrent partnerships(15); using partnership numbers as reported would underestimate the rate of infection spread. However, we define bounds on our priors to keep partnership counts within a plausible range. These parameters are defined such that higher risk groups have fewer acts per partnership per year, reflecting an assumption of shorter partnership duration in higher risk groups. We calibrate the distribution of acts per partnership by age in women (Table N). Among men, we assume those aged 10-19 have the same number of acts per partnership as women, whereas men aged 20-79 have equal acts to women of the next lowest age group, reflecting age disparities in relationships.

Using methods similar to other models (16), we define mixing matrices to describe patterns of sexual contact by age and sexual-risk groups. Informed by data on age discrepancies in KZN(17, 18), we define an age mixing matrix with partnerships being most likely to form between women and men of the next oldest 5-year age group (see section IV.b.). To account for uncertainty in this distribution, we calibrate a mixing parameter (ϵ_α; Table N), which determines the degree to which the distribution of partnerships by male and female age group deviates from this off-diagonal matrix. The parameter ϵ_α can range from 0, which corresponds to no change from this off-diagonal matrix, to 1 (random mixing), in which case mixing is proportional to the relative

sizes of all compartments. For mixing by risk group, we apply a parameter of $\epsilon_r = 0.3$ (Table F)(16). In this case, a value of $\epsilon_r = 0$ would correspond to an identity matrix with purely assortative (like-with-like) risk group mixing, whereas $\epsilon_r = 1$ corresponds to random mixing.

Because our model assumes purely heterosexual contact, the modeled number of partnerships expected for men must be consistent with the number expected for women. The observed data used to inform these parameters are subject to selection and response biases that may result in imbalances, and our calibration procedure may also result in imbalanced parameters. We thus adjust contact rates such that the number of partnerships among men in a given age and risk group equals the number of partnerships that women have with men of that same age and risk group. We assume that this adjusted contact rate is equally driven by estimated rates for men and women due to lack of data to assume otherwise.

Table E. Sexual risk distribution by age. Risk distribution is derived from partner data from the Africa Centre cohort (now on AHRI) for KwaZulu Natal, South Africa(14). The population aged 15-49 was divided in groups defined as low-risk (0-1 recent partners), moderate-risk (2-4 recent partners), or high risk (5+ recent partners). Children 10-14 are assumed to be predominantly low-risk, and the risk distribution for ages 45-49 is extrapolated up to age 79. The risk distribution from male partner data is used for both men and women in the model.

Age group	Risk distribution		
	Low-risk	Moderate-risk	High-risk
**10 – 14**	0.980	0.015	0.005
**15 – 19**	0.509	0.408	0.083
**20 – 24**	0.472	0.443	0.085
**25 – 29**	0.510	0.412	0.078
**30 – 34**	0.605	0.342	0.054
**35 – 39**	0.766	0.203	0.031
**40 – 44**	0.818	0.168	0.014
**45 – 49**	0.851	0.148	0.001
**50 – 54**	0.851	0.148	0.001
**55 – 59**	0.851	0.148	0.001
**60 - 64**	0.851	0.148	0.001
**65- 69**	0.851	0.148	0.001
**70 - 74**	0.851	0.148	0.001
**75 - 79**	0.851	0.148	0.001

Table F. Sexual mixing by sexual risk group (ϵ_r). (16)

Mixing by sexual risk group
0.3

(II.c.) HIV Natural history

HIV is introduced to the model in 1980 with an initial prevalence of 2% across all population subgroups. HIV infection occurs either through heterosexual transmission or mother to child. The natural history of HIV infection is then modeled in stages defined by CD4 count and viral load as shown in Figure A. When a person becomes HIV-infected, s/he enters the acute stage characterized by a short duration and high probability of onward HIV transmission. The person then progresses through stages of decreasing CD4 count. Viral load decreases from the acute to the asymptomatic phase and then increases. Transition rates are based on literature describing the average duration in each stage by gender and age (Tables G-H) (19-22). HIV-associated mortality rates with untreated infection are estimated from studies of untreated persons living with HIV and depend on CD4 cell count and age (Table I) (23-25). The combinations of disease progression and mortality rates imply that untreated women have a longer average expected survival than untreated men, and persons aged 5-49 live longer with untreated infection than young children and older adults. Children under age four have the highest HIV-specific mortality, and adults >50 years are assumed to experience HIV mortality rates two times that of persons aged 5-49(26, 27).

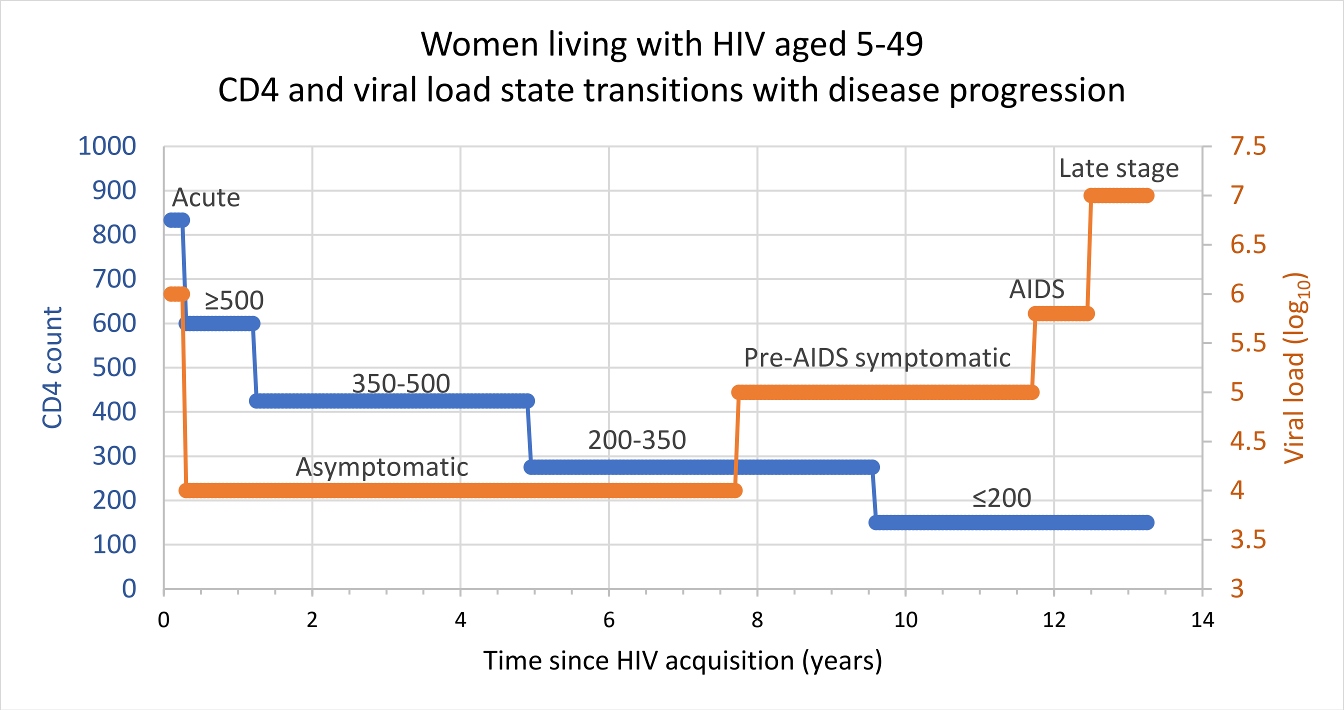

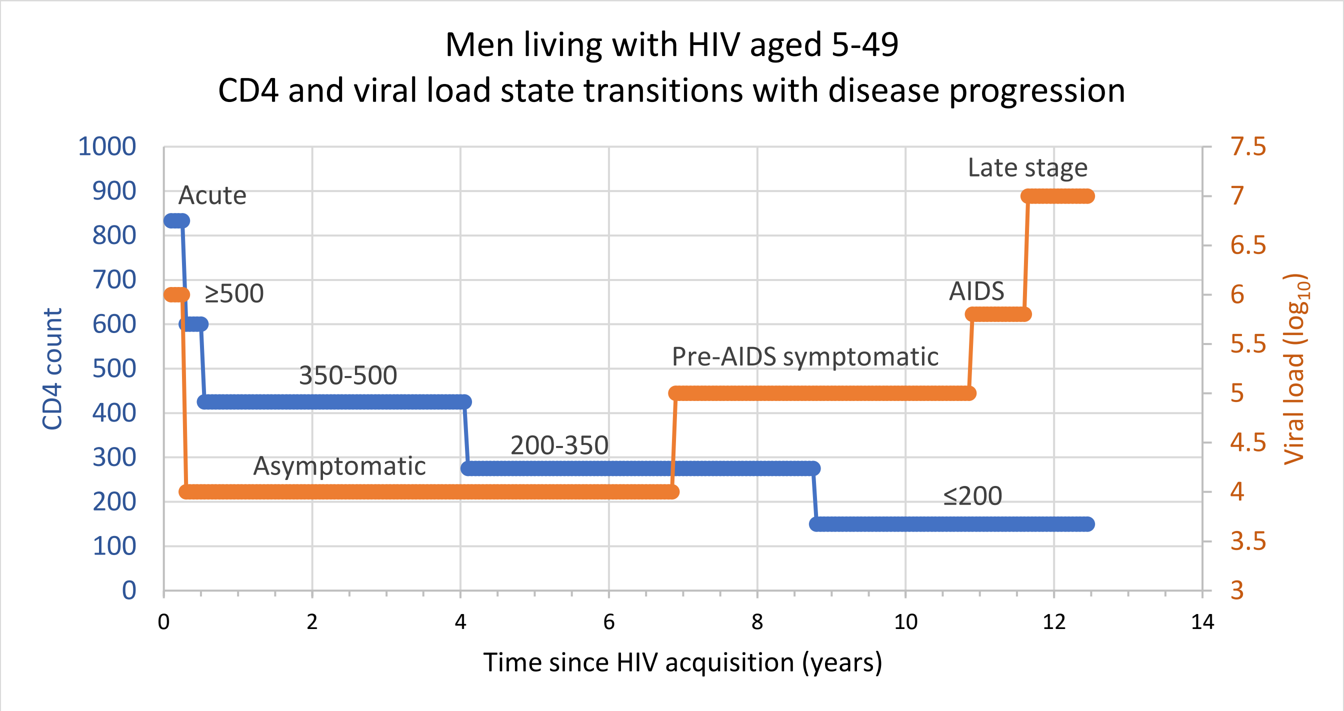

Figure A. HIV CD4 and viral load state transitions and durations for women (top) and men (bottom). Natural history of HIV infection. Values for CD4 count in the acute phase and for viral load in each phase of infection are approximated to capture the trajectory of disease over time. The implications of each CD4 and viral load state for mortality, HIV transmission, and susceptibility to HPV and cervical cancer are described in below sections of the appendix.

Table G. The duration of time in each CD4 stage by gender and age (20, 22). These average durations determine the rate at which individuals transition between states. The actual time spent in each state is slightly lower on average due to the effects of background and disease-specific mortality rates.

**Age group**	**Duration of time spent in CD4 stage with untreated infection (years)**				
	**Acute**	**CD4 ≥500**	**CD4 350-500**	**CD4 200-350**	**CD4 ≤200**
	**Men**				
**0 – 4**	0.25	0.25	3.56	4.67	2.13
**5 – 49**	0.25	0.25	3.56	4.67	3.70
**50 – 79**	0.25	0.25	2.85	4.51	1.85
	**Women**				
**0 – 4**	0.25	0.93	3.71	4.68	2.13
**5 – 49**	0.25	0.93	3.71	4.68	3.70
**50 – 79**	0.25	0.29	3.34	4.23	1.85

Table H. The duration of time in each viral load stage by gender and age (19, 21). We define time in the asymptomatic stage such that the total time spent in all viral load stages matches the total time spent in CD4 stages. This is based on data indicating that, while there is variability in time to AIDS, the trajectory in the pre-AIDS and AIDS phases are similar across groups(19).

**Age group**	**Duration of time spent in viral load stage (years)**				
	**Acute**	**Asymptomatic**	**Pre-AIDS symptomatic**	**AIDS**	**Late-stage**
	**Men**				
**0 – 4**	0.25	5.03	4.00	0.75	0.83
**5 – 49**	0.25	6.60	4.00	0.75	0.83
**50 – 79**	0.25	3.88	4.00	0.75	0.83
	**Women**				
**0 – 4**	0.25	5.87	4.00	0.75	0.83
**5 – 49**	0.25	7.44	4.00	0.75	0.83
**50 – 79**	0.25	4.13	4.00	0.75	0.83

Table I. HIV-associated mortality rates by age and CD4 count. HIV-associated mortality is estimated from observational studies of untreated persons with HIV and depends on CD4 cell count and age(23-25). Mortality from observed studies reflects both background and HIV-specific mortality, so we derived HIV-specific mortality by subtracting the average background mortality rate among men and women aged 30-34 (approximately the median age for the included studies) from the estimated rates. Children under age four have the highest disease mortality and adults >50 years have rates two times that of persons ages 5-49(26, 27).

**Age group**	**Annual HIV-associated mortality rates**				
	**Acute**	**CD4 ≥500**	**CD4 350-500**	**CD4 200-350**	**CD4 ≤200**
**0 – 4**	0	0.4700	0.4700	0.4700	0.4700
**5 – 49**	0	0.0035	0.0255	0.0455	0.2655
**50 – 79**	0	0.0071	0.0511	0.0911	0.5311

(II.d.) Transmission probabilities

The number of people who acquire HIV infection at each time step is determined by sex, age, and risk-group specific forces of infection. These rates are a function of the rate of partner change, the prevalence of infection, patterns of sexual mixing, and per-partnership transmission probabilities. Per-partnership transmission probabilities reflect the cumulative risk of acquiring HIV from all sexual acts assumed to occur within a partnership (per-partnership transmission = $1-(1-p)^a$, where a is the number of acts and p is the probability of transmission per act). The number of acts per partnership depends on the age and risk group of the partner without HIV, and is highest for young adults and those in lower risk groups, reflecting assumptions of higher coital frequency for young adults, and that partnerships are of longer duration.

For HIV transmission, the probability of transmission per sex act depends on the viral load of the partner with HIV. The risk of HIV transmission is highest during the initial acute stage of infection. Risk decreases during the asymptomatic phase of HIV infection before gradually rising as individuals progress to pre-AIDS symptomatic and AIDS stages (19, 28-30). We calibrate HIV transmission per act in the asymptomatic stage and apply risk multipliers from literature across the other stages of infection (Tables N, J). We assume the probability of male-to-female HIV transmission is equal to the probability of female-to-male transmission across all viral load stages.

As a proxy for decreased sexual activity due to pain and sickness during late-stage HIV, we reduce HIV per-act transmission to 10% of the AIDS rate (30). We assume women with regional or distant cervical cancer (FIGO stages 2-4) also decrease their sexual activity due to pain, reducing HIV transmission by 50%.

Table J. Risk multipliers for HIV transmission by viral load. The probability of HIV transmission per act depends on the viral load of the partner with HIV(19, 28-31).

Risk multiplier for HIV transmission				
**Acute**	**Asymptomatic**	**Pre-AIDS symptomatic**	**AIDS**	**Late-stage**
9.0	1.0	2.5	7.0	0.7

(II.e.) Historical interventions

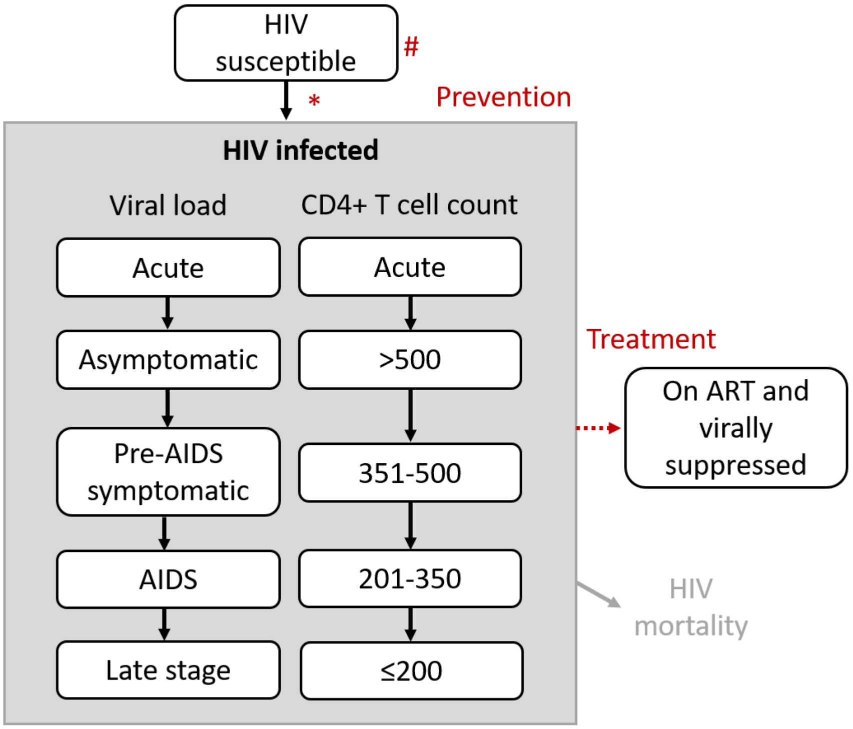

HIV susceptible men are stratified by circumcision status; circumcision decreases risk of HIV acquisition.
* Condoms decrease risk of HIV acquisition in both men and women.

Figure B. Effects of HIV-focused interventions on HIV natural history.

(II.e.i.) HIV antiretroviral therapy (ART)

Beginning in 2004, individuals may achieve viral suppression with initiation of ART. We only model the effect of ART among individuals who achieve viral suppression: persons with HIV who initiate treatment without achieving viral suppression are assumed to have no benefit from treatment and are not tracked in our model. Persons on treatment with viral suppression are assumed to have zero probability of transmitting HIV(32, 33), and to have the same fertility rates as women without HIV. HIV-associated mortality is reduced among treated persons, and it decreases over time as the average CD4 count at ART initiation increases(34-37), as described below.

We define ART coverage as the percentage of all persons living with HIV and age-eligible for ART who are on treatment and virally suppressed. We assume individuals aged 15-19 or older are eligible for ART because an age of 18 or older was required for DO ART Study enrollment. The scale-up of ART over time depends on CD4 count and gender. Reflecting changes in policy for treatment eligibility in South Africa(38),

ART becomes available in 2004 to persons with a CD4 count ≤200 cells/µL. The initiation threshold is subsequently raised to CD4 ≤350 cells/µL in 2011 and to CD4 ≤500 cells/µL in 2015. In 2016, ART becomes available to all individuals living with HIV regardless of their CD4 cell count, including those with acute infection. Informed by empirical data on ART coverage and viral suppression over time in South Africa and KwaZulu Natal specifically(2, 39-42), we model higher rates of viral suppression in women than men. The proportion of men living with HIV who are treated and achieve viral suppression increases from 0% in 2004 to approximately 18% in 2011, whereas the proportion of women who achieve viral suppression reaches 24% in 2011 (Table K)(2, 39-42). Between 2011 and 2017, we linearly increase the proportion of persons living with HIV who are treated and virally suppressed to values calculated as (proportion diagnosed)*(proportion on ART and virally suppressed) from South Africa national survey data(2) and DO ART Study data(43), respectively. This allows the modeled proportion of persons on ART and virally suppressed under the clinic-based standard of care to equilibrate to observed levels by 2020.

Among individuals of the same gender and CD4 cell count, the probability of ART initiation is uniform by age or risk group. However, we do not model the process of discontinuation of ART and resulting loss of viral suppression, such that the cumulative probability of being on ART increases with age. This results in proportions with viral suppression that are too low in younger ages and too high in older ages compared to observed data. To control this age differential, we apply a minimum bound of 0.85*(target population-level viral suppression) and a maximum bound of 1.04*(target population-level viral suppression) within each age group; eligible individuals are initiated on ART with viral suppression if coverage falls below the minimum for a given age group, and we discontinue treatment for some individuals if coverage exceeds the maximum within an age group. These age-specific minimum and maximum limits ensure that viral suppression is distributed more appropriately in all age groups while also matching the population-level levels of viral suppression by gender in the observed data.

Trends in HIV-associated mortality among treated persons living with HIV mirror changes to the ART initiation threshold to reflect higher baseline health among persons initiating treatment over time. HIV-associated mortality with treatment is defined relative to background mortality, informed by empirical data (34-37), and is applied additively. From 2004 to 2011, HIV-associated excess mortality among virally suppressed persons living with HIV is 0.5x the background rate (i.e., the mortality rate from background causes and HIV combined among persons with viral suppression is 1.5 times the background mortality rate). This multiplier decreases to 0.4x, 0.25x, and 0.15x the background mortality rate in 2011, 2015, and 2016, respectively (Table L).

Table K. Proportion of persons living with HIV on ART and virally suppressed (VS) in KZN. We derived estimates of viral suppression from observed data from 2005-2011 and 2017(2, 39-42). DO ART Study observed data inform 2017 values. Between these coverage targets, we assume a linear increase in viral suppression. Viral suppression is assumed to stay constant between 2017 and 2020.

Proportion virally suppressed over time by gender		
**Year**	**Men**	**Women**
**2004**	0	0
**2005**	0.0057	0.0077
**2006**	0.0218	0.0294
**2007**	0.0476	0.0643
**2008**	0.0820	0.1108
**2009**	0.1152	0.1557
**2010**	0.1416	0.1913
**2011**	0.1760	0.2378
**2017**	0.78 (diagnosed) * 0.51 (on ART, VS) = 0.3978	0.889 (diagnosed) * 0.70 (on ART, VS) = 0.6223

Table L. HIV-associated excess mortality with viral suppression(34-37).

HIV-associated mortality among individuals with HIV viral suppression	
**Time period**	**Multiplier on background mortality**
**2004 to 2011**	0.5
**2011 to 2015**	0.4
**2015 to 2016**	0.25
**After 2016**	0.15

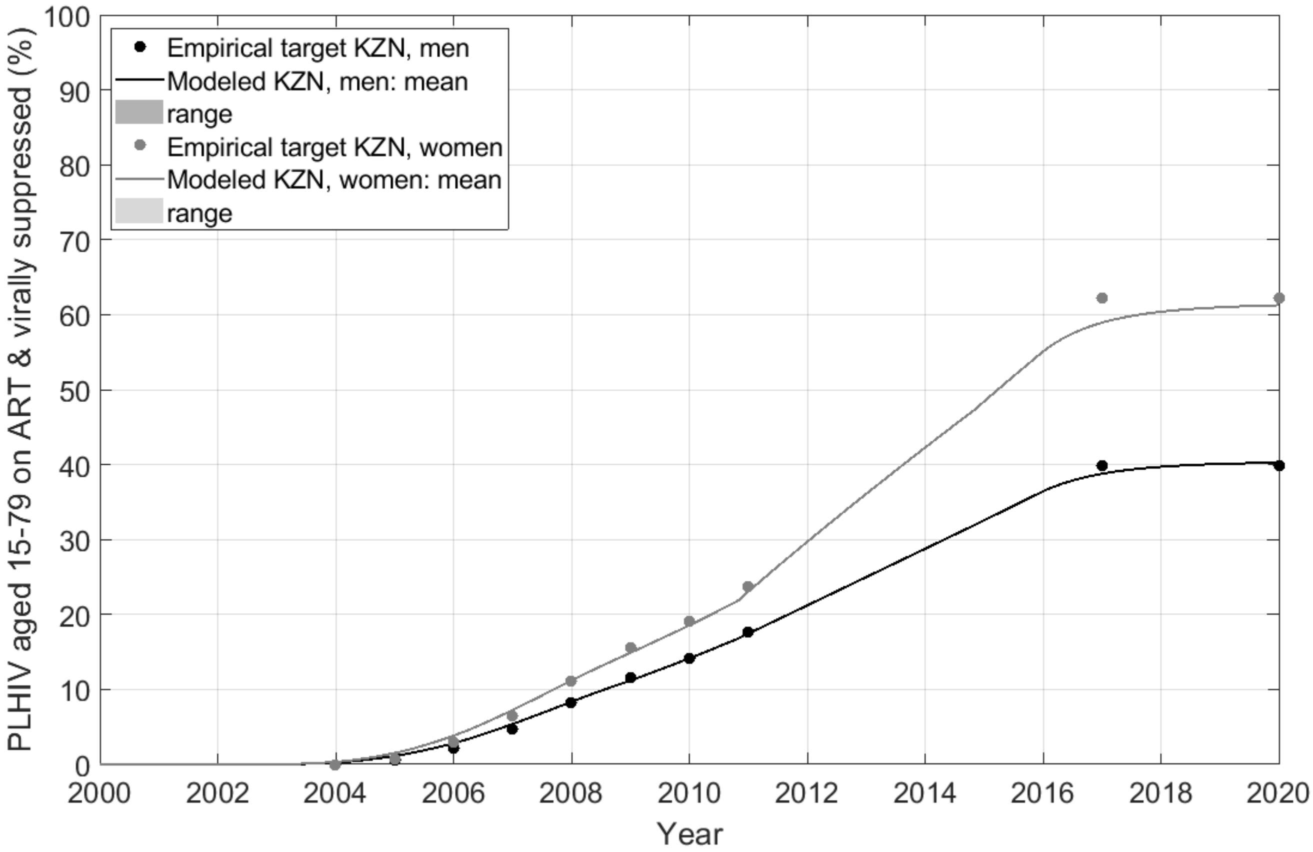

Figure C. Percentage of persons living with HIV on ART and virally suppressed over time. Shaded regions represent the range of estimates using the 25 best-fitting model parameter sets, although given that the percentage of persons with viral suppression was a model input, there is very little variability in this outcome.

(II.e.ii.) Condoms

Condom use is defined as the average proportion of the population ever using condoms multiplied by the percent of sexual acts for which those persons use a condom. Condom use is initiated in the model in 1995, scales up linearly until 2000, and then remains constant. We calibrate the level of condom use in 2000 (see Table N in section III.a.iii). We assume condoms reduce HIV acquisition in both men and women by 80%(44).

(II.e.iii.) Circumcision

We assume that men without HIV who receive voluntary medical male circumcision (VMMC) have decreased risk of HIV acquisition. Data suggest that men circumcised by a medical professional have a 60% lower risk of acquiring HIV(45, 46). We assume no effect of circumcision for men living with HIV (47) and that circumcision does not reduce the risk of HIV transmission to female partners(48, 49).

We model medical circumcision beginning in 1960 for age groups 15-19 and 20-24 (Table M). Prior to the initiation of the South Africa National VMMC program in 2010, circumcision was primarily targeted to young adult men as a rite of passage(50). In addition to accounting for the historical practice of circumcision(51, 52), starting circumcision in 1960 among youth results in circumcision prevalence among men ages 50 and older in 2012 corresponding to observed estimates(53). We assume coverage increases linearly to 2000 and between 2000 and 2008 to match coverage levels estimated from SABSSM data(50, 54). Following initiation of the national VMMC program in 2010, we model scale-up of circumcision for all men aged 15 or older at levels extrapolated backwards from 2012-2017 SABSSM and DHS data (1, 2, 53). Estimates of the proportion of men without HIV who are circumcised in 2020 are extrapolated from 2017 data assuming that coverage continues to increases linearly, as suggested by consistent VMMCs per year reported by UNAIDS between 2015 and 2019(55). We assume country-level VMMC coverage data is reflective of that in KwaZulu-Natal as the overall proportion of men who received VMMC is similar in SA and KZN(2, 53), and province-level estimates are not provided by age.

Table M. Proportion of men without HIV who receive VMMC(1, 2, 50, 53, 54). *Prior to 2010, the coverage of circumcision in men aged 25 and older slowly increases as men circumcised between ages 15-24 age into these groups. We begin tracking this in the model in 2010 to ensure that coverage matches observed targets, with additional men newly circumcised in these older ages if necessary. Estimates of the proportion of men without HIV circumcised in 2020 are extrapolated based on evidence that coverage continues to increase linearly from 2012.

**VMMC coverage among men without HIV over time by age group**				
	**Age group**			
**Year**	**15-19**	**20-24**	**25-49**	**50-79**
**1960**	0.040	0.060	*	*
**2000**	0.100	0.130	*	*
**2008**	0.114	0.161	*	*
**2010**	0.143	0.201	0.140	0.120
**2012**	0.172	0.242	0.191	0.143
**2017**	0.459	0.420	0.318	0.204
**2020**	0.631	0.527	0.394	0.241

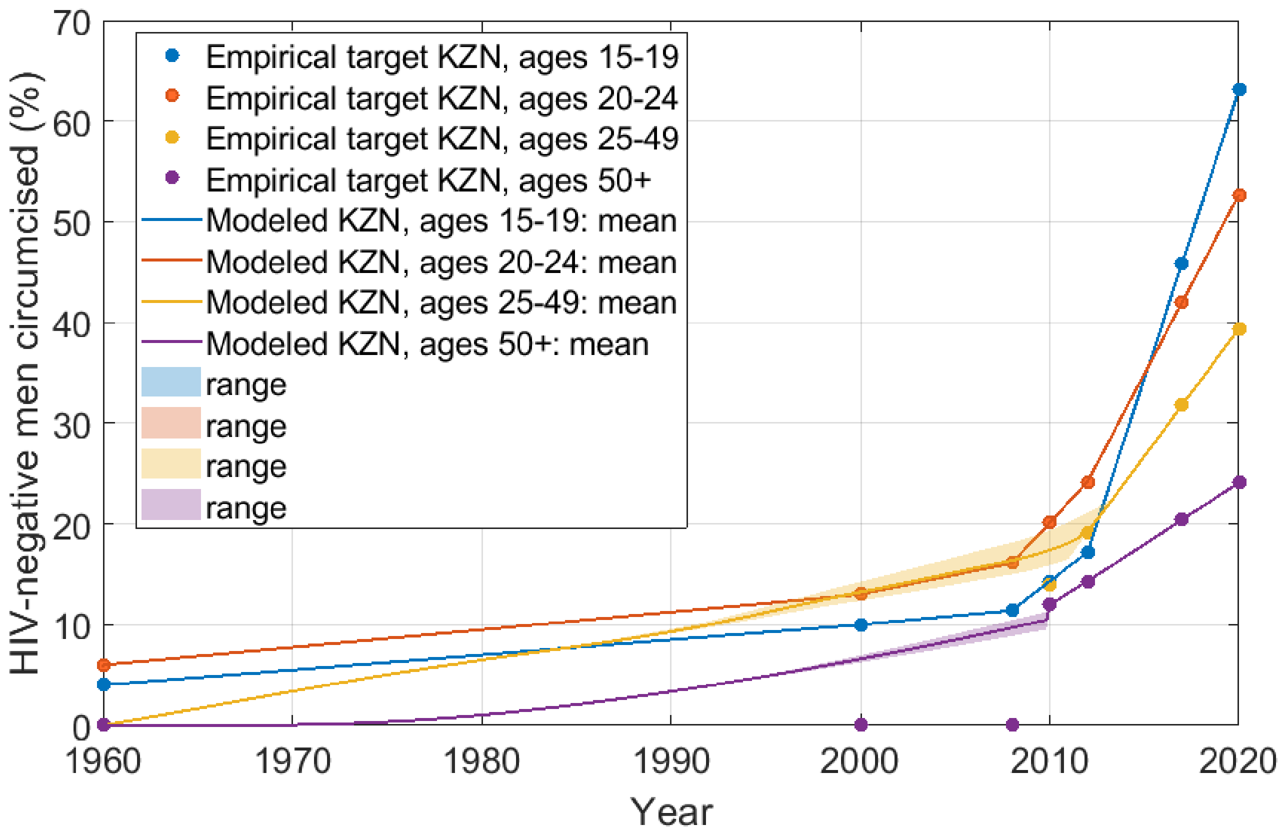

Figure D. Percentage of men without HIV who are circumcised over time. Shaded regions represent the range of estimates using the 25 best-fitting model parameter sets.

(I.f.) Future assumptions and interventions

(I.f.i.) Demography

For future simulations, we model a 50% linear decline in fertility rates from 2020 to 2035 to match projected United Nations Population Division estimates for population size, age distribution, and total fertility (4, 5).

(I.f.ii.) HIV antiretroviral therapy (ART)

Model scenarios differ in their assumptions about future scale-up of ART. In scenarios with standard-of-care clinic ART, the proportion of persons living with HIV who are virally suppressed remains at the estimated levels for 2020 (or 2017) for the duration of the simulation. In scenarios with additional HIV testing or treatment interventions, the percentage of persons living with HIV who are treated with viral suppression is assumed to increase in the first timestep of 2020.

(I.f.iii.) Home HIV testing and counseling campaigns (HTC)

In scenarios including additional home HIV testing and counseling (HTC) campaigns once every 5 years, we estimate and track the proportion of persons with HIV aged 15-79 who are diagnosed over time outside of the model compartments. At baseline in 2020, we assume that 88.9% of women and 78% of men are diagnosed(2). By assuming that each home HTC campaign has 75% testing coverage(56), and multiplying campaign coverage by the number of persons in the population who do not have HIV or who are living with HIV and undiagnosed, we are able to calculate the number of additional persons tested during each home HTC campaign and the number of persons with HIV who learn their status during each campaign.

We also adjust the number of persons diagnosed and undiagnosed over time according to model dynamics. We assign 59% of annual incidence HIV infections as diagnosed and 41% as undiagnosed based on observed data from KwaZulu-Natal indicating that 41% of persons refuse HIV testing within the year(57). Persons with HIV aging into the 15-19 age group are assumed to be undiagnosed, and persons aging out of the model at age 79 or dying due to background mortality are proportionately assigned as diagnosed or undiagnosed. We assume that persons with HIV will be diagnosed before end of life, and thus subtract all HIV-associated and cervical cancer-associated deaths from diagnosed persons with HIV.

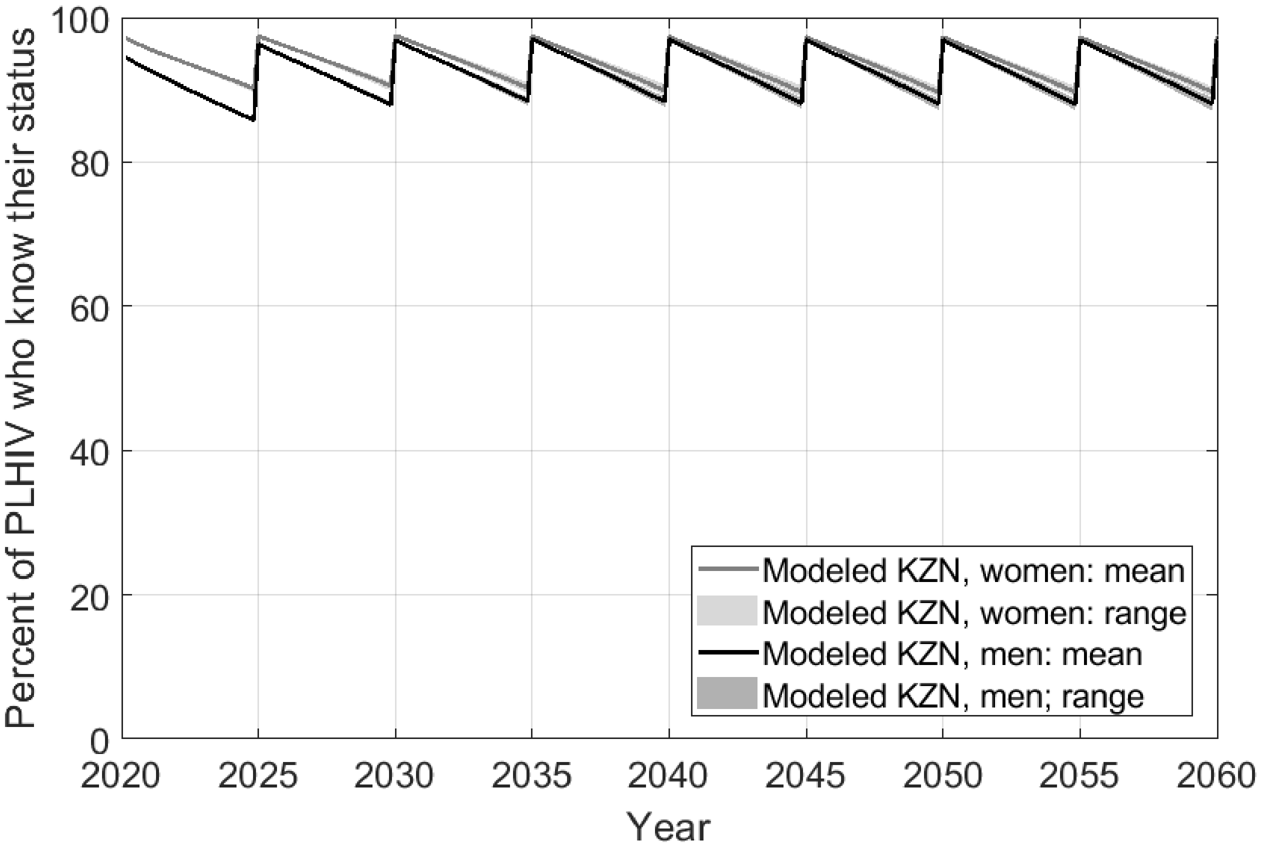

Figure E. Percentage of persons with HIV who know their HIV status over time with baseline testing and additional home HTC campaigns. Shaded regions represent the range of estimates using the 25 best-fitting model parameter sets.

(I.f.iv.) Circumcision

In modeled scenarios without future VMMC scale-up, we assume coverage remains at estimated levels for 2020 for the duration of the simulation. In scenarios in which KZN achieves 72% coverage for men aged 15-79 by 2030 as observed with enhanced community-based linkage strategies(58), we assume that coverage increases linearly between 2020 and 2030 and then remains stable.

(III.) Model calibration and validation

(III.a.) Calibration

We took a phased approach to model calibration. Briefly, we used hand-calibration to explore the sensitivity of model outcomes to individual parameters (Phase 0). This exploratory step informed our decision to divide the formal Bayesian calibration into two phases. In Phase 1, we used a Bayesian algorithm to fit 22 sexual behavior and HIV natural history parameters to observed demographic and HIV prevalence data. Randomly resampling from the 50 best-fitting parameter sets of Phase 1, we then used the same Bayesian algorithm to fit 23 HPV natural history parameters to observed data on HPV prevalence, CIN prevalence, cervical cancer incidence, and type distribution (Phase 2). The choice to retain 50 parameter sets from Phase 1 focused resampling to sets that best fit demographic and HIV prevalence data without overly restricting the parameter space for Phase 2. The number of retained sets was refined to 25 in Phase 2 to select sets that best fit all empirical targets while capturing underlying parameter uncertainty. 50 and 25 are in line with the number of sets retained at each stage of model fitting in prior literature(59), and maintain computational feasibility. The range of outcomes generated from the 25 best-fitting sets of Phase 2 and their corresponding resampled Phase 1 parameter values capture our uncertainty in model predictions, and represent the peak of the joint posterior distribution across parameters.

(III.a.i.) Phase 0

In greater detail, we first identified parameters for which empirical evidence was limited. We assumed a uniform prior distribution for each parameter, and we used published estimates and expert opinion to assign upper and lower bounds. By systematically varying parameters within the specified ranges, we verified that the parameters included in the calibration and their prior ranges could plausibly produce model results fitting the observed data. We also assessed the relative impact of individual parameters on different model outcomes. From this exploratory process, we determined that HIV outcomes are more sensitive to sexual behavior parameters than HPV-related outcomes, that HIV outcomes are tightly linked to demographic outcomes, and that pre-cancer and cancer outcomes are solely driven by the HPV natural history parameters. These findings allowed us to narrow prior ranges and divide our formal Bayesian calibration into two Phases, thereby increasing efficiency. To further increase efficiency, we fixed parameters with negligible impact on all model outcomes to the most robust estimates from literature.

(III.a.ii.) Bayesian calibration approach

For Phases 1 and 2, we used the Approximate Bayesian Computation-Sequential Monte Carlo (ABC-SMC) algorithm to efficiently explore the multidimensional parameter space and identify parameter sets that generated model outcomes that best fit observed data(60-62). The ABC-SMC approach focuses sampling in the highest likelihood areas and avoids resampling the same parameter sets. Because a Bayesian calibration approach frames parameters as probabilistic and unknown, this allowed us to systematically estimate the range of outcomes given uncertainty in the underlying parameters.

We used the summed-log-likelihood as our metric of model fit, and maximum likelihood estimation (MLE) to evaluate the probability that potential parameter sets produced observed data. The point in multidimensional parameter space that maximizes the likelihood function is the most likely to have produced the observed trends in transmission of infection and development of disease. (Note: although ABC-SMC is an “approximate” Bayesian method in that it doesn’t require computation of a likelihood, we used the summed-log-likelihood as our fit metric.)

(III.a.iii.) Phase 1

For the first ABC-SMC iteration, we fit 22 sexual behavior and HIV natural history parameters (Table N) to observed demographic and HIV prevalence data (Table O). We sampled a total of 91,980 parameter sets, from which we took the range of values for individual parameters from among the 50 best-fitting sets to define parameter uncertainty.

(III.a.iv.) Phase 2

We then used ABC-SMC to fit 23 HPV transmission, progression, regression, and immunity natural history parameters to observed data on HPV prevalence, CIN prevalence, cervical cancer incidence, and type distribution(63-74). For each Phase 2 simulation, we randomly resampled the sexual behavior and HIV natural history parameters from the 50 best-fitting parameter sets of Phase 1. After sampling a total of 25,760 parameter sets, the range of individual parameters among the 25 best-fitting Phase 2 sets and their corresponding Phase 1 sampled values defined parameter uncertainty.

Table N: Phase 1 calibrated sexual behavior and HIV natural history parameters. Values presented are the Mean [Uncertainty Range] of the 50 best-fitting parameter sets after Phase 1, and the Mean [Uncertainty Range] of the Phase 1 values corresponding to the 25 best-fitting parameter sets after Phase 2.

	Parameter	Sexual risk group	Age group	Prior [lb, ub]	Phase 1 Mean [Uncertainty Range]	Phase 2 Mean [Uncertainty Range]	References
1	Men, annual partnerships	High	15-19	0.20, 9.15	2.95 [0.25, 7.64]	2.23 [0.27, 7.50]	Prior bounds were defined around observed data on male partnerships (14), with ranges informed by Phase 0. Partnerships for the 10-14 age group were set as 0.5x calibrated partnerships in the 15-19 age group.
2			20-24	5.00, 18.30	10.88 [5.02, 18.23]	12.33 [5.02, 17.32]	
3			25-29	6.25, 37.50	24.76 [7.45, 37.35]	21.28 [8.99, 35.22]	
4			30-44	6.25, 37.50	19.76 [7.43, 36.89]	20.32 [7.89, 34.78]	
5			45-79	5.00, 18.00	12.11 [5.24, 17.83]	13.85 [5.24, 17.83]	
6		Moderate risk multiplier on high risk	15-79	0.11, 0.87	0.15 [0.11, 0.29]	0.16 [0.11, 0.29]	Prior range informed by observed estimates and Phase 0.
7		Low risk multiplier on moderate risk	15-79	0.15, 0.75	0.27 [0.16, 0.59]	0.24 [0.16, 0.52]	
8	Women, annual partnerships	High	15-19	2.25, 13.50	9.39 [3.37, 13.36]	10.07 [5.92, 13.34]	Prior bounds were defined around observed data on female partnerships (14), with ranges informed by Phase 0. Partnerships for the 10-14 age group were set as 0.5x calibrated partnerships in the 15-19 age group.
9			20-24	6.50, 39.00	27.20 [7.18, 38.19]	25.84 [13.18, 37.52]	
10			25-29	5.00, 28.50	13.09 [5.27, 28.28]	10.70 [5.21, 26.69]	
11			30-44	5.00, 21.00	8.29 [5.09, 15.42]	8.58 [5.31, 14.51]	
12			45-79	5.00, 15.00	10.50 [5.35, 14.89]	9.33 [5.65, 14.89]	
13		Moderate risk multiplier	15-79	0.11, 0.87	0.51 [0.18, 0.86]	0.47 [0.18, 0.86]	Prior range informed by observed

		**on high risk**					estimates and Phase 0.
**14**		**Low risk multiplier on moderate risk**	**15-79**	0.15, 0.75	0.49 [0.24, 0.74]	0.49 [0.24, 0.73]	
**15**	**Women, annual acts per partnership**	**Low**	**15-19**	53.13, 88.56	67.65 [53.68, 87.50]	65.71 [58.17, 76.37]	Prior range informed by Phase 0. Acts per partnership for the 10-14 age group were set as 0.5x the 15-19 age group. We assume acts per partnership for moderate and high-risk individuals are 0.60x acts in the next lowest risk group.<br><br>Phase 0 demonstrated that fit to observed HPV and CIN2/3 prevalence by age was sensitive to age trends in female acts. Among men, we assume those aged 10-19 have the same number of acts per partnership as women, whereas men aged 20-79 have equal acts to women of the next lowest age group, reflecting age disparities in relationships.
**16**			**20-24**	52.47, 87.44	71.46 [53.73, 86.02]	72.94 [54.24, 84.97]	
**17**			**25-29**	42.15, 70.25	56.07 [44.64, 69.02]	55.39 [47.20, 62.32]	
**18**			**30-44**	40.95, 68.25	49.13 [41.04, 62.07]	49.22 [41.04, 59.50]	
**19**			**45-79**	39.53, 65.88	52.82 [39.59, 65.05]	54.02 [42.06, 64.40]	

**20**	**Condom use in 2000 (x% of people * y% of the time)**	0.11, 0.45	0.29 [0.11, 0.45]	0.28 [0.16, 0.40]	Prior bounds were informed by data on self-reported condom use for contraception (1). The lower bound uses data from married women, and the upper bound uses data from women aged 20-24, who were the most sexually active.
**21**	**Mixing parameter by age** (0 < ϵ_a < 1), where ($\epsilon_a = 0$) indicates completely off-diagonal mixing, and ($\epsilon_a = 1$) indicates completely random mixing	0.10, 0.60	0.46 [0.13, 0.59]	0.43 [0.13, 0.56]	Prior set according to expert opinion (Ruanne Barnabas, personal communications) and published literature(75, 76) indicating that mixing by age is more assortative than random.
**22**	**HIV transmission rate per act**	0.0004, 0.0020	0.0008 [0.0006, 0.0013]	0.0009 [0.0007, 0.0013]	Prior set according to data on female-to-male and male-to-female transmission in developed and developing countries (31), and narrowed in Phase 0 to exclude values inconsistent with observed cohort data.

Table O: Phase 1 targets

Phase 1: Demography calibration targets								
**Criteria**	**Gender**	**Year**	**Age Group**	**Occurrences**	**N**	**Mean**	**Variance**	**Reference**
**Total population size**	**Men and women combined**	**2001**	**0-79**			9348178	4.06E+10	Statistics South Africa Census 2001 Post-enumeration survey, Table 2.7: Adjusted total population- full universe (77)
	**Men and women combined**	**2011**	**0-79**			10159121	1.21E+10	Statistics South Africa Census 2011 Post-enumeration survey, Table 14: Adjusted total population- full universe (78)
	**Men and women combined**	**2019**	**0-79**			11192662	1.21E+10	
**Criteria**	**Gender**	**Year**	**Age Group**	**Occurrences**	**N**	**Mean**	**Variance**	**Reference**
**Population age distribution**	**Men and women combined**	**1996**	**0-4**	964546	8244075	0.1170	1.25E-08	Statistics South Africa Primary tables Census '96 and 2001 compared, Table 4.1: KwaZulu-Natal (79)
			**5-9**	1005944	8244075	0.1220	1.30E-08	
			**10-14**	1018218	8244075	0.1235	1.31E-08	
			**15-19**	914305	8244075	0.1109	1.20E-08	
			**20-24**	851953	8244075	0.1033	1.12E-08	
			**25-29**	684675	8244075	0.0831	9.24E-09	
			**30-34**	590583	8244075	0.0716	8.07E-09	
			**35-39**	504746	8244075	0.0612	6.97E-09	
			**40-44**	408926	8244075	0.0496	5.72E-09	
			**45-49**	337933	8244075	0.0410	4.77E-09	
			**50-54**	248877	8244075	0.0302	3.55E-09	
			**55-59**	212751	8244075	0.0258	3.05E-09	
			**60-64**	178471	8244075	0.0216	2.57E-09	
			**65-69**	158208	8244075	0.0192	2.28E-09	
			**70-74**	95372	8244075	0.0116	1.39E-09	
			**75-79**	68567	8244075	0.0083	1.00E-09	
**Population age distribution**	**Men and women combined**	**2011**	**0-4**	1198134	10159121	0.1179	1.02E-08	Statistics South Africa Statistical Release P0301 Community Survey 2016, Table 2.1: Census 2011 and Community Survey 2016 (80)
			**5-9**	1042528	10159121	0.1026	9.06E-09	
			**10-14**	1038857	10159121	0.1023	9.04E-09	
			**15-19**	1119535	10159121	0.1102	9.65E-09	
			**20-24**	1102388	10159121	0.1085	9.52E-09	
			**25-29**	980929	10159121	0.0966	8.59E-09	
			**30-34**	729230	10159121	0.0718	6.56E-09	
			**35-39**	612615	10159121	0.0603	5.58E-09	
			**40-44**	499102	10159121	0.0491	4.60E-09	
			**45-49**	454637	10159121	0.0448	4.21E-09	
			**50-54**	384397	10159121	0.0378	3.58E-09	

			**55-59**	325571	10159121	0.0320	3.05E-09	
			**60-64**	271326	10159121	0.0267	2.56E-09	
			**65-69**	175673	10159121	0.0173	1.67E-09	
			**70-74**	137821	10159121	0.0136	1.32E-09	
			**75-79**	86378	10159121	0.0085	8.30E-10	
**Population age distribution**	**Men and women combined**	**2019**	**0-4**	1231101	11192662	0.1100	8.75E-09	Statistics South Africa Statistical Release P0302 2019 Mid-year population estimates, Table 6 and Table 11 (5)
			**5-9**	1196909	11192662	0.1069	8.53E-09	
			**10-14**	1136163	11192662	0.1015	8.15E-09	
			**15-19**	980573	11192662	0.0876	7.14E-09	
			**20-24**	1006031	11192662	0.0899	7.31E-09	
			**25-29**	1061072	11192662	0.0948	7.67E-09	
			**30-34**	1008573	11192662	0.0901	7.33E-09	
			**35-39**	805405	11192662	0.0720	5.97E-09	
			**40-44**	613052	11192662	0.0548	4.63E-09	
			**45-49**	517032	11192662	0.0462	3.94E-09	
			**50-54**	423932	11192662	0.0379	3.26E-09	
			**55-59**	376538	11192662	0.0336	2.90E-09	
			**60-64**	306529	11192662	0.0274	2.38E-09	
			**65-69**	244864	11192662	0.0219	1.91E-09	
			**70-74**	178784	11192662	0.0160	1.40E-09	
			**75-79**	106104	11192662	0.0095	8.39E-10	

**Phase 1: HIV calibration targets**								
**Criteria**	**Gender**	**Year**	**Age Group**	**Cases**	**N**	**Mean**	**Variance**	**Reference**
**HIV Prevalence in Men**	**Men**	**2003**	**15-19**	57	5624	0.0101	1.78E-06	Africa Centre cohort (now on AHRI) data request (14)
			**20-24**	455	5141	0.0885	1.57E-05	
			**25-29**	1027	3643	0.2819	5.56E-05	
			**30-34**	1253	2773	0.4519	8.93E-05	
			**35-39**	728	1956	0.3722	1.19E-04	
			**40-44**	441	1638	0.2692	1.20E-04	
			**45-49**	309	1362	0.2269	1.29E-04	
	**Men**	**2005**	**15-19**	74	5624	0.0132	2.31E-06	
			**20-24**	519	5141	0.1010	1.77E-05	
			**25-29**	1214	3643	0.3332	6.10E-05	
			**30-34**	1243	2773	0.4483	8.92E-05	
			**35-39**	707	1956	0.3615	1.18E-04	
			**40-44**	527	1638	0.3217	1.33E-04	
			**45-49**	284	1362	0.2085	1.21E-04	
	**Men**	**2006**	**15-19**	41	5624	0.0073	1.29E-06	
			**20-24**	466	5141	0.0906	1.60E-05	

			25-29	1139	3643	0.3127	5.90E-05	
			30-34	1175	2773	0.4237	8.81E-05	
			35-39	815	1956	0.4167	1.24E-04	
			40-44	533	1638	0.3254	1.34E-04	
			45-49	394	1362	0.2893	1.51E-04	
HIV Prevalence in Men	Men	2007	15-19	58	5624	0.0103	1.81E-06	
			20-24	552	5141	0.1074	1.86E-05	
			25-29	1106	3643	0.3036	5.80E-05	
			30-34	1116	2773	0.4025	8.67E-05	
			35-39	785	1956	0.4013	1.23E-04	
			40-44	648	1638	0.3956	1.46E-04	
			45-49	393	1362	0.2885	1.51E-04	
	Men	2008	15-19	58	5624	0.0103	1.81E-06	
			20-24	557	5141	0.1083	1.88E-05	
			25-29	1124	3643	0.3085	5.86E-05	
			30-34	1079	2773	0.3891	8.57E-05	
			35-39	911	1956	0.4657	1.27E-04	
			40-44	500	1638	0.3053	1.29E-04	
			45-49	370	1362	0.2717	1.45E-04	
	Men	2009	15-19	52	5624	0.0092	1.63E-06	
			20-24	403	5141	0.0784	1.41E-05	
			25-29	1010	3643	0.2772	5.50E-05	
			30-34	1271	2773	0.4583	8.95E-05	
			35-39	1081	1956	0.5527	1.26E-04	
			40-44	574	1638	0.3504	1.39E-04	
			45-49	498	1362	0.3656	1.70E-04	
**Criteria**	**Gender**	**Year**	**Age Group**	**Cases**	**N**	**Mean**	**Variance**	**Reference**
HIV Prevalence in Women	Women	2003	15-19	555	5622	0.0987	1.58E-05	Africa Centre cohort (now on AHRI) data request (14)
			20-24	1797	5489	0.3274	4.01E-05	
			25-29	1957	3869	0.5058	6.46E-05	
			30-34	1502	3174	0.4732	7.85E-05	
			35-39	844	2404	0.3511	9.48E-05	
			40-44	540	2075	0.2602	9.28E-05	
			45-49	387	1829	0.2116	9.12E-05	
	Women	2005	15-19	431	5622	0.0767	1.26E-05	
			20-24	1794	5489	0.3268	4.01E-05	
			25-29	1942	3869	0.5019	6.46E-05	
			30-34	1428	3174	0.4499	7.80E-05	
			35-39	909	2404	0.3781	9.78E-05	
			40-44	522	2075	0.2516	9.07E-05	
			45-49	370	1829	0.2023	8.82E-05	
	Women	2006	15-19	489	5622	0.0870	1.41E-05	

			**20-24**	1732	5489	0.3155	3.93E-05	
			**25-29**	1804	3869	0.4663	6.43E-05	
			**30-34**	1505	3174	0.4742	7.86E-05	
			**35-39**	875	2404	0.3640	9.63E-05	
			**40-44**	530	2075	0.2554	9.17E-05	
			**45-49**	320	1829	0.1750	7.89E-05	
**HIV Prevalence in Women**	**Women**	**2007**	**15-19**	527	5622	0.0937	1.51E-05	
			**20-24**	1793	5489	0.3267	4.01E-05	
			**25-29**	2022	3869	0.5226	6.45E-05	
			**30-34**	1527	3174	0.4811	7.87E-05	
			**35-39**	898	2404	0.3735	9.73E-05	
			**40-44**	698	2075	0.3364	1.08E-04	
			**45-49**	361	1829	0.1974	8.66E-05	
	**Women**	**2008**	**15-19**	531	5622	0.0945	1.52E-05	
			**20-24**	1697	5489	0.3092	3.89E-05	
			**25-29**	1993	3869	0.5151	6.46E-05	
			**30-34**	1587	3174	0.5000	7.88E-05	
			**35-39**	968	2404	0.4027	1.00E-04	
			**40-44**	701	2075	0.3378	1.08E-04	
			**45-49**	491	1829	0.2685	1.07E-04	
	**Women**	**2009**	**15-19**	621	5622	0.1105	1.75E-05	
			**20-24**	1794	5489	0.3268	4.01E-05	
			**25-29**	1896	3869	0.4900	6.46E-05	
			**30-34**	1692	3174	0.5331	7.84E-05	
			**35-39**	1071	2404	0.4455	1.03E-04	
			**40-44**	778	2075	0.3749	1.13E-04	
			**45-49**	495	1829	0.2706	1.08E-04	

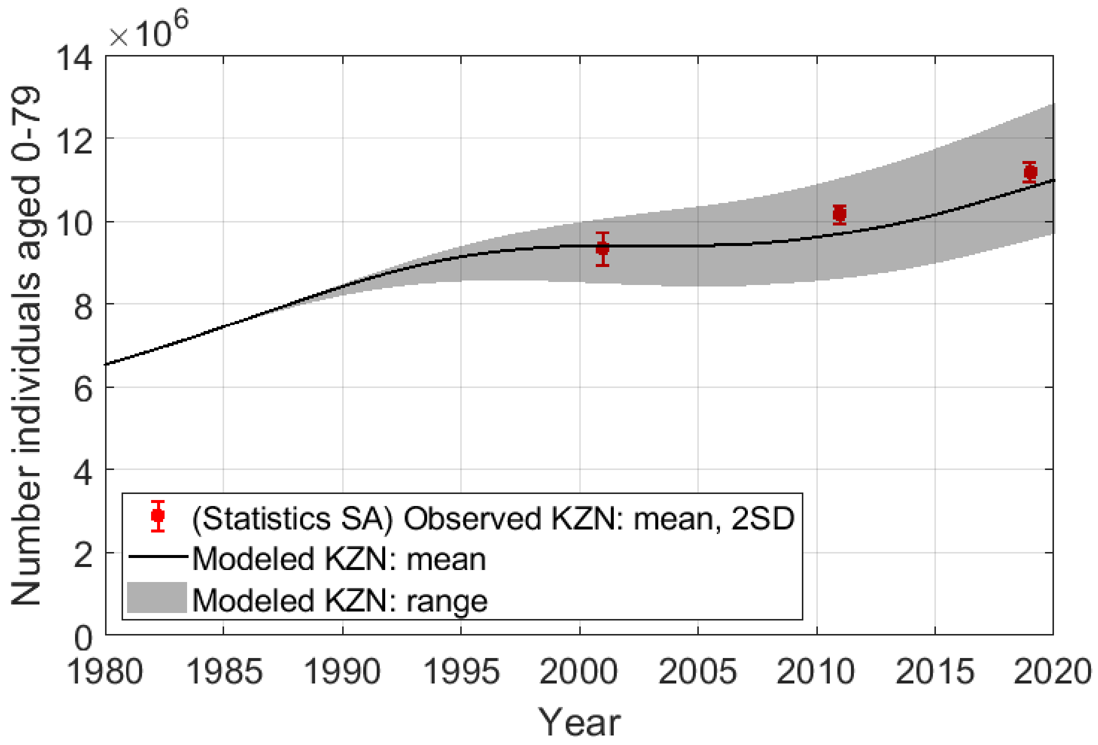

Figure F. Model fit to observed total population size data over time. Standard deviation of the observed data calculated from the given absolute error assuming that the total population size follows a normal distribution. Shaded region represents the range of estimates using the 25 best-fitting model parameter sets.

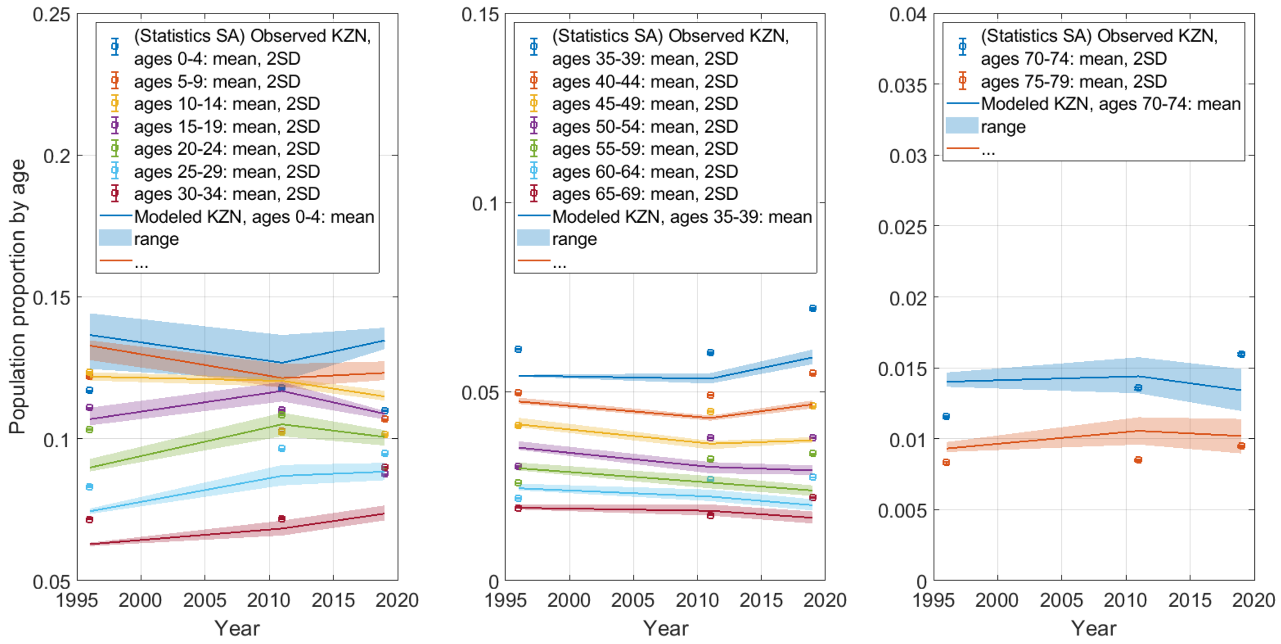

Figure G. Model fit to observed population age distribution data over time. Standard deviation of the observed data calculated assuming that population proportions follow a normal approximation of the binomial distribution. Shaded regions represent the range of estimates using the 25 best-fitting model parameter sets.

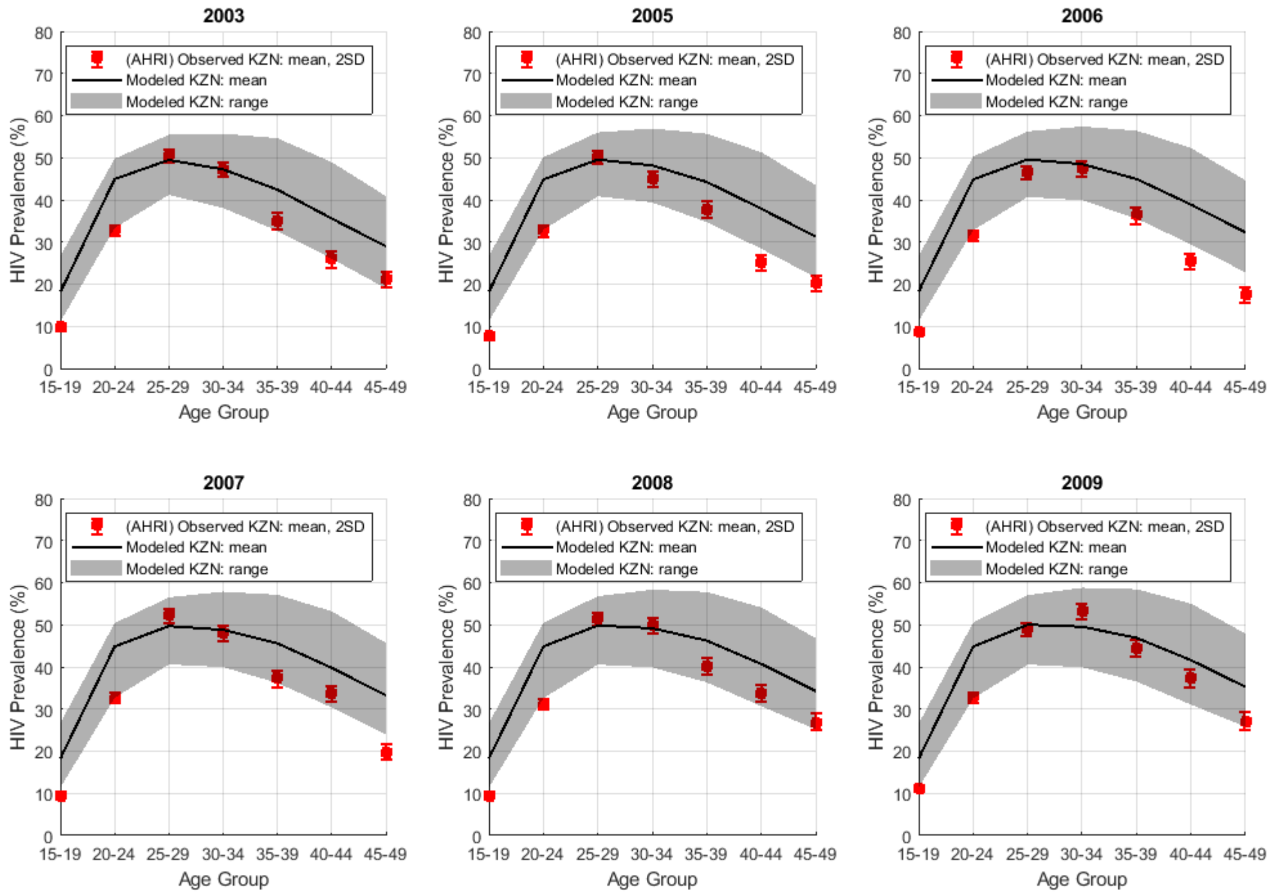

Figure H. Model fit to observed HIV prevalence data in women by age over time. Standard deviation of the observed data calculated assuming that prevalence proportions follow a normal approximation of the binomial distribution. Shaded regions represent the range of estimates using the 25 best-fitting model parameter sets.

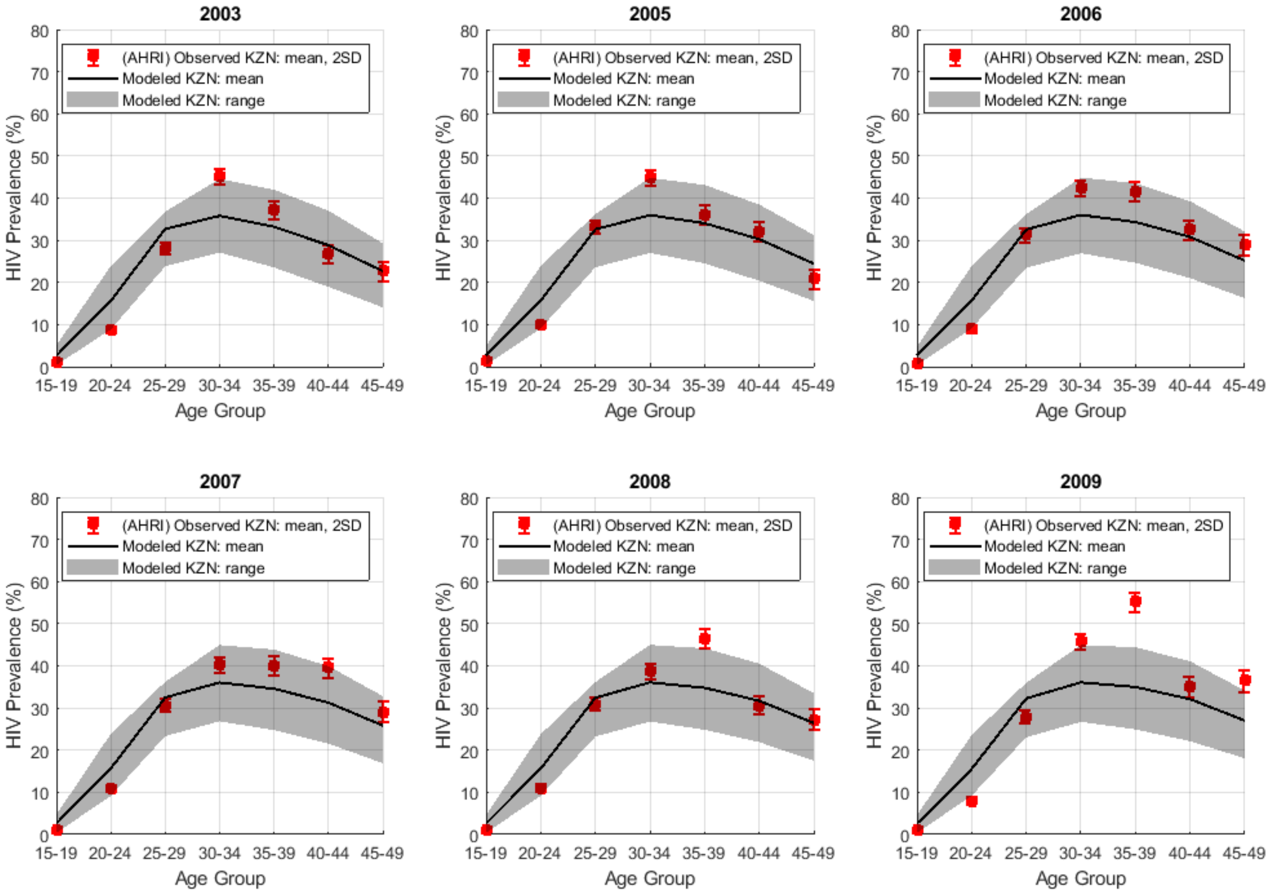

Figure I. Model fit to observed HIV prevalence data in men by age over time. Standard deviation of the observed data calculated assuming that prevalence proportions follow a normal approximation of the binomial distribution. Shaded regions represent the range of estimates using the 25 best-fitting model parameter sets.

(III.a.v.) Approximate Bayesian Computation-Sequential Monte Carlo (ABC-SMC) algorithm

The ABC-SMC algorithm repeats the following steps until approximate convergence is achieved(60-62):

1. Sample the parameter space
2. Simulate model outputs for each potential parameter set
3. Calculate a likelihood for each parameter set
4. Retain parameter sets with the highest likelihood (that produce model outputs most similar to observed data)
5. Weight the retained parameter sets as the inverse of their probability of having been sampled
6. Re-sample from the retained parameter sets according to their weights, and then use a Gaussian perturbation kernel to avoid re-sampling the exact same sets.

Essentially, we sequentially sample the parameter space, and then use maximum likelihood estimation to identify the parameter sets that are most likely to have generated the observed data. At each iteration we retain only a proportion (*alpha*) of the best-fitting sets and then re-sample from those sets, therefore focusing sampling in the areas of highest likelihood. By assigning weights to the parameter sets in the re-sampling pool, we ensure thorough exploration of the parameter space. Eventually, the probability density distribution of accepted parameter sets converges on the posterior probability distribution of the parameters. Convergence is achieved when less than a set proportion (p_{acc_min}) of accepted particles have a likelihood greater than *epsilon,* or the smallest likelihood of the accepted particles from the last iteration. Smaller values of *alpha* and p_{acc_min} improve the quality of the final posterior approximation but increase the total number of model runs that will be needed. To balance quality with computational time, we choose intermediate values of *alpha* = 0.6 (phase 1) or 0.4 (phase 2), and p_{acc_min} = 0.1.

(III.a.vi.) Likelihood calculation

The model was calibrated to fit observed data on HIV, HPV, and population dynamics from 1996 to 2019 (Table O). We defined likelihood equations for each target. By assuming that all outputs followed independent normal distributions, we were able to use the same equation form for all targets. For prevalence data, we assumed a normal approximation of the binomial where μ = prevalence proportion (p) and variance = (p(1-p))/N for a sample size N. For incidence data, we assumed a normal approximation of a Poisson distribution where μ = λ, variance = λ, and λ = the annual CC incidence rate. We assumed a normal distribution for the total population size of KwaZulu-Natal and that the 2019 estimate had the same variance as the 2011 estimate. We believe this is conservative as trends indicate that variance will continue to

decrease as sampling techniques improve. In most cases, mean values were derived from a single study. However, the proportions of vaccine and non-vaccine type HPV by disease stage were averaged across several studies. Therefore, we assumed a normal approximation of the binomial and calculated the variance of an average as = (1/*N*)^2 * (sum of the individual variances). When observed values were estimated from a multi-year sample, we compared to modeled results from the year at the middle of the time interval.

**Equation variables**	
n	The number of outcomes, or calibration targets
μ	Vector of observed outcome means of length n
σ^2	Vector of observed outcome variances of length n
x	Vector of model-generated outcome means of length n

Assuming a normal distribution, we calculated the likelihood of the n observed outcomes with mean μ and variance σ^2 given the model-generated set of means x as:

$$L(\mu, \sigma^2|x) = (2\pi\sigma^2)^{\frac{-n}{2}} \cdot exp\left(-\frac{1}{2\sigma^2}\sum_{i=1}^{n}(x_i - \mu)^2\right)$$

However, it is often more convenient to work with the natural logarithm of the likelihood function, or the log-likelihood:

$$l\,n\big(L(\mu, \sigma^2|x)\big) = -\frac{n}{2} \cdot ln(2\pi\sigma^2) - \frac{1}{2\sigma^2}\sum_{i=1}^{n}(x_i - \mu)^2$$

Since $L(\mu, \sigma^2|x)$ and $l\,n\big(L(\mu, \sigma^2|x)\big)$ are monotonically related, we maximize $l\,n\big(L(\mu, \sigma^2|x)\big)$ by searching for (μ, σ^2) such that:

$$\nabla\, l\,n\big(L(\mu, \sigma^2|x)\big) = 0$$

(III.b.) Validation

To verify our ability to accurately predict future outcomes, we validated our HIV natural history module by comparing model output to additional time points and data sources (Table P). More specifically, we compared our model outputs to estimated HIV prevalence at later time points by gender and age from the same reference as our calibration data set (14). We also compared our modeled HIV prevalence for both genders combined in broad age groups to observed data for KZN from the 2017 SABSSM survey (2). Finally, we compared HIV incidence, a non-calibrated output, to observed data from the Africa Centre cohort in KZN, the same cohort captured by our prevalence data (81). Alignment of our model to these external data supports the robustness of our predicted HIV dynamics.

Table P. Validation targets.

Validation: HIV prevalence targets by gender							
**Criteria**	**Gender**	**Year**	**Age Group**	**Cases**	**N**	**Mean**	**References**
HIV Prevalence in Men	Men	2010	15-19	17	1060	0.0160	Africa Centre cohort (now on AHRI) data request (14)
			20-24	52	544	0.0956	
			25-29	69	238	0.2899	
			30-34	79	170	0.4647	
			35-39	77	148	0.5203	
			40-44	58	139	0.4173	
			45-49	48	131	0.3664	
	Men	2011	15-19	17	917	0.0185	
			20-24	41	511	0.0802	
			25-29	57	260	0.2192	
			30-34	77	173	0.4451	
			35-39	70	158	0.4430	
			40-44	49	118	0.4153	
			45-49	49	132	0.3712	
	Men	2012	15-19	23	837	0.0275	
			20-24	39	395	0.0987	
			25-29	52	209	0.2488	
			30-34	62	157	0.3949	
			35-39	63	127	0.4961	
			40-44	50	97	0.5155	
			45-49	34	103	0.3301	
HIV Prevalence in Men	Men	2013	15-19	39	1128	0.0346	
			20-24	47	487	0.0965	
			25-29	74	248	0.2984	
			30-34	85	180	0.4722	
			35-39	95	150	0.6333	
			40-44	63	122	0.5164	
			45-49	46	115	0.4000	
	Men	2014	15-19	28	975	0.0287	
			20-24	51	430	0.1186	
			25-29	80	226	0.3540	
			30-34	89	192	0.4635	
			35-39	73	142	0.5141	
			40-44	79	133	0.5940	
			45-49	45	111	0.4054	
	Men	2015	15-19	53	1343	0.0395	
			20-24	43	598	0.0719	
			25-29	94	340	0.2765	

			30-34	122	293	0.4164	
			35-39	89	171	0.5205	
			40-44	88	167	0.5269	
			45-49	69	155	0.4452	
	Men	2016	15-19	43	956	0.0450	
			20-24	34	424	0.0802	
			25-29	65	238	0.2731	
			30-34	85	202	0.4208	
			35-39	76	148	0.5135	
			40-44	65	127	0.5118	
			45-49	48	92	0.5217	

Criteria	Gender	Year	Age Group	Cases	N	Mean	References
HIV Prevalence in Men	Women	2010	15-19	114	1227	0.0929	Africa Centre cohort (now on AHRI) data request (14)
			20-24	289	920	0.3141	
			25-29	375	704	0.5327	
			30-34	303	512	0.5918	
			35-39	265	491	0.5397	
			40-44	187	438	0.4269	
			45-49	184	569	0.3234	
	Women	2011	15-19	95	1053	0.0902	
			20-24	255	805	0.3168	
			25-29	316	611	0.5172	
			30-34	281	458	0.6135	
			35-39	245	453	0.5408	
			40-44	193	446	0.4327	
			45-49	166	484	0.3430	
	Women	2012	15-19	97	928	0.1045	
			20-24	201	656	0.3064	
			25-29	253	498	0.5080	
			30-34	210	358	0.5866	
			35-39	211	359	0.5877	
			40-44	154	340	0.4529	
			45-49	143	365	0.3918	
HIV Prevalence in Men	Women	2013	15-19	119	1275	0.0933	
			20-24	275	810	0.3395	
			25-29	308	600	0.5133	
			30-34	331	510	0.6490	
			35-39	295	453	0.6512	
			40-44	206	419	0.4916	
			45-49	175	422	0.4147	
	Women	2014	15-19	114	1099	0.1037	

			20-24	264	764	0.3455	
			25-29	321	618	0.5194	
			30-34	336	537	0.6257	
			35-39	299	458	0.6528	
			40-44	230	424	0.5425	
			45-49	189	392	0.4821	
	Women	2015	15-19	171	1554	0.1100	
			20-24	360	1055	0.3412	
			25-29	468	867	0.5398	
			30-34	506	782	0.6471	
			35-39	397	614	0.6466	
			40-44	332	589	0.5637	
			45-49	289	583	0.4957	
	Women	2016	15-19	107	1144	0.0935	
			20-24	258	772	0.3342	
			25-29	348	664	0.5241	
			30-34	400	634	0.6309	
			35-39	310	463	0.6695	
			40-44	258	421	0.6128	
			45-49	217	432	0.5023	

Validation: HIV prevalence targets, genders combined			
**Criteria**	**Year**	**Mean [95% CI]**	**References**
HIV Prevalence, ages 15-49	2010	0.2904	Africa Centre cohort (now on AHRI) data request (14)
	2011	0.2905	
	2012	0.2932	
	2013	0.3119	
	2014	0.3381	
	2015	0.3382	
	2016	0.3445	
	2002	0.1570 [0.1160, 0.2110]	(2)
	2005	0.2190 [0.1830, 0.2590]	
	2008	0.2580 [0.2210, 0.2980]	
	2012	0.2790 [0.2520, 0.3080]	
	2017	0.2700 [0.2390, 0.3040]	
HIV Prevalence, ages 25+	2002	0.1490 [0.1010, 0.2150]	(2)
	2005	0.2050 [0.1680, 0.2460]	
	2008	0.2350 [0.1970, 0.2780]	
	2012	0.3010 [0.2690, 0.3360]	
	2017	0.3120 [0.2760, 0.3500]	
HIV Prevalence, ages 50+	2002	0.1100 [0.0450, 0.2430]	(2)
	2005	0.0950 [0.0590, 0.1480]	
	2008	0.0610 [0.0370, 0.1010]	
	2012	0.0980 [0.0740, 0.1280]	
	2017	0.1790 [0.1390, 0.2280]	

Validation: HIV incidence targets by gender			
**Criteria**	**Year**	**Mean [95% CI]**	**References**
Men aged 15-29, HIV incidence	2005-2017	Values digitized from reference	(81)
Men aged 30-54, HIV incidence	2005-2017	Values digitized from reference	
Men aged 15-54, HIV incidence	2005-2017	Values digitized from reference	
Women aged 15-29, HIV incidence	2005-2017	Values digitized from reference	
Women aged 30-49, HIV incidence	2005-2017	Values digitized from reference	
Women aged 15-49, HIV incidence	2005-2017	Values digitized from reference	

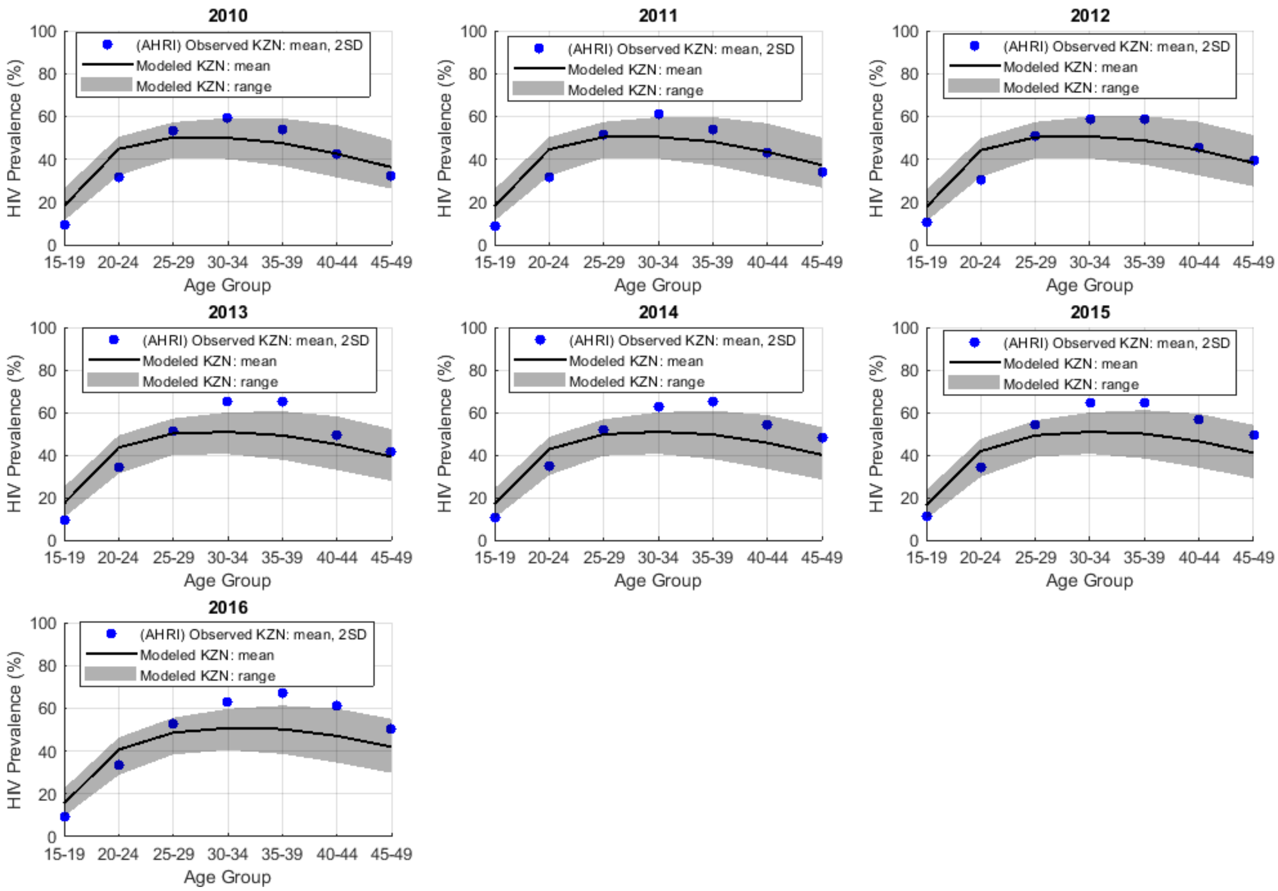

Figure J. Model validation to observed HIV prevalence data in women by age over time. Shaded regions represent the range of estimates using the 25 best-fitting model parameter sets.

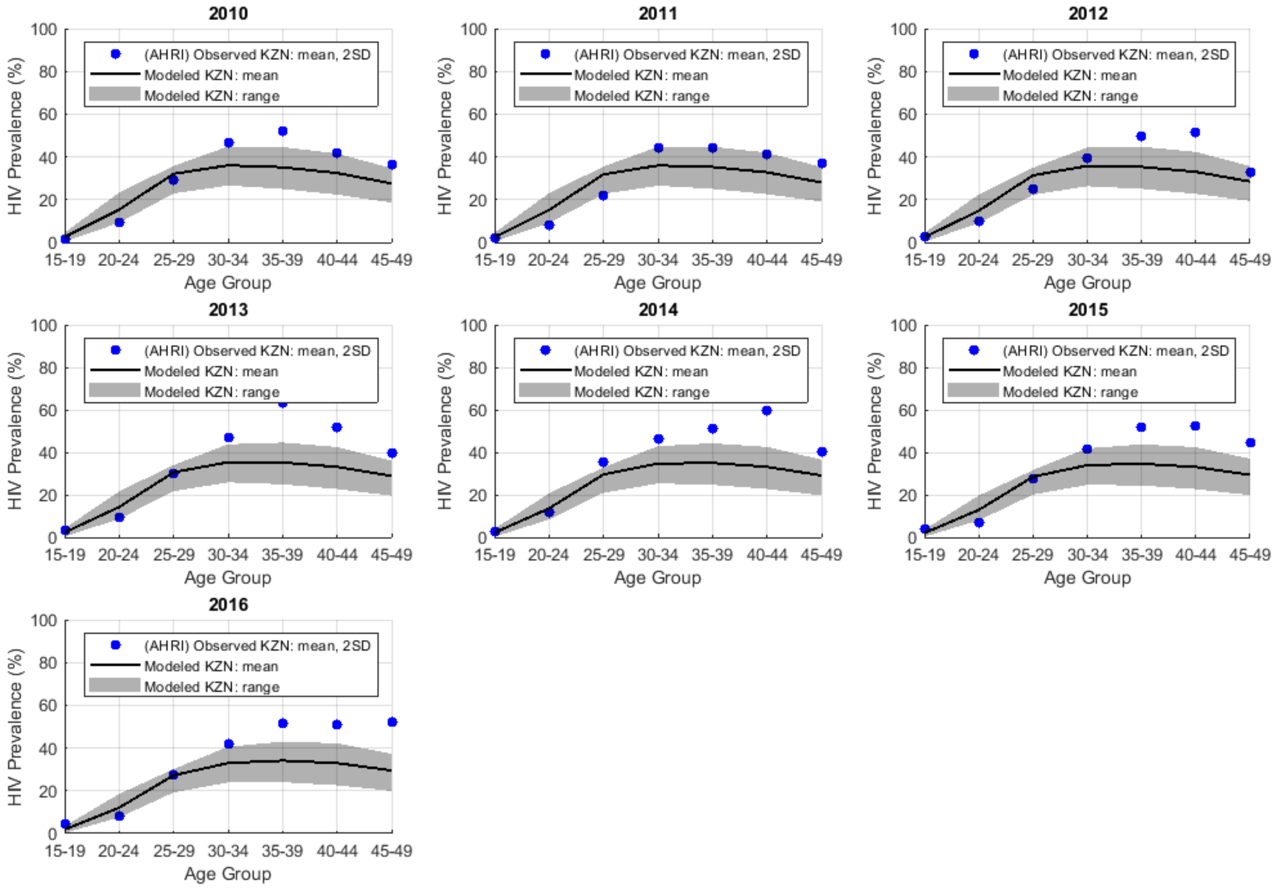

Figure K. Model validation to observed HIV prevalence data in men by age over time. Shaded regions represent the range of estimates using the 25 best-fitting model parameter sets.

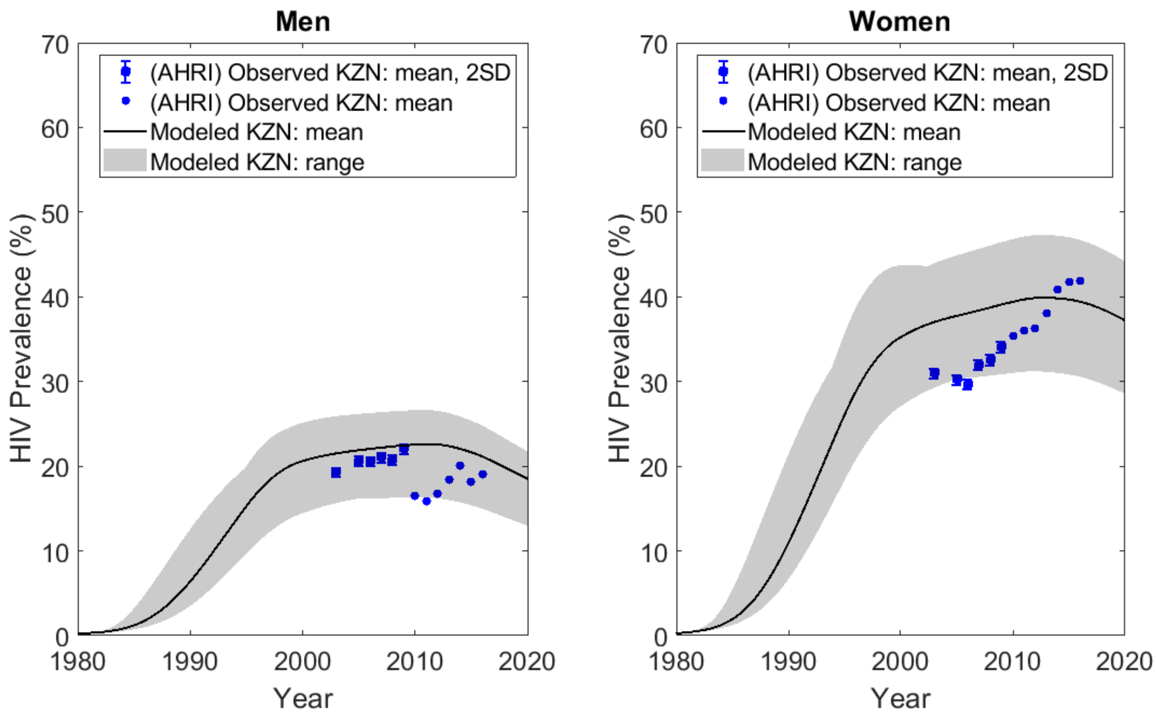

Figure L. Model validation to observed HIV prevalence data by gender over time. Blue error bars from 2003-2009 recalculate HIV prevalence for ages 15-49 combined from the calibration dataset, while blue data from 2010-2016 represents later timepoints used only for validation. Shaded regions represent the range of estimates using the 25 best-fitting model parameter sets.

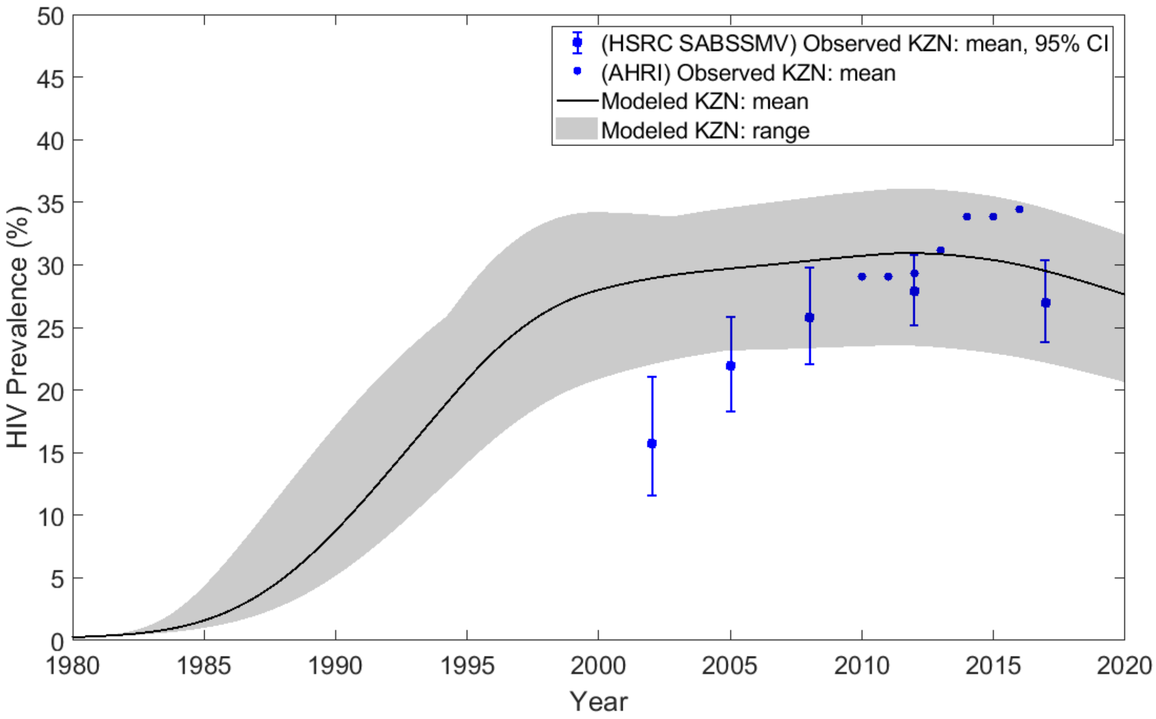

Figure M. Model validation to observed HIV prevalence data among ages 15-49 over time. Shaded region represents the range of estimates using the 25 best-fitting model parameter sets.

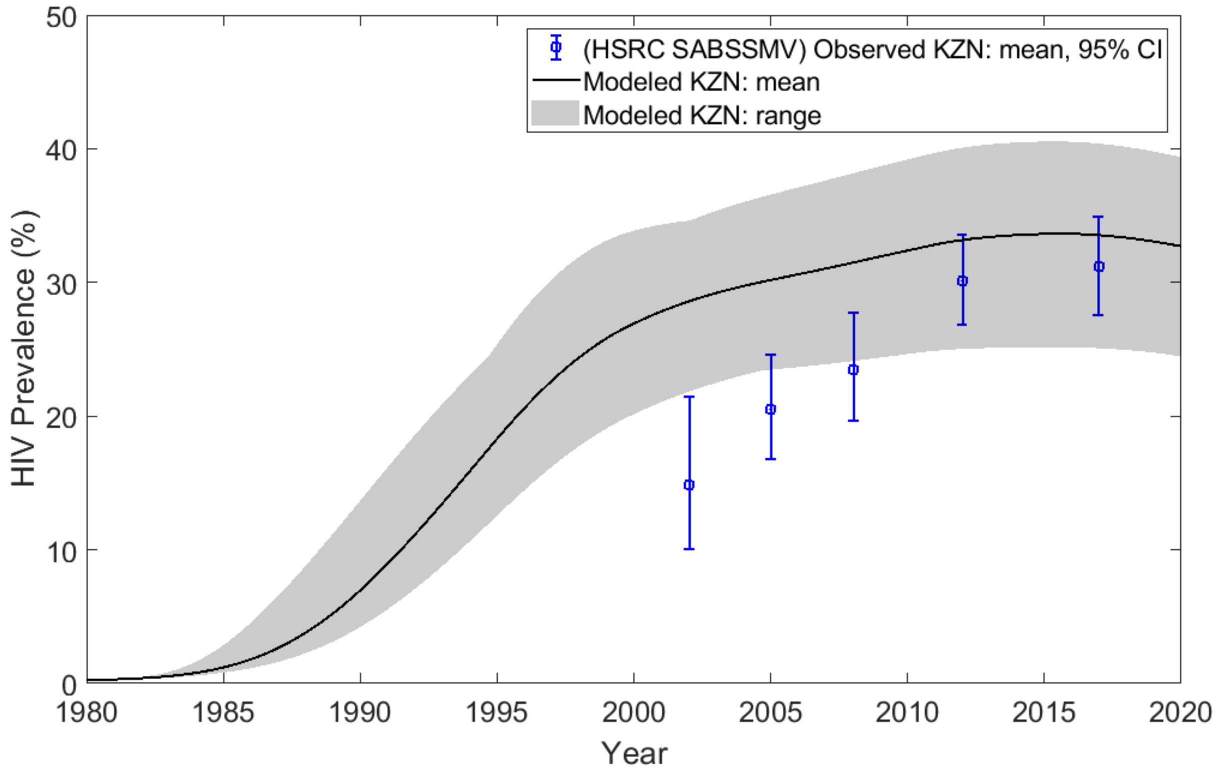

Figure N. Model validation to observed HIV prevalence data among ages 25+ over time. Shaded region represents the range of estimates using the 25 best-fitting model parameter sets.

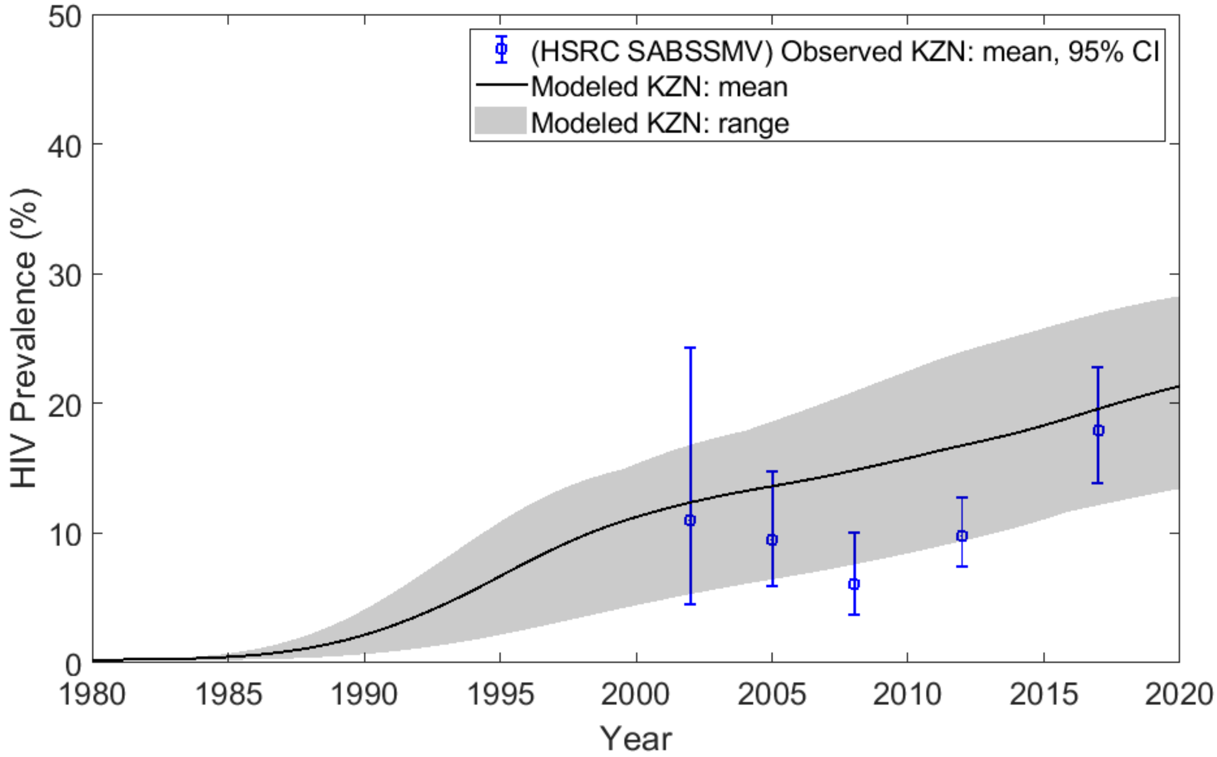

Figure O. Model validation to observed HIV prevalence data among ages 50+ over time. Shaded region represents the range of estimates using the 25 best-fitting model parameter sets.

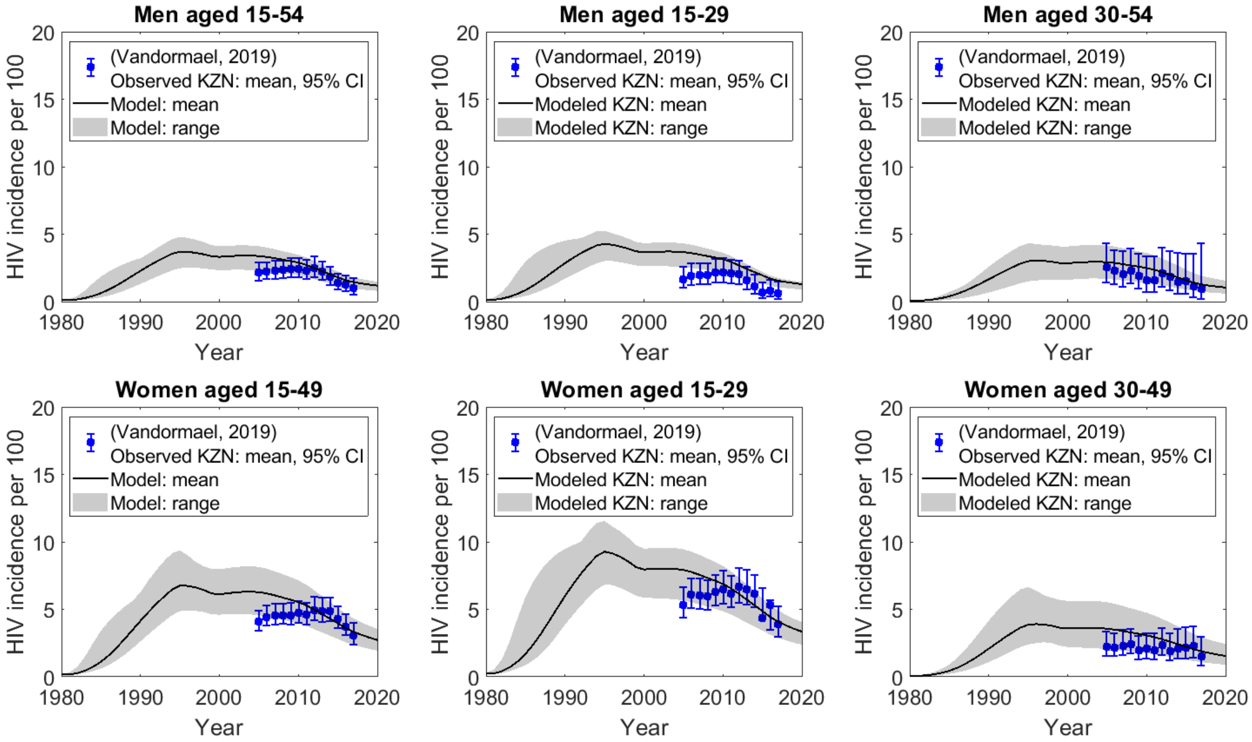

Figure P. Model validation to observed HIV incidence data by gender and age over time. Shaded regions represent the range of estimates using the 25 best-fitting model parameter sets.

(IV.) Model differential equations

We use a system of differential equations to estimate changes in population and infection dynamics over each time step. We split the full system of equations into topic-based modules and solve each iteratively using a 4th-order Runge-Kutta numerical solver in MATLAB. The order is largely historical and based on previously published work(3). The modules are:

1. HPV natural history
    a. Progression and clearance of HPV
    b. Progression and regression of precancerous lesions
    c. Development and progression of cervical cancer
    d. Cervical cancer- associated mortality
2. Cervical cancer screening and treatment
    a. Screening
    b. Treatment
3. HPV and HIV transmission
    a. Heterosexual mixing by gender, age, and risk group
    b. Partnership adjustment
    c. HPV infection by type
    d. HIV infection
4. HIV natural history and treatment
    a. CD4 progression
    b. Viral load progression
    c. ART initiation, discontinuation, and scale-up by CD4 count
    d. HIV-associated mortality
5. Demography
    a. Births
    b. Mother-to-child HIV transmission
    c. Aging and risk-group redistribution
    d. Natural deaths
6. Voluntary male medical circumcision
7. HPV vaccination
    a. School-based regimen
    b. Catch-up regimen

Ideally, each module would be completely independent from the others. This may not be the case here, but a comparative analysis revealed no significant difference in the results of split or combined approaches. We therefore chose the split approach for efficiency as it allows us to use a larger time step.

Throughout each simulation, we track population demographics and the number of persons with infection, with progressed disease, or with preventative or therapeutic treatment. We describe these states $X_{g,a,r}^{d,v,h,s,x,p}(t)$ with the following indices (using 1-based indexing):

Index	Description	Values
d	HIV disease state, CD4 count, circumcision status, and ART status	1. HIV-negative, uncircumcised<br>2. HIV-negative, circumcised<br>3. HIV-positive, acute infection<br>4. HIV-positive, CD4 > 500 cells/µL<br>5. HIV-positive, CD4 350-500 cells/µL<br>6. HIV-positive, CD4 200-350 cells/µL<br>7. HIV-positive, CD4 ≤ 200 cells/µL<br>8. HIV-positive, on ART
v	HIV viral load	1. If ($2 < d < 8$), Acute infection; if (d = 1,2), HIV-negative: VL = 0.0<br>2. Asymptomatic: VL = 3.0-4.5 $\log_{10}$<br>3. Pre-AIDS symptomatic: VL = 4.0-5.5 $\log_{10}$<br>4. AIDS: VL = 5.5-7.0 $\log_{10}$<br>5. Late-stage<br>6. On ART and virally suppressed: VL = 0.0
h	Vaccine-type HPV precancer or disease state	1. Susceptible<br>2. Infected<br>3. CIN1<br>4. CIN2<br>5. CIN3<br>6. Cervical cancer or hysterectomy<br>7. Immune
s	Non-vaccine type HPV precancer or disease state	1. Susceptible<br>2. Infected<br>3. CIN1<br>4. CIN2<br>5. CIN3<br>6. Cervical cancer or hysterectomy<br>7. Immune
x	Cervical cancer or hysterectomy status	1. If (h = 6 or s = 6), Cervical cancer, local; else, no cancer or hysterectomy<br>2. Cervical cancer, regional<br>3. Cervical cancer, distant<br>4. Hysterectomy
p	Vaccination and screening history	1. Non-vaccinated, non-screened<br>2. Vaccinated<br>3. Screened<br>4. Vaccinated and screened
g	Gender	1. Men<br>2. Women

a	Age	1. 0-4<br>2. 5-9<br>3. 10-14<br>4. 15-19<br>5. 20-24<br>6. 25-29<br>7. 30-34<br>8. 35-39<br>9. 40-44<br>10. 45-49<br>11. 50-54<br>12. 55-59<br>13. 60-64<br>14. 65-69<br>15. 70-74<br>16. 75-79
r	Risk	1. Low risk<br>2. Moderate risk<br>3. High risk

(IV.a.) Demography

Equation variables	
γ_a^d(t)	The annual fertility rate for women by age *a* and HIV disease stage *d*. Women ages 15-49 bear children.
$\eta(t)$	The proportion of births from women with HIV that result in vertical transmission.
$b\_s(t)$	Number of births by HIV-negative women and women on ART.
$b\_i(t)$	Number of births by women living with HIV.
$b_{g,a,r}^{d,1,h,s,x,p}(t)$	Number of infant births of HIV disease stage *d* and gender *g.* We assume an equal gender ratio at birth of 1:1, that all newborns are born as low risk, no vertical transmission of HPV, and that if HIV is vertically transmitted, that infected newborns are born into the acute stage of HIV.
$\phi_{g,a,r}$	Distribution of sexual risk *r* by gender *g* and age *a.* (Currently, the risk distribution derived from male partner data is used for both men and women for simplicity.)
$\mu\_bkrd_{g,a}(t)$	Annual background mortality rate by gender *g* and age *a.*

Fertility

The number of births by HIV status of the mother are calculated as:

HIV-negative women and women on ART

$$b\_s(t) = \sum_{h=1}^{7}\sum_{s=1}^{7}\sum_{x=1}^{3}\sum_{p=1}^{4}\sum_{a=4}^{10}\sum_{r=1}^{3}\left[\gamma_a^1(t)\cdot X_{g,a,r}^{1,1,h,s,x,p}(t) + \gamma_a^8(t)\cdot X_{g,a,r}^{8,6,h,s,x,p}(t)\right]$$

Women living with HIV

$$b\_i(t) = \sum_{d=3}^{7}\sum_{v=1}^{5}\sum_{h=1}^{7}\sum_{s=1}^{7}\sum_{x=1}^{3}\sum_{p=1}^{4}\sum_{a=4}^{10}\sum_{r=1}^{3}\left[\gamma_a^d(t)\cdot X_{g,a,r}^{d,v,h,s,x,p}(t)\right]$$

We then compute the number of births by gender and HIV status of the infant as:

HIV-negative, uncircumcised births

For h = s = x = p = a = r = 1,

$$b_{g,a,r}^{1,1,h,s,x,p}(t) = 0.5\left(b\_s(t) + \left(1-\eta(t)\right)b\_i(t)\right)$$

else,

$$b_{g,a,r}^{1,1,h,s,x,p}(t) = 0$$

HIV-positive births

For h = s = x = p = a = r = 1,

$$b_{g,a,r}^{3,1,h,s,x,p}(t) = 0.5\,\eta(t) \cdot b\_i(t)$$

else,

$$b_{g,a,r}^{3,1,h,s,x,p}(t) = 0$$

Aging

To age the population, one-fifth of each compartment moves to the next age group while maintaining the same gender, disease state, and sexual risk distribution $\phi_{g,a,r}$:

$$\frac{dX_{g,1,r}^{d,v,h,s,x,p}(t)}{dt} = -\frac{1}{5}\sum_{r=1}^{3} X_{g,1,r}^{d,v,h,s,x,p}(t) \cdot \phi_{g,a,r} \qquad (for\ a = 1)$$

$$\frac{dX_{g,a,r}^{d,v,h,s,x,p}(t)}{dt} = -\frac{1}{5}\sum_{r=1}^{3} X_{g,a,r}^{d,v,h,s,x,p}(t) \cdot \phi_{g,a,r} + \frac{1}{5}\sum_{r=1}^{3} X_{g,a-1,r}^{d,v,h,s,x,p}(t) \cdot \phi_{g,a-1,r} \qquad (for\ a \neq 1)$$

Upon aging to the next five-year group, individuals are re-distributed into the closest unfilled risk group to match observed data on the age distribution of low, moderate, and high-risk individuals.

Mortality

We compute the number of deaths due to background mortality as:

$$\frac{dX_{g,a,r}^{d,v,h,s,x,p}(t)}{dt} = -\mu\_bkrd_{g,a}(t) \cdot X_{g,a,r}^{d,v,h,s,x,p}(t)$$

(IV.b.) Sexual Behavior

Mixing matrix

Equation variables	
$c_{g,a,r}$	Number of partners a person has per year of gender *g,* age *a,* and sexual-risk group *r* (ie. the partner exchange rate, or contact rate).
ϵ_a	Mixing parameter by age *a.*<br>We assume a mixing pattern that is partially random and partially off-diagonal (0 < ϵ_a < 1), where ($\epsilon_a = 0$) indicates completely off-diagonal mixing, and ($\epsilon_a = 1$) indicates completely random mixing.
ϵ_r	Mixing parameter by sexual-risk group *r.*<br>We assume a mixing pattern that is partially random and partially on-diagonal (0 < ϵ_r < 1), where ($\epsilon_r = 0$) indicates completely on-diagonal mixing, and ($\epsilon_r = 1$) indicates completely random mixing.
$\delta_{g,a,a'}$	Mixing pattern by age.<br>In completely non-random mixing by age, women are most likely to form partnerships with men of the next oldest age group. We represent this pattern using on off-diagonal matrix.<br><br>For men (*g* = 1) of age *a* mixing with women of age *a'*:<br>= 0.3 if $(a = a')$<br>= 0.7 if $(a = a' + 1)$<br>except for the following (correct for no sexual activity before age group 3):<br>= 0.0 if $(a = a' = 1)$<br>= 0.0 if $(a = 2)$ and (*a'* = 1)<br>= 0.0 if $(a = 2)$ and (*a'* = 2)<br>= 0.0 if $(a = 3)$ and (*a'* = 2)<br><br>For women (*g* = 2) of age *a* mixing with men of age *a'*:<br>= 0.3 if $(a = a')$<br>= 0.7 if $(a = a' - 1)$<br>except for the following (correct for no sexual activity before age group 3):<br>= 0.0 if $(a = a' = 1)$<br>= 0.0 if $(a = 1)$ and (*a'* = 2)<br>= 0.0 if $(a = a' = 2)$<br>= 0.0 if $(a = 2)$ and (*a'* = 3)
$\delta_{r,r'}$	Mixing pattern by risk.<br>Completely non-random mixing by risk confines sexual encounters to individuals within the same risk group. We represent this pattern using an identity matrix.<br>= 1.0 if $(r = r')$<br>= 0.0 if $(r \neq r')$

For a person of gender *g,* age *a,* and sexual-risk group *r,* we use the mixing matrix $\rho_{g,a,a',r,r'}(t)$ to describe the proportion of sexual partners that come from age group *a'* and sexual-risk group *r'*. We assume that mixing is partially random and partially designated by a mixing pattern $\delta_{g,a,a'}$ or $\delta_{r,r'}$. The overall mixing matrix is therefore a weighted average of random mixing proportional to the number of available

partnerships of each group, and mixing among groups with similar characteristics. Although an off-diagonal mixing pattern results in the first and last ages groups (ages 10-14 and 75-79) having fewer than 100% of their partnerships, these age groups have relatively few partnerships and contribute marginally to overall infection transmission.

$$\rho_{g,a,a',r,r'}(t) = \left(\epsilon_a \cdot \frac{\sum_{r'=1}^{3} \left(c_{g',a',r'} \cdot \sum_{d'=1}^{8} \sum_{v'=1}^{6} \sum_{h'=1}^{7} \sum_{s'=1}^{7} \sum_{x'=1}^{4} \sum_{p'=1}^{4} X_{g',a',r'}^{d',v',h',s',x',p'}(t) \right)}{\sum_{a'=1}^{16} \sum_{r'=1}^{3} \left(c_{g',a',r'} \cdot \sum_{d'=1}^{8} \sum_{v'=1}^{6} \sum_{h'=1}^{7} \sum_{s'=1}^{7} \sum_{x'=1}^{4} \sum_{p'=1}^{4} X_{g',a',r'}^{d',v',h',s',x',p'}(t) \right)} + (1-\epsilon_a)\delta_{g,a,a'} \right) \cdot \left(\epsilon_r \cdot \frac{\left(c_{g',a',r'} \cdot \sum_{d'=1}^{8} \sum_{v'=1}^{6} \sum_{h'=1}^{7} \sum_{s'=1}^{7} \sum_{x'=1}^{4} \sum_{p'=1}^{4} X_{g',a',r'}^{d',v',h',s',x',p'}(t) \right)}{\sum_{r'=1}^{3} \left(c_{g',a',r'} \cdot \sum_{d'=1}^{8} \sum_{v'=1}^{6} \sum_{h'=1}^{7} \sum_{s'=1}^{7} \sum_{x'=1}^{4} \sum_{p'=1}^{4} X_{g',a',r'}^{d',v',h',s',x',p'}(t) \right)} + (1-\epsilon_r)\delta_{g,r,r'} \right)$$

Rate of partner change

Equation variables	
$c_{g,a,r}$	Number of partners a person has per year of gender *g,* age *a,* and sexual-risk group *r* (ie. the partner exchange rate, or contact rate). We assume zero partnerships for individuals below the age of sexual debut (age 10).
θ	Gender influence on contact rate adjustment. We assume an adjusted contact rate equally driven by rates reported by men and women (θ = 0.5), where (θ = 0) when completely female-driven, and (θ = 1) when completely male-driven.
$\rho_{g,a,a',r,r'}(t)$	Mixing matrix for a person of gender *g,* age *a,* and sexual-risk group *r* that describes the proportion of sexual partners that come from age group *a′* and sexual-risk group *r′.* We assume a solely heterosexual population and therefore that all contacts are with the opposite gender.

Bias in observed data leads to contact rates $c_{g,a,r}$ that, when assuming solely heterosexual contact, are inconsistent between men and women. We account for this variability by using an adjusted contact rate $c^{*}_{g,a,a',r,r'}(t)$ that ensures that the number of partnerships of men of age *a* and risk group *r* with women of age *a′* and risk group *r′* equals the number of partnerships of women of age *a* and risk group *r* with men of age *a′* and risk group *r′*.

We first calculate the discrepancy between reported contacts among men and women as:

$$B_{a,a',r,r'}(t) = \frac{c_{1,a,r} \cdot \rho_{1,a,a',r,r'}(t) \cdot \sum_{d'=1}^{8}\sum_{v'=1}^{6}\sum_{h'=1}^{7}\sum_{s'=1}^{7}\sum_{x'=1}^{4}\sum_{p'=1}^{4} X_{1,a',r'}^{d',v',h',s',x',p'}(t)}{c_{2,a,r} \cdot \rho_{2,a,a',r,r'}(t) \cdot \sum_{d=1}^{8}\sum_{v=1}^{6}\sum_{h=1}^{7}\sum_{s=1}^{7}\sum_{x=1}^{4}\sum_{p=1}^{4} X_{2,a,r}^{d,v,h,s,x,p}(t)}$$

We then compute the adjusted contact rate for women as:

$$c^{*}_{2,a,a',r,r'}(t) = c_{2,a,r} \cdot \rho_{2,a,a',r,r'}(t) \cdot B_{a,a',r,r'}(t)^{\theta} \cdot \left(\frac{\sum_{d'=1}^{8}\sum_{v'=1}^{6}\sum_{h'=1}^{7}\sum_{s'=1}^{7}\sum_{x'=1}^{4}\sum_{p'=1}^{4} X_{1,a',r'}^{d',v',h',s',x',p'}(t)}{\sum_{d=1}^{8}\sum_{v=1}^{6}\sum_{h=1}^{7}\sum_{s=1}^{7}\sum_{x=1}^{4}\sum_{p=1}^{4} X_{2,a,r}^{d,v,h,s,x,p}(t)}\right)^{-(1-\theta)}$$

and for men, an adjusted contact rate of:

$$c^{*}_{1,a,a',r,r'}(t) = c_{1,a,r} \cdot \rho_{1,a,a',r,r'}(t) \cdot B_{a,a',r,r'}(t)^{-(1-\theta)} \cdot \left(\frac{\sum_{d'=1}^{8}\sum_{v'=1}^{6}\sum_{h'=1}^{7}\sum_{s'=1}^{7}\sum_{x'=1}^{4}\sum_{p'=1}^{4} X_{1,a',r'}^{d',v',h',s',x',p'}(t)}{\sum_{d=1}^{8}\sum_{v=1}^{6}\sum_{h=1}^{7}\sum_{s=1}^{7}\sum_{x=1}^{4}\sum_{p=1}^{4} X_{2,a,r}^{d,v,h,s,x,p}(t)}\right)^{\theta}$$

(IV.c.) Transmission Probabilities

Per-partnership probability of transmission

Equation variables	
$A_{g,a,r}$	Number of acts a person has per partnership of gender *g,* age *a,* and sexual-risk group *r*. We assume zero acts for individuals below the age of sexual debut (age 10).
$\chi\_HIV_g^{v',x'}$	Per-act probability of HIV transmission to a person of gender *g* based on the viral load *v′* of the partner with HIV. We assume the probability of female-to-male HIV transmission is equal to the probability of male-to-female transmission across all viral load stages ($\chi\_HIV_1^{v'} = \chi\_HIV_2^{v'}$). We reduce HIV per-act transmission as a proxy for decreased sexual activity during late-stage HIV (*v′* = 5), regional or distant cervical cancer (*x′* = 2 or *x′* = 3), or hysterectomy (*x′* = 4).

The per-partnership probability of HIV transmission $\beta\_HIV_{g,a,r}^{v',x'}$ is the cumulative risk of acquiring HIV from all sexual acts with a partner. This quantity depends on the per-act probability of HIV transmission and the number of acts per partnership.

We calculate the per-partnership probability of HIV transmission to a male partner:

$$\beta\_HIV_{1,a,r}^{v',x'} = 1 - (1 - \chi\_HIV_1^{v',x'})^{A_{1,a,r}}$$

Similarly, the per-partnership probability of HIV transmission to a female partner:

$$\beta\_HIV_{2,a,r}^{v',x'} = 1 - (1 - \chi\_HIV_2^{v',x'})^{A_{2,a,r}}$$

Force of infection

Equation variables	
$c^*_{g,a,a',r,r'}(t)$	Adjusted yearly contact rate for persons of gender *g,* age *a,* and risk group *r,* with persons of the opposite gender, age *a',* and risk group *r'.*
$\beta\_HIV^{v',x'}_{g,a,r}$	Annual per-partnership probability of HIV transmission from a person with HIV with viral load *v'* and cervical cancer stage *x'* to a HIV-susceptible partner with gender *g,* age *a,* and risk group *r.*

The force of infection represents the cumulative risk of acquiring HIV from all possible partners, and depends on the adjusted contact rate, the per-partnership probability of transmission, and the proportion of sexually active persons who are HIV-infected.

The force of infection $\lambda\_HIV_{g,a,r}(t)$ determines HIV disease transmission:

$$\lambda\_HIV_{g,a,r}(t) = \sum_{a'=1}^{16}\sum_{r'=1}^{3}\left(c^*_{g,a,a',r,r'}(t) \cdot \frac{-\sum_{v'=1}^{6}\sum_{x'=1}^{4}\ln\left(1-\beta\_HIV^{v',x'}_{g,a,r}\right)\cdot\sum_{d'=3}^{8}\sum_{h'=1}^{7}\sum_{s'=1}^{7}\sum_{p'=1}^{4}X^{d',v',h',s',x',p'}_{g',a',r'}(t)}{\sum_{d'=1}^{8}\sum_{v'=1}^{6}\sum_{h'=1}^{7}\sum_{s'=1}^{7}\sum_{x'=1}^{4}\sum_{p'=1}^{4}X^{d',v',h',s',x',p'}_{g',a',r'}(t)}\right)$$

(IV.d.) Natural History and Interventions

HIV

Equation variables	
$\mu\_HIV_{g,a}^{d}$	Annual HIV-associated mortality rate by gender *g*, age *a*, and HIV disease stage *d* for (3 ≤ *d* ≤ 8).
$\lambda\_HIV_{g,a,r}(t)$	Force of HIV infection for HIV-negative persons by gender *g,* age *a,* and risk *r*.
ρ_HIV_g	Reduction in HIV acquisition due to circumcision by gender.<br>Only men receive circumcision ($\rho\_HIV_2 = 1$).
ψ_HIV_g	Reduction in HIV acquisition due to population-level condom use by gender.
ω^d	The rate of progressing from HIV stage *d* to stage *d + 1,* for (3 ≤ *d* ≤ 7).
l^d	The rate of progressing from viral load stage *v* to *v + 1,* for (1 ≤ *v* ≤ 5).
$P_{g,a}(t)$	The proportion of HIV-negative persons of gender *g* and age *a* that are circumcised.<br>Only men receive circumcision ($P_{2,a}(t)$ = 0).
$A_{g,a}^{d}(t)$	The proportion of persons living with HIV of disease stage *d,* gender *g,* and age *a* that initiate ART.
$\sigma_{g,a,r}^{d,v}(t)$	The proportion of persons who discontinue ART based on the recent distribution of persons initiating ART by gender *g*, age *a,* risk *r,* disease *d,* and viral *v*.

We calculate changes in HIV status and HIV stage defined by CD4 count, viral load, and treatment status. The population of persons without HIV can acquire HIV after sexual debut with a force of infection reduced by circumcision in men and condom use by either gender. We only track circumcision among men without HIV. Individuals with HIV infection experience HIV-associated mortality, CD4 and viral load stage progression, and ART initiation and discontinuation. CD4 and viral load stage are not tracked among persons on treatment.

HIV-negative, uncircumcised

$$\frac{dX_{g,a,r}^{1,1,h,s,x,p}(t)}{dt} = -\left(\psi\_HIV_g \cdot \lambda\_HIV_{g,a,r}(t) \, + \, P_{g,a}(t)\right) X_{g,a,r}^{1,1,h,s,x,p}(t)$$

HIV-negative, circumcised

$$\frac{dX_{g,a,r}^{2,1,h,s,x,p}(t)}{dt} = P_{g,a}(t) \cdot X_{g,a,r}^{1,1,h,s,x,p}(t) - \left(\psi\_HIV_g \cdot \rho\_HIV_g \cdot \lambda\_HIV_{g,a,r}(t)\right) X_{g,a,r}^{2,1,h,s,x,p}(t)$$

HIV-positive, acute infection

$$\frac{dX_{g,a,r}^{3,1,h,s,x,p}(t)}{dt} = \psi\_HIV_g \cdot \lambda\_HIV_{g,a,r}(t) \cdot X_{g,a,r}^{1,1,h,s,x,p}(t)$$

$$+ \psi\_HIV_g \cdot \rho\_HIV_g \cdot \lambda\_HIV_{g,a,r}(t) \cdot X_{g,a,r}^{2,1,h,s,x,p}(t) + \sigma_{g,a,r}^{3,1}(t) \cdot X_{g,a,r}^{8,6,h,s,x,p}(t)$$

$$- \left(\mu\_HIV_{g,a}^{3} + \omega^3 + A_{g,a}^{3}(t)\right) X_{g,a,r}^{3,1,h,s,x,p}(t)$$

HIV-positive, CD4 > 500 cells/µL

$$\frac{dX_{g,a,r}^{4,v,h,s,x,p}(t)}{dt} = \omega^3 \cdot X_{g,a,r}^{3,v,h,s,x,p}(t) + l^{v-1} \cdot X_{g,a,r}^{4,v-1,h,s,x,p}(t) + \sigma_{g,a,r}^{4,v}(t) \cdot X_{g,a,r}^{8,6,h,s,x,p}(t)$$

$$- \left(\mu\_HIV_{g,a}^{4} + \omega^4 + l^v + A_{g,a}^{4}(t)\right) X_{g,a,r}^{4,v,h,s,x,p}(t)$$

HIV-positive, CD4 350-500 cells/µL

$$\frac{dX_{g,a,r}^{5,v,h,s,x,p}(t)}{dt} = \omega^4 \cdot X_{g,a,r}^{4,v,h,s,x,p}(t) + l^{v-1} \cdot X_{g,a,r}^{5,v-1,h,s,x,p}(t) + \sigma_{g,a,r}^{5,v}(t) \cdot X_{g,a,r}^{8,6,h,s,x,p}(t)$$

$$- \left(\mu\_HIV_{g,a}^{5} + \omega^5 + l^v + A_{g,a}^{5}(t)\right) X_{g,a,r}^{5,v,h,s,x,p}(t)$$

HIV-positive, CD4 200-350 cells/µL

$$\frac{dX_{g,a,r}^{6,v,h,s,x,p}(t)}{dt} = \omega^5 \cdot X_{g,a,r}^{5,v,h,s,x,p}(t) + l^{v-1} \cdot X_{g,a,r}^{6,v-1,h,s,x,p}(t) + \sigma_{g,a,r}^{6,v}(t) \cdot X_{g,a,r}^{8,6,h,s,x,p}(t)$$

$$- \left(\mu\_HIV_{g,a}^{6} + \omega^6 + l^v + A_{g,a}^{6}(t)\right) X_{g,a,r}^{6,v,h,s,x,p}(t)$$

HIV-positive, CD4 ≤ 200 cells/µL

$$\frac{dX_{g,a,r}^{7,v,h,s,x,p}(t)}{dt} = \omega^6 \cdot X_{g,a,r}^{6,v,h,s,x,p}(t) + l^{v-1} \cdot X_{g,a,r}^{7,v-1,h,s,x,p}(t) + \sigma_{g,a,r}^{7,v}(t) \cdot X_{g,a,r}^{8,6,h,s,x,p}(t)$$

$$- \left(\mu\_HIV_{g,a}^{7} + \omega^7 + l^v + A_{g,a}^{7}(t)\right) X_{g,a,r}^{7,v,h,s,x,p}(t)$$

HIV-positive, on ART

$$\frac{dX_{g,a,r}^{8,6,h,s,x,p}(t)}{dt} = \sum_{d=3}^{7} \sum_{v=1}^{5} \left(A_{g,a}^{d}(t) \cdot X_{g,a,r}^{d,v,h,s,x,p}(t) - \sigma_{g,a,r}^{d,v} \cdot X_{g,a,r}^{8,6,h,s,x,p}(t) \right)$$

(V.) Scenario descriptions

Table Q. Percent of persons with HIV who know their status under different HIV testing interventions.

Testing Intervention	Gender	Percent who know their status of all persons with HIV	References
**Baseline HIV testing**	**Women**	88.9%	Baseline HIV testing coverage from SABSSMV, Table 3.33 (2).
	**Men**	78%	Baseline HIV testing coverage from SABSSMV, Table 3.33 (2).
**Baseline HIV testing + Home HIV testing and counseling (HTC) campaigns 1x/5 years**	**Women**	88.9% + 75% x (100% - 88.9%) HTC 1x/5 years = 93%*	Baseline HIV testing coverage from SABSSMV, Table 3.33 (2), with additional home HTC campaigns for persons with HIV not reached by baseline testing (56).
	**Men**	78% + 75% x (100% - 78%) HTC 1x/5 years = 92%*	Baseline HIV testing coverage from SABSSMV, Table 3.33 (2), with additional home HTC campaigns for persons with HIV not reached by baseline testing (56).

*Percentages are averages over the 2020-2060 timeframe; the exact percent of persons with HIV who know their status fluctuates over time.

Table R. Percent of persons with HIV who know their status who are on ART and achieve viral suppression under different HIV treatment interventions. We assume that the increase in viral suppression with community ART is achieved solely by an increase in the percent of persons with HIV who know their status and are on ART achieving viral suppression; the percent of persons with HIV who know their status and are on community ART is the same as those diagnosed that are on clinic ART.

Treatment intervention	Gender	1<br>Percent on ART of persons with HIV who know their status	2<br>Percent who achieve viral suppression of persons with HIV who know their status and are on ART	3<br>Percent who achieve viral suppression of all persons with HIV who know their status (% on ART x % virally suppressed)	References
**Clinic ART**	**Women**	73.85%	94.79%	70%	Column 3 values represent DO ART Study results for the KwaZulu-Natal setting (43). Column 2 values derived from SABSSMV, Table 3.33 (2). Column 1 back-calculated from columns 2 and 3.
	**Men**	72.64%	70.21%	51%	
**Community ART for persons with HIV not reached in clinic**	**Women**	73.85%	98.85%	73%	Column 3 values represent DO ART Study results for the KwaZulu-Natal setting (43). Column 1 values set equal to Clinic ART, and Column 2 values back-calculated from columns 1 and 3.
	**Men**	72.64%	99.12%	72%	

Table S. Modeled HIV testing and treatment intervention scenarios, including a third scenario with Home Testing + Clinic ART.

#	Scenario Description	Gender	Distribution of persons with HIV who are on ART		Percent of persons with HIV virally suppressed (% diagnosed x % on ART & suppressed)	Costs
			Clinic ART	Community ART		
1	Standard of Care<br>Baseline HIV testing +<br>Clinic ART only	Women	100%	0%	88.9% x 70% = 62.23%	• Clinic ART<br>• Hospitalization
		Men	100%	0%	78% x 51% = 39.78%	
2	Home Testing + Community ART<br>Baseline HIV testing +<br>Home HTC campaigns 1x/5 years +<br>Clinic ART +<br>Community ART for persons with HIV not reached in clinic	Women	91.83%*	8.17%*	93%* x 73% = 68%*	• 1x/5 years home HTC campaigns<br>• Clinic ART<br>• Community ART<br>• Hospitalization
		Men	68.17%*	31.83%*	92%* x 72% = 66%*	
3	Home Testing + Clinic ART<br>Baseline HIV testing +<br>Home HTC campaigns 1x/5 years +<br>Clinic ART only	Women	100%	0%	93%* x 70% = 65%*	• 1x/5 years home HTC campaigns<br>• Clinic ART<br>• Hospitalization
		Men	100%	0%	92%* x 51% = 47%*	

*For Scenarios 2 and 3, percentages are averages over the 2020-2060 timeframe; because the exact percent of persons with HIV who know their status fluctuates over time, the distribution of people on ART and population-level viral suppression fluctuate accordingly.

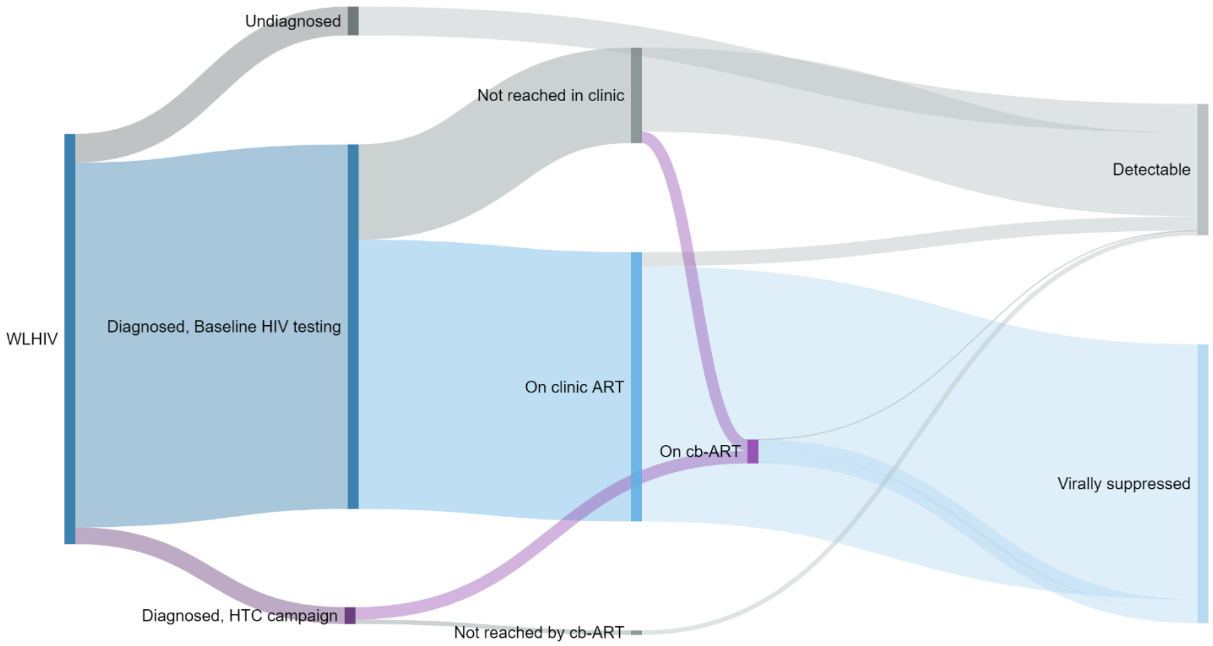

Figure Q. Distribution of women with HIV who are diagnosed, on ART, and virally suppressed with different HIV testing and treatment interventions to achieve observed DO ART viral suppression rates at the population-level. Community ART is abbreviated as cb-ART and home HIV testing and counseling campaigns are abbreviated as "HTC campaigns."

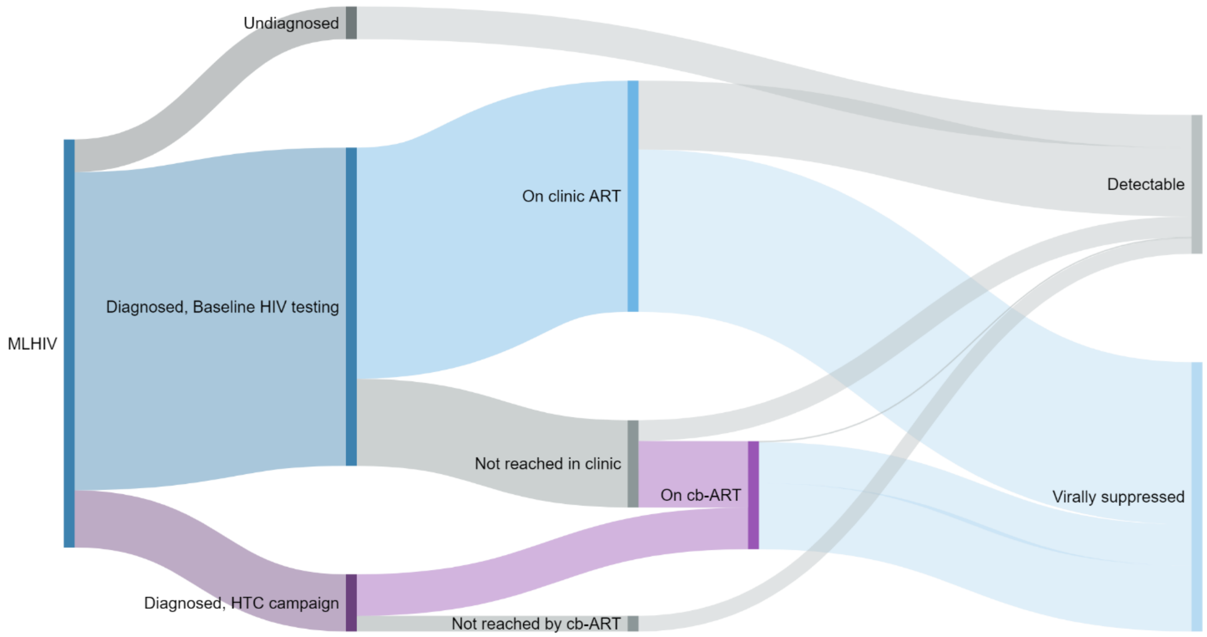

Figure R. Distribution of men with HIV who are diagnosed, on ART, and virally suppressed with different HIV testing and treatment interventions to achieve observed DO ART viral suppression rates at the population-level. Community ART is abbreviated as cb-ART and home HIV testing and counseling campaigns are abbreviated as "HTC campaigns."

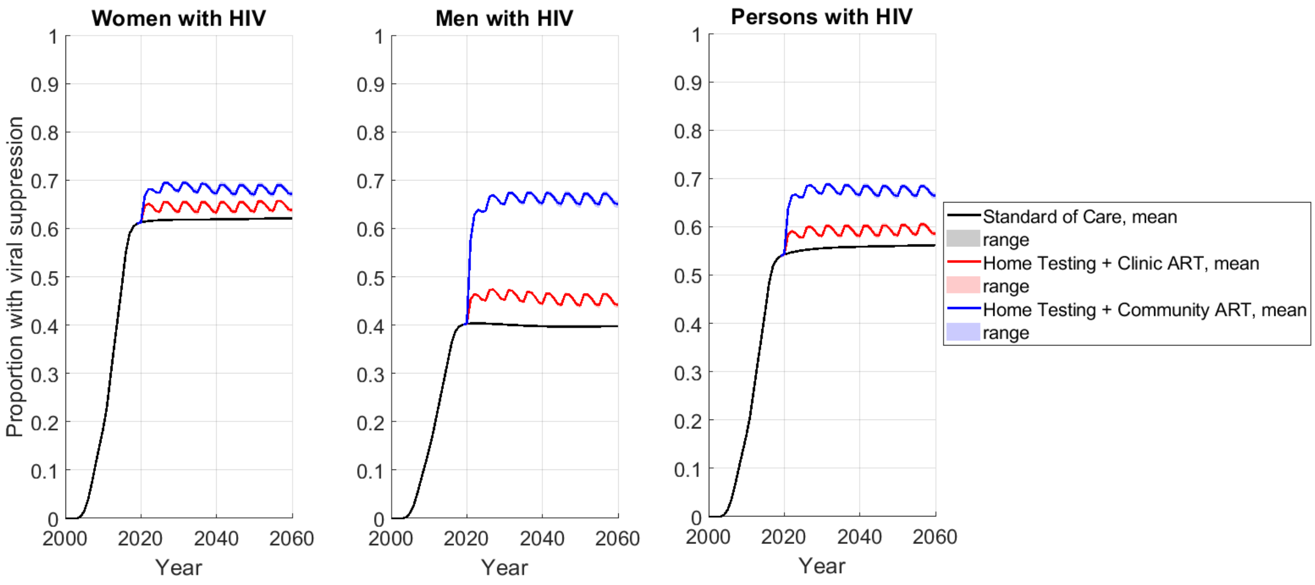

Figure S. Proportion of persons with HIV virally suppressed over time by gender under different scenarios.

Calculating the proportions of population groups who receive community ART in order to achieve the viral suppression rates observed in the DO ART Community Group at the population-level in Scenario 2

**Equation variables**	
$pDiag_g^{base}$	Proportion of persons of gender *g* who know their status with baseline HIV testing under the standard of care
$pART_g^{clinic}$	Proportion of persons of gender *g* on clinic ART, of those with HIV who know their status
pVS_g^{clinic}	Proportion of persons of gender *g* with viral suppression, of those with HIV who know their status and are on clinic ART
$pDiag_g^{htc}$	Proportion of persons of gender *g* who know their status with baseline HIV testing + home HIV testing and counseling campaigns every five years
$pART_g^{cb}$	Proportion of persons of gender *g* on community ART, of those with HIV who know their status
pVS_g^{cb}	Proportion of persons of gender *g* with viral suppression, of those with HIV who know their status and are on community ART
$pSwitch_g^1$	Proportion of persons of gender *g* who switch to community ART and achieve viral suppression, of those who were diagnosed with baseline HIV testing but who were not reached by clinic ART
$pSwitch_g^2$	Proportion of persons of gender *g* who switch to community ART and achieve viral suppression, of those who were diagnosed at baseline and who received clinic ART but who were not virally suppressed
$pDiag\_ART\_VS_g^{clinic+cb}$	Proportion of persons of gender *g* who are diagnosed, on ART, and virally suppressed under the Home Testing + Community ART scenario
pCb_g	Proportion of persons on community ART of all those on ART in the Home Testing + Community ART scenario
$pClinic_g$	Proportion of persons on clinic ART of all those on ART in the Home Testing + Community ART scenario

In the Standard of Care scenario, the proportions of diagnosed persons living with HIV with viral suppression reflected observed viral suppression rates from the clinic ART group of the DO ART Study. In the Home Testing + Community ART scenario, we calibrated the proportions of men and women living with HIV but not reached by clinic-based care who initiated community ART in order to achieve the viral suppression results observed in the Community ART group of the DO ART Study. This was done using a tiered method outlined by Table T. First, we assumed that all persons newly diagnosed during home HIV testing campaigns were put on community ART (row 1 of Table T). Then, $pSwitch_g^1$ was calculated as the proportion of persons with HIV who were diagnosed with baseline HIV testing, but who were not reached by clinic ART, who were put on community ART (row 2 of Table T). The proportion $pSwitch_g^2$ was also built into the equation, but ultimately no persons with HIV who were diagnosed at baseline and who received clinic ART but who were not virally suppressed needed to be moved to community ART (row 3 of Table T). We solved for $pSwitch_g^1$ and $pSwitch_g^2$ using the following equation and the values in Tables S17 and S18:

$$\begin{aligned} pDiag\_ART\_VS_g^{clinic+cb} &= \left(pDiag_g^{base} \cdot pART_g^{clinic} \cdot pVS_g^{clinic}\right) + \left((pDiag_g^{htc} - pDiag_g^{base}) \cdot pART_g^{cb} \cdot pVS_g^{cb}\right) \\ &+ pSwitch_g^1 \cdot \left(pDiag_g^{base} \cdot \left(1 - pART_g^{clinic}\right) \cdot pVS_g^{cb}\right) + pSwitch_g^2 \\ &\cdot \left(pDiag_g^{base} \cdot pART_g^{clinic} \cdot (1 - pVS_g^{clinic}) \cdot pVS_g^{cb}\right) \end{aligned}$$

And then calculated the distribution of persons on clinic or community ART in the Home Testing + Community Art scenario as:

$$pCb_g = \frac{\left((pDiag_g^{htc} - pDiag_g^{base}) \cdot pART_g^{cb}\right) + pSwitch_g^1 \cdot \left(pDiag_g^{base} \cdot \left(1 - pART_g^{clinic}\right)\right) + pSwitch_g^2 \cdot \left(pDiag_g^{base} \cdot pART_g^{clinic} \cdot (1 - pVS_g^{clinic})\right)}{\left(pDiag_g^{base} \cdot pART_g^{clinic}\right) + \left((pDiag_g^{htc} - pDiag_g^{base}) \cdot pART_g^{cb}\right) + pSwitch_g^1 \cdot \left(pDiag_g^{base} \cdot \left(1 - pART_g^{clinic}\right)\right)}$$

$$pClinic_g = \frac{\left(pDiag_g^{base} \cdot pART_g^{clinic} \cdot pVS_g^{clinic}\right) + \left(1 - pSwitch_g^2\right) \cdot \left(pDiag_g^{base} \cdot pART_g^{clinic} \cdot (1 - pVS_g^{clinic})\right)}{\left(pDiag_g^{base} \cdot pART_g^{clinic}\right) + \left((pDiag_g^{htc} - pDiag_g^{base}) \cdot pART_g^{cb}\right) + pSwitch_g^1 \cdot \left(pDiag_g^{base} \cdot \left(1 - pART_g^{clinic}\right)\right)}$$

Table T. Percentages of population groups who receive community ART in order to achieve the observed DO ART viral suppression rates at the population-level in Scenario 2.

Population group	Percentage that receives community ART	
	Men	Women
Additional persons with HIV who are diagnosed with home HTC campaigns	100%	100%
Persons with HIV who were diagnosed with baseline HIV testing, but who were not reached by clinic ART	~76.3%*	~12.1%*
Persons with HIV who were diagnosed at baseline and who received clinic ART but who were not virally suppressed	0%	0%
Persons with HIV who were diagnosed, received clinic ART, and virally suppressed	0%	0%

*Percentages are averages over the 2020-2060 timeframe; the exact percent of persons with HIV who know their status fluctuates over time.

(VI.) Outcomes by gender and age

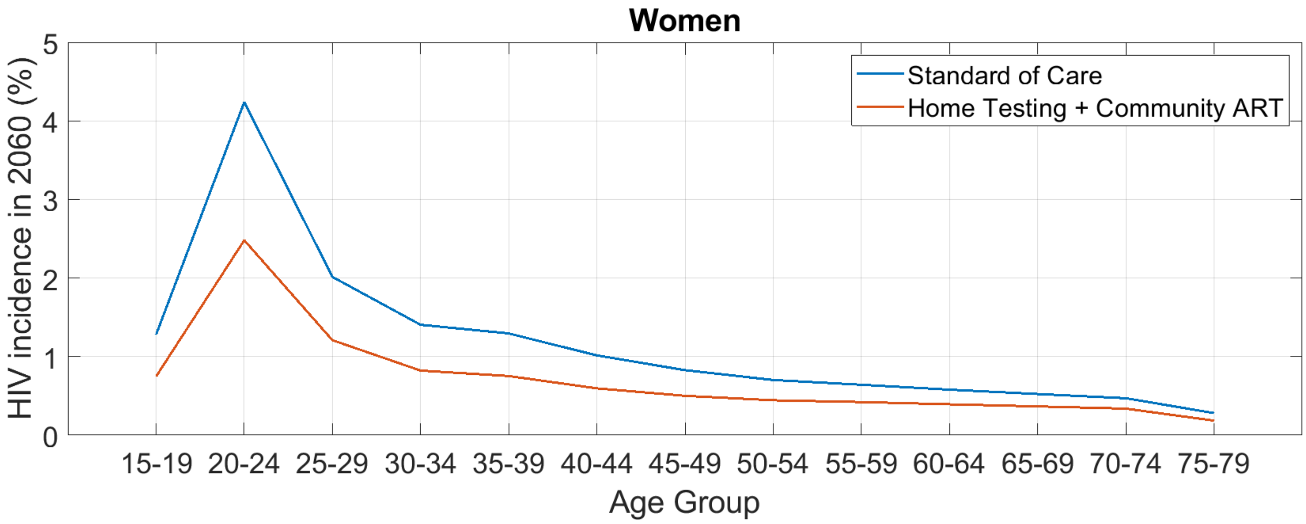

Figure T. Mean projected HIV incidence in 2060 among women by age with the Standard of Care or Home Testing + Community ART.

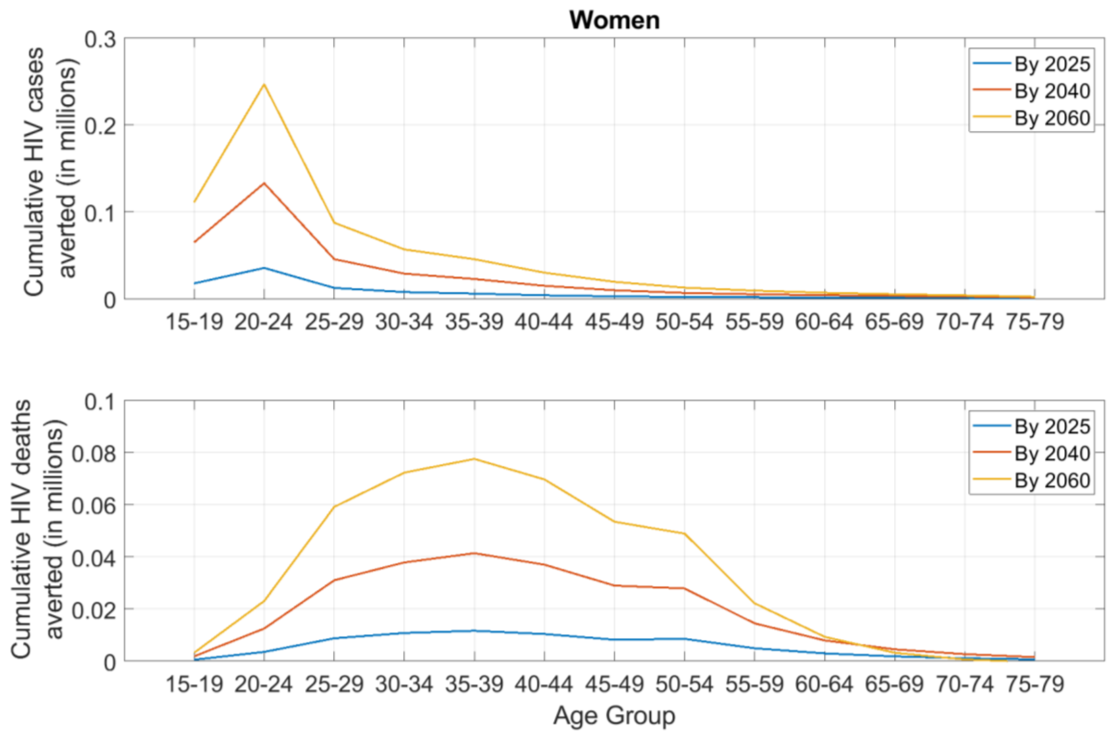

Figure U. Mean projected cumulative HIV cases and deaths averted over time among women by age for Standard of Care compared to Home Testing + Community ART.

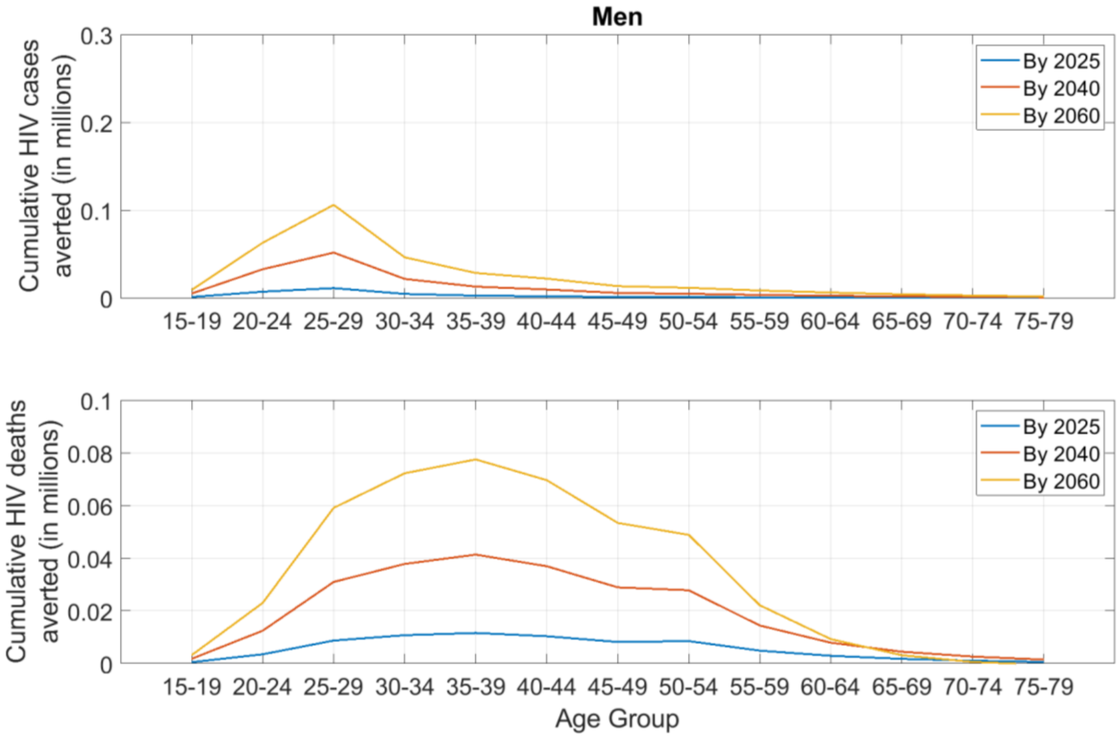

Figure V. Mean projected cumulative HIV cases and deaths averted over time among men by age for Standard of Care compared to Home Testing + Community ART.

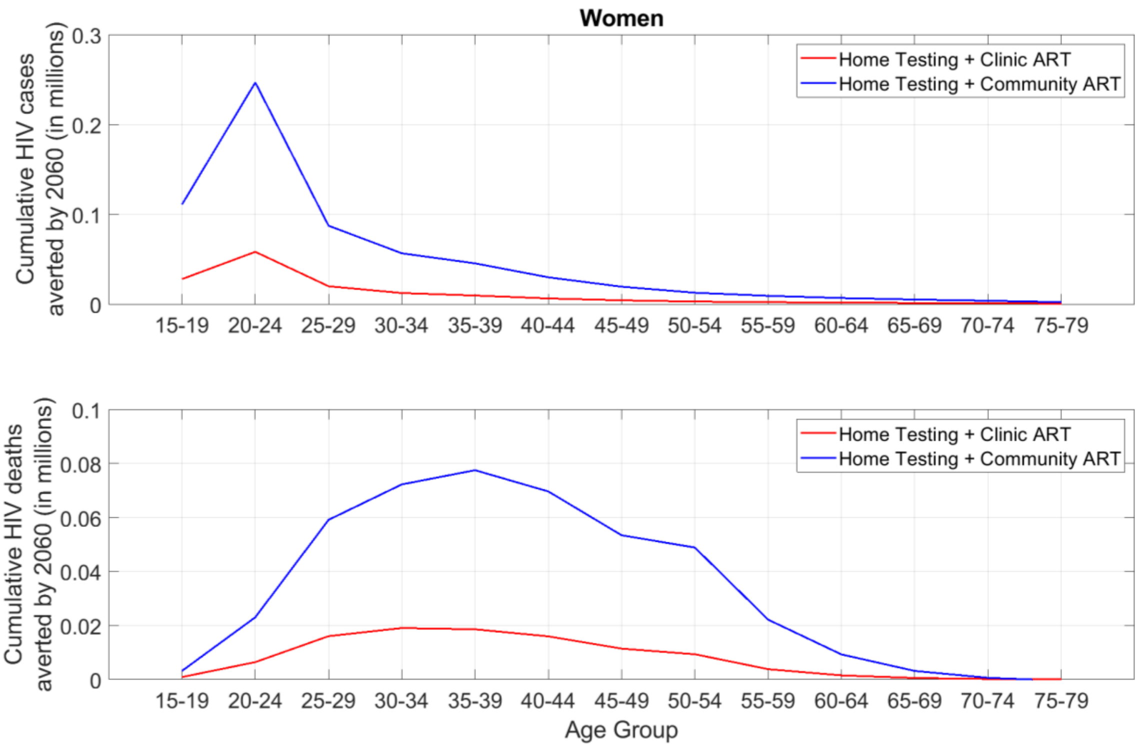

Figure W. Mean projected cumulative HIV cases and deaths averted by 2060 among women by age with Home Testing + Clinic ART (sensitivity analysis) or Home Testing + Community ART.

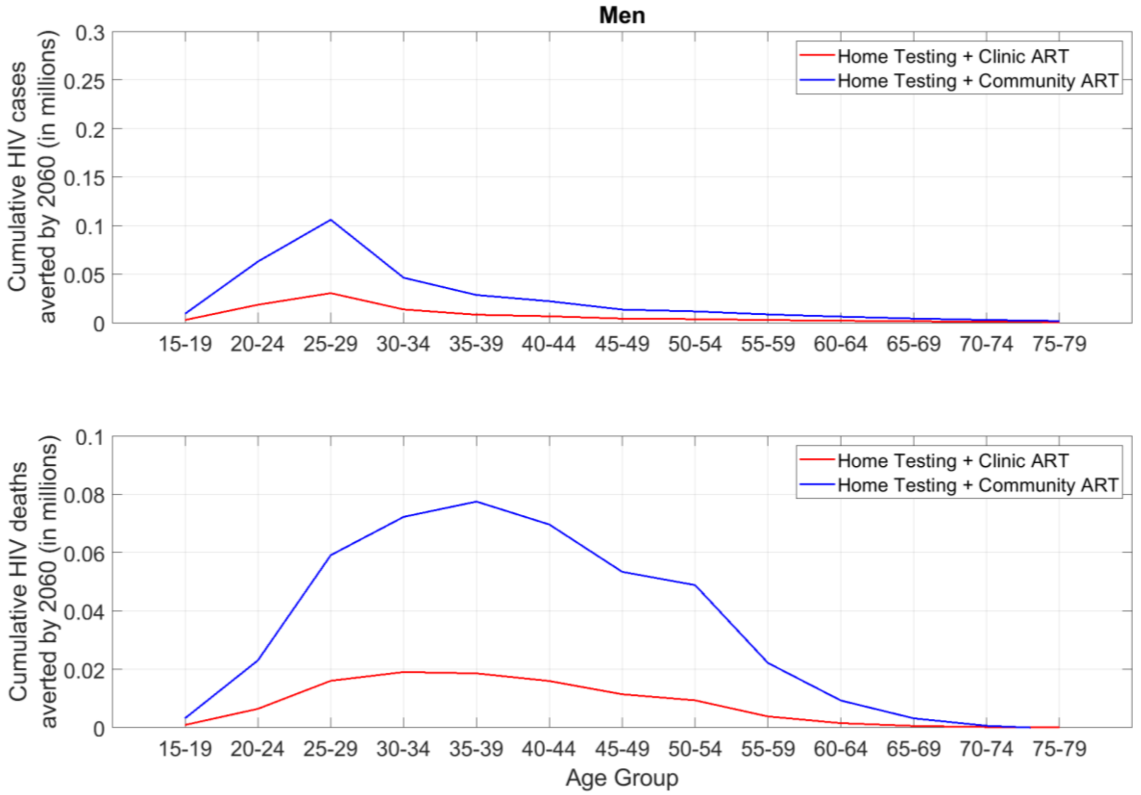

Figure X. Mean projected cumulative HIV cases and deaths averted by 2060 among men by age with Home Testing + Clinic ART (sensitivity analysis) or Home Testing + Community ART.

(VII.) Additional modeling scenarios

(VI.a.) Impact of home HIV testing and counseling campaigns

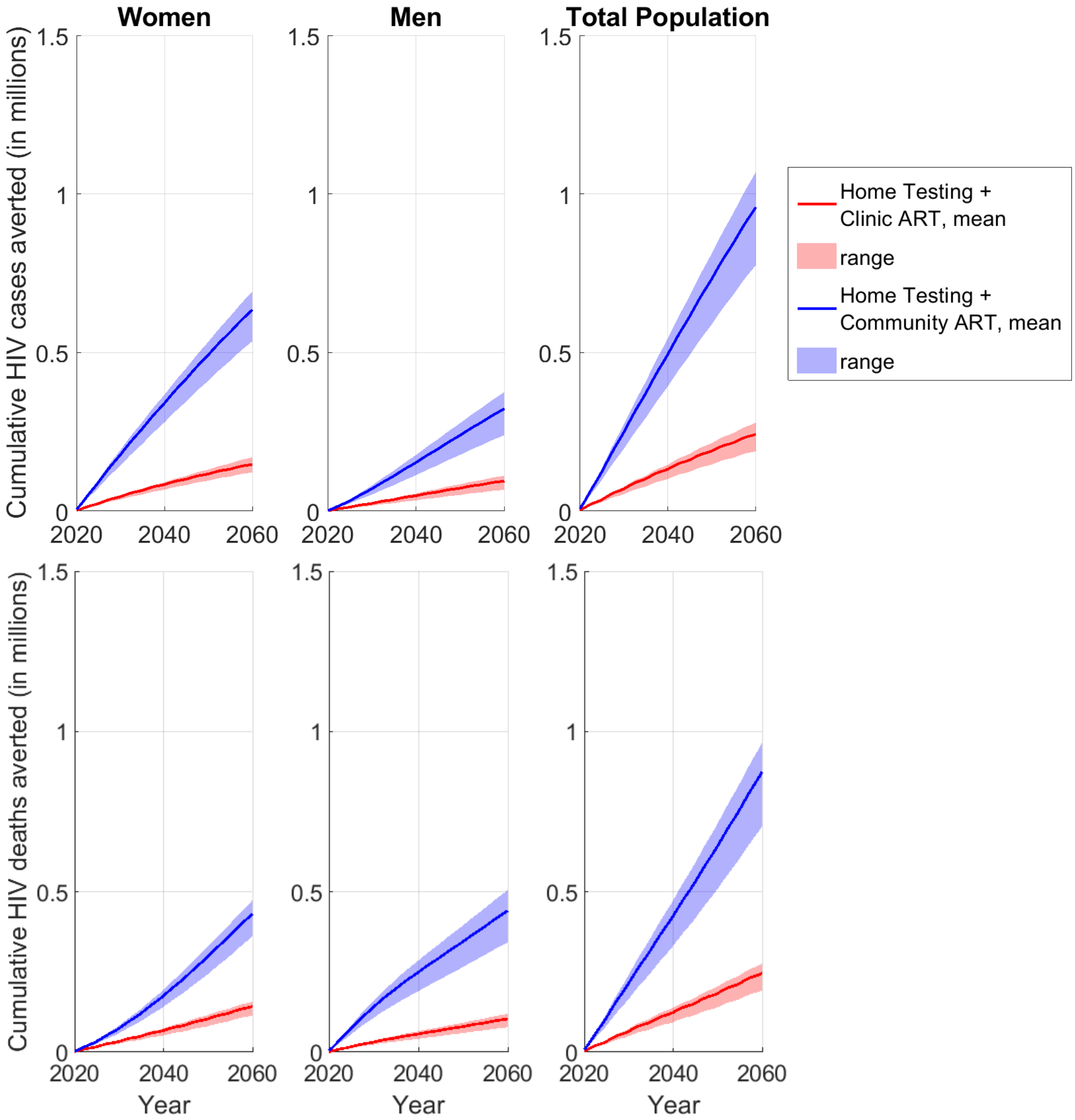

Figure Y: Predicted cumulative HIV cases and deaths averted over time with Home Testing + Clinic ART (sensitivity analysis) or Home Testing + Community ART (main analysis) relative to Clinic ART. Shaded regions represent the range of estimates using the 25 best-fitting model parameter sets.

(VI.b.) Circumcision scale-up sensitivity analysis

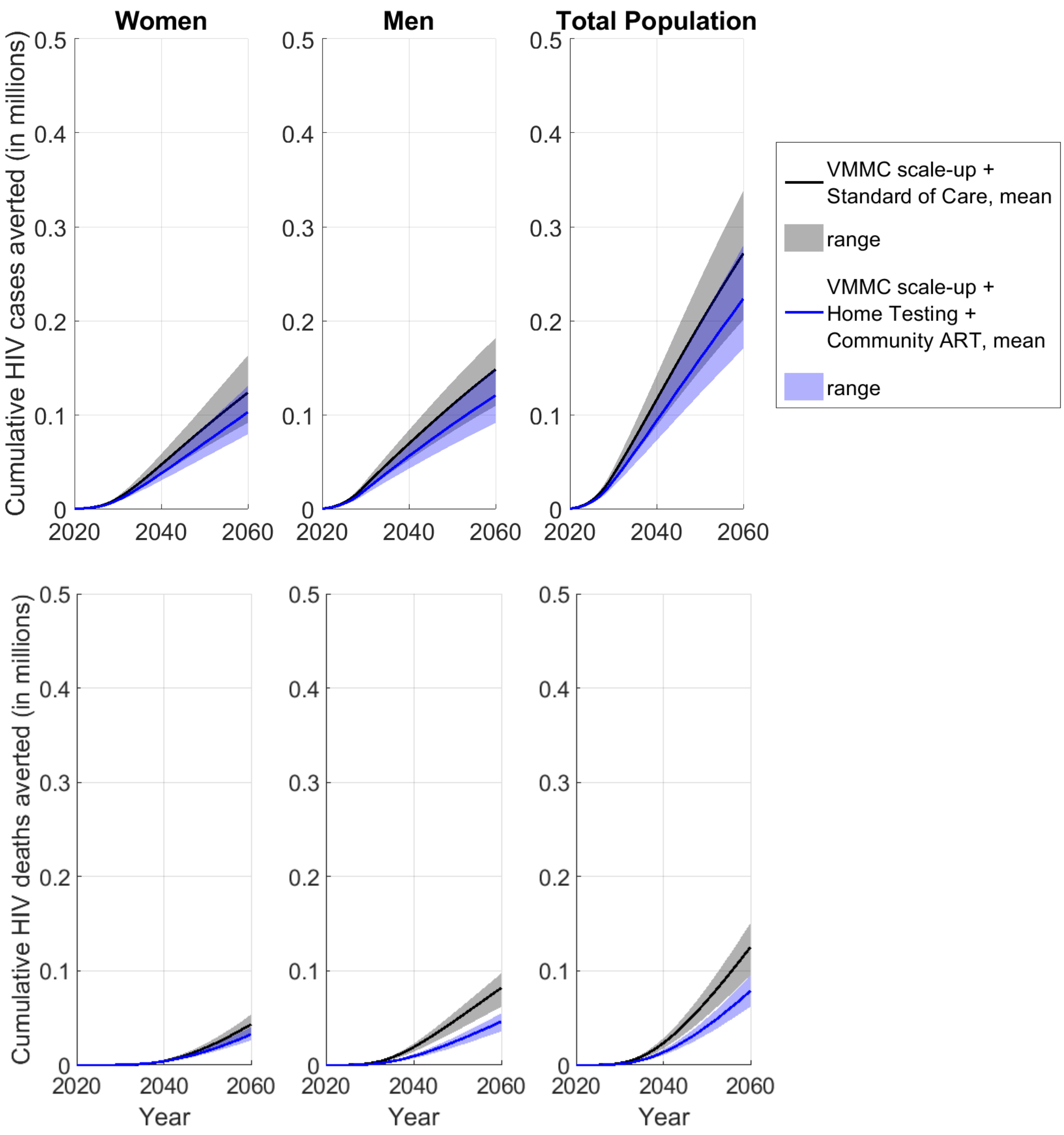

Figure Z: Predicted cumulative HIV cases and deaths averted over time with VMMC scale-up to 72% among all ages and Standard of Care or Home Testing + Community ART (sensitivity analysis) relative to VMMC coverage at the status quo in each scenario, respectively (main analysis). Shaded regions represent the range of estimates using the 25 best-fitting model parameter sets.

Cost-Effectiveness Analysis

(VIII.) Costs

The annual per-client cost of community-based ART was evaluated in the DO ART Study (43). We reviewed the literature to obtain all remaining costs, including the cost of home-based HIV testing and counselling, clinic-based ART, and average hospitalization costs. Where possible, we relied on Meyer-Rath et al.'s systematic review of per-patient costs of HIV services in South Africa (82). Using the currency year reported in the literature, we then inflated the currency to 2020 USD using a deflator series and exchange rates based on the IMF World Economic Outlook (83). In this section we describe the rationale and calculations used for each cost included in the analysis, including the baseline costs as well as the lower and upper bounds used for sensitivity analysis. The final inflation-adjusted values used for the analysis are reported in Table CC.

Table U. Cost for home HIV testing and counselling (HTC) in South Africa. Baseline values used in main analysis are from Meyer et al.'s systematic review of per-patient costs of HIV services (82). The lower bound used in sensitivity analysis reflects the cost of an HIV self-test from OraSure (84). The upper bound used in sensitivity analysis is the average cost per adult tested via the hybrid mobile testing approach in the SEARCH trial, which took place in rural Western Kenya and Uganda (85).

	Per-test cost Baseline [lb, ub]	Currency (Baseline)	Currency (lb)	Currency (ub)
**Home HTC (positive result)**	$5.81 [2.00, 20.50]	2018 USD	2020 USD	2014 USD
**Home HTC (negative result)**	$5.23 [2.00, 20.50]	2018 USD	2020 USD	2014 USD

Table V. Standard of care ART cost for adults in South Africa. The baseline value used in main analysis is from Meyer-Rath et al's National ART Cost Model, which uses a bottom up cost analysis from South Africa's public sector ART programme conducted in the Themba Lethu Clinic (TLC) in Johannesburg to estimate the annual per patient costs for first-line treatment for adults from the provider perspective (38, 82). The lower bound used in sensitivity analysis reflects facility-based care costs in Zambia for patients who are retained at 12 months, using bottom-up micro-costing from the provider perspective (86). The upper bound reflects the costs in the South Africa site of the PopART Trial, evaluated using detailed costing surveys (87).

	Annual per-client cost Baseline [lb, ub]	Currency (baseline)	Currency (lb)	Currency (ub)
**Clinic ART (adults)**	$249.15 [109.55, 315.39]	2018 USD	2018 USD	2017 USD

Table W. Annual community ART cost for adults in KwaZulu-Natal, South Africa. This table shows the annual cost per client for community-based ART and lower and upper bounds, which was evaluated as part of the DO ART Study using activity-based micro-costing from the provider perspective (43). Client volume was projected using time and motion data to estimate costs under varying volume scenarios; the main analysis assumed a client volume of approximately 7-13 clients per work day. Additional calculations and assumptions for baseline, lower, and upper bound costs are described in Tables a-c below.

	**Annual per-client cost Baseline [lb, ub]**	**Currency**
**Community ART (First Year)**	$310.84 [250, 618.68]	2018 USD
**Community ART (Subsequent Years)**	$245.42 [199, 500.88]	2018 USD

Table X. Calculation of baseline annual per-client community ART costs. The baseline cost corresponds to the "efficient-at-scale" cost per client from the South Africa site of the DO ART Study (43). This cost reflects the per client cost of care using the maximum volume of clients that can be seen in an eight-hour workday after removing travel, administrative tasks, and break time and represents a mobile unit servicing one community location per day operating at full capacity.

We used the volume-weighted mean(88) of the "efficient at-scale" costs from two DO ART Study sites located in the Midlands and Northern regions of KwaZulu-Natal (43). Note that for the volume denominator, we used the sample size for the full DO ART study population rather than the population costed. However, given the small difference between the group sizes, the effect of this assumption is marginal.

	**DO ART Study Population (Community ART)**	**Annual per-client cost: First year**	**Annual per-client cost: Subsequent years**	**Currency**
**KZN , Midlands**	283	$312	$246	2018 USD
**KZN, Northern**	116	$308	$244	2018 USD
**KZN, Volume-weighted mean**	283 + 116 = 399	$\frac{(\$312 \cdot 283\ + \$308 \cdot 116)}{399} = \$310.84$	$\frac{(\$246 \cdot 283\ + \$244 \cdot 116)}{399} = \$245.42$	2018 USD

Table Y. Calculation of upper bound annual per-client community-based ART costs. The upper bound reflected the DO ART "steady-state" as-observed scenario cost, i.e. the costs observed in the study including salaries paid in the research study (which are higher than those from the Ministry of Health salary scales), assuming the maximum client volume achieved during the DO ART Study was sustained for 12 months (43). The calculation for the volume-weighted mean is shown below.

	DO ART Study Population (Community ART)	Annual per-client cost: First year	Annual per-client cost: Subsequent years	Currency
**KZN , Midlands**	283	\$637	\$516	2018 USD
**KZN, Northern**	116	\$574	\$464	2018 USD
**KZN, Volume-weighted mean**	283 + 116 = 399	$\frac{(\$637 \cdot 283 \ + \$574 \cdot 116)}{399} = \$618.68$	$\frac{(\$516 \cdot 283 \ + \$464 \cdot 116)}{399} = \$500.88$	2018 USD

Table Z. Calculation of lower bound annual community-based ART costs with six-monthly refills (instead of quarterly). For the lower bound cost of community-based ART, we assumed six-month refills rather than quarterly refills using costs from the DO ART study (for the "efficient-at-scale" scenario assuming maximum client volume). This multi-month scripting scenario assumes 3 visits in the first year (initiation, month 1, and month 6) and 2 visits in the second year (month 12 and month 18). We also assume that the field teams still are able to conduct the same number of visits per day under the respective scenarios. Since the number of visits per person per year has decreased from 5 to 3 (in the first year) and 4 to 2 (in subsequent years), this results in the same field teams being able to treat more total clients per year. Therefore, the fixed costs are spread across a larger number of clients, and the cost per client declines. The 6-month refill strategy also saves in variable costs per client, since fewer encounters per client results in fewer tests and less fuel expended per client.

	Annual per-client cost: First year	Annual per-client cost: Subsequent years	Currency
Annual per-client cost of community ART assuming six-monthly refills	\$250<br>(compared to \$310.84)	\$199<br>(compared to \$245.42)	2018 USD

Table AA. Hospitalization costs, with non-HIV-associated events removed. Average inpatient costs per patient-year in the HIV cohort are obtained from a study that evaluated the average number of hospitalizations and mean cost per stay according to the CD4+ count and ART status in South Africa, with non-HIV related events removed (89). Since our HIV transmission model does not track CD4 count after ART initiation, we assume that all individuals have CD4 >350 post-ART initiation .

	Mean annual cost Baseline [lb, ub]	Currency	Notes
Hospitalization: pre-ART CD4 ≤200 cells/μL	$125.50 [71.50, 222.50]	2009 USD	Meyer-Rath et al. separately report CD4+ ≤100 ($138 [94-292]) and CD4+ 101-200 ($113 [49-153]); we take the mean here
Hospitalization: pre-ART CD4 200-350 cells/μL	$58 [39, 97]	2009 USD	
Hospitalization: pre-ART CD4 >350 cells/μL	$39 [23, 76]	2009 USD	
Hospitalization: post-ART CD4 >350 cells/μL	$45 [23, 80]	2009 USD	

Table BB. Costs of voluntary medical male circumcision (VMMC). In an additional scenario, we consider the results when scaling up HIV prevention through VMMC and the costs associated with this (82).

		Cost per male circumcised Baseline [lb, ub]	Currency
Voluntary medical male circumcision		$95.15 [76.12, 132.90]	2018 USD

Table CC. Intervention costs presented in 2020 USD. Here we present all costs in 2020 USD after adjusting for inflation using the deflator series from the IMF World Economic Outlook (83).

**Intervention**	**Cost (baseline)**	**Cost (lb)**	**Cost (ub)**
Home HTC (positive result), per test	$6.00	$2.00	$22.52
Home HTC (negative result), per test	$5.40	$2.00	$22.52
Clinic-based ART, per client-year	$257.18	$113.08	$333.37
Community ART (Year 1), per client-year	$320.86	$258.06	$638.62
Community ART (subsequent years), per client-year	$253.33	$205.41	$517.02
Hospitalization: pre-ART (CD4 ≤200 cells/µL), per client-year	$150.40	$85.68	$266.64
Hospitalization: pre-ART (CD4 200-350 cells/µL), per client-year	$69.51	$46.74	$116.24
Hospitalization: pre-ART (CD4 >350 cells/µL), per client-year	$46.74	$27.56	$91.08
Hospitalization: post-ART (CD4 >350 cells/µL), per client-year	$53.93	$27.56	$95.87
Voluntary medical male circumcision, per circumcision	$110.41	$88.32	$137.18

(IX.) Total costs for each HIV intervention scenario

Total costs for each HIV intervention scenario are calculated as the sum of HIV testing costs, HIV treatment costs, and hospitalization costs (Section VIII). Prevalent cases incurred treatment and hospitalization costs for the full year, while incident cases accrued these costs for half a year (as an average, to account for variation in the calendar month of diagnosis). Treatment costs are applied to all persons living with HIV on ART. Since the mathematical model only tracks persons on ART with viral suppression, the total number of persons on ART is back-calculated using the ratio $\left(\frac{1}{percent\ who\ achieve\ viral\ suppression\ of\ PLHIV\ who\ know\ their\ status\ and\ are\ on\ ART}\right)$ by gender as a scalar (Table R, Column 2). The total number of persons on ART by ART type and gender is shown in Table DD.

Table DD. Total number of persons on ART, by ART type, gender, and year.

Scenario	ART type	Gender	Number of individuals		
			2020 (mean)	2025 (mean)	2030 (mean)
Clinic ART	Clinic	Men	332,853	303,332	279,634
		Women	760,928	781,604	788,081
	Community	Men	0	0	0
		Women	0	0	0
Home HTC + Community ART	Clinic	Men	271,362	359,998	356,807
		Women	724,912	761,801	736,203
	Community	Men	89,749	119,064	118,009
		Women	61,846	64,993	62,809

(X.) DALY and QALY calculation

Cumulative DALYs = Years of Life Lived with Disability (YLDs) + Years of Life Lost (YLLs)

*We first calculate a Truncated Time Horizon for YLLs which is the lesser of either the full time horizon or the remaining life expectancy for each person at a specific year:

Truncated Time Horizon for YLLs = min(Time Horizon, Year + (Life Expectancy - Age))

*Discount Factor = (1 + Annual Discount Rate)^(Year - 2020))

For each year, age, sex, CD4+ group, and ART status:

1. YLDS = (Population Size – All-cause deaths) * Disability Weight / Discount Factor
2. YLLs = All-cause deaths * sum(1 / Discount Factor) * (1/(1+Discount Rate))^(0:Truncated Time Horizon for YLLs – Year)
3. DALYs = YLLs + YLDs
4. DALYs are summed over time, up to time horizon

Cumulative QALYs

For each year, age, sex, CD4+ group, and ART status:

1. QALYs = (Population Size – All-cause deaths) * QALY Weight / Discount Factor
2. QALYs are summed over time, up to the time horizon

Other notes:

- The baseline Discount Rate is 3% per year
- We use Life Expectancy of 80 given that our population is up to age 79

Table EE. Utility (90) and disability (91) weights.

Health State	QALY Weight	Disability Weight	Notes
**HIV-negative**	1		
**HIV-positive CD4 >350**	0.94	0.078	This is the weight for “HIV/AIDS: receiving ART”
**HIV-positive CD4 200-350**	0.82	0.274	HIV: symptomatic, pre-AIDS
**HIV-positive CD4 <200**	0.70	0.582	AIDS: not receiving ART
**HIV-positive on ART**	0.94	0.078	HIV/AIDS: receiving ART
**Dead**	0		

(XI.) Cost-effectiveness sensitivity analyses

Table FF. Sensitivity analyses for tornado plot.

		Value	Lower	Upper	Source – main	Source – lower	Source – upper
	Costs						
1	Home testing **[vary together]**<br>Positive test<br>Negative test	 5.81<br>5.23	 2<br>2	 20.5<br>20.5	Meyer-Rath, 2019	Shapiro et al self-testing (https://www.orasure.com/)	SEARCH trial (https://www.ncbi.nlm.nih.gov/pmc/articles/PMC5089839/
2	Hospitalization **[vary together]**<br>Annual Hospitalization, CD4 <200<br>Annual Hospitalization, CD4 200-350<br>Annual Hospitalization cost, CD4 350+<br>Annual Hospitalization cost, on ART	 125.5<br>58<br>39<br>45	 71.5<br>39<br>23<br>23	 222.5<br>97<br>76<br>80	Meyer-Rath, 2013	Meyer-Rath, 2013	Meyer-Rath, 2013
3	Community –based ART (1st year)	310.84	249	618.68	DO ART (at-scale)	DO ART 6-month refill (calculated from the DO ART Study	DO ART (as observed)
	Community –based ART (subsequent years)	245.42	199	500.88	DO ART (at-scale)	DO ART 6-month refill (calculated from the DO ART Study)	DO ART (as observed)
4	Standard of care, Clinic ART	249.15	109.55	315.39	Meyer-Rath, 2019	Nichols, AIDS 2020 SDC6 (12 mo retained)	Thomas, Lancet 2021 popART
	Assumptions						
5	Scenario 2: Percentage of people on cb-ART (vs. clinic ART)	See Table 1	0%	100%	DO ART viral suppression rates for clinic v. community	DO ART testing rates for clinic v. community	Illustrative
6	Discount rate	3%	0%	5%	Drummond et al	Drummond et al	Drummond et al

Table GG. Incremental costs, health outcomes, and cost-effectiveness ICER per infection for HTC + Community ART, compared with Standard of Care, with varying time horizons.

	**2030 Time Horizon**	**2045 Time Horizon**	**2060 Time Horizon (Main Analysis)**
**Cost and budget impact** *(undiscounted)*			
Incremental annual programme cost, 2020 to time horizon	50·2 million (UR: 40·2–56·1)	41·8 million (UR: 31·7–48·7)	31·6 million (UR: 21·8–40·5)
Incremental annual programme cost, 2020-2024	44·9 million (UR: 35·8–50·1)	--	--
Additional investment required, 2020-2024	14·3% (UR: 11·4–16·0)	--	--
**Health gains** *(undiscounted)*			
HIV cases averted	248,478 (UR: 196,240–272,776)	614,132 (UR: 491,612–679,125)	957,808 (UR: 775,441–1,068,738)
HIV deaths averted	214,589 (UR: 165,767–240,349)	531,871 (UR: 417,995–590,715)	874,015 (UR: 703,693–965,636)
DALYs averted	1·3 million (UR: 1–1·5)	6·1 million (UR: 4·8–6·8)	13·0 million (UR: 10·5–14·5)
**Cost-effectiveness** *(discounted 3% for both costs and health gains)*			
Cost per case averted	$2237 (UR: 2029–2340)	$1857 (UR: 1540–2095)	$1570 (UR: 1206–1950)
Cost per death averted	$2592 (UR: 2517–2689)	$2145 (UR: 1954–2261)	$1748 (UR: 1451–2013)
Cost per DALY averted	$400 (UR: 392–413)	$171 (UR: 157–180)	$102 (UR: 85–117)

Notes: *Costs are presented in 2020 USD. Values are the mean of 25 best-fitting parameter sets. Uncertainty ranges (URs) represent the minimum and maximum for the 25 best-fitting parameter sets from the model projections.*

Figure AA. Tornado diagram for time horizon of 2030.

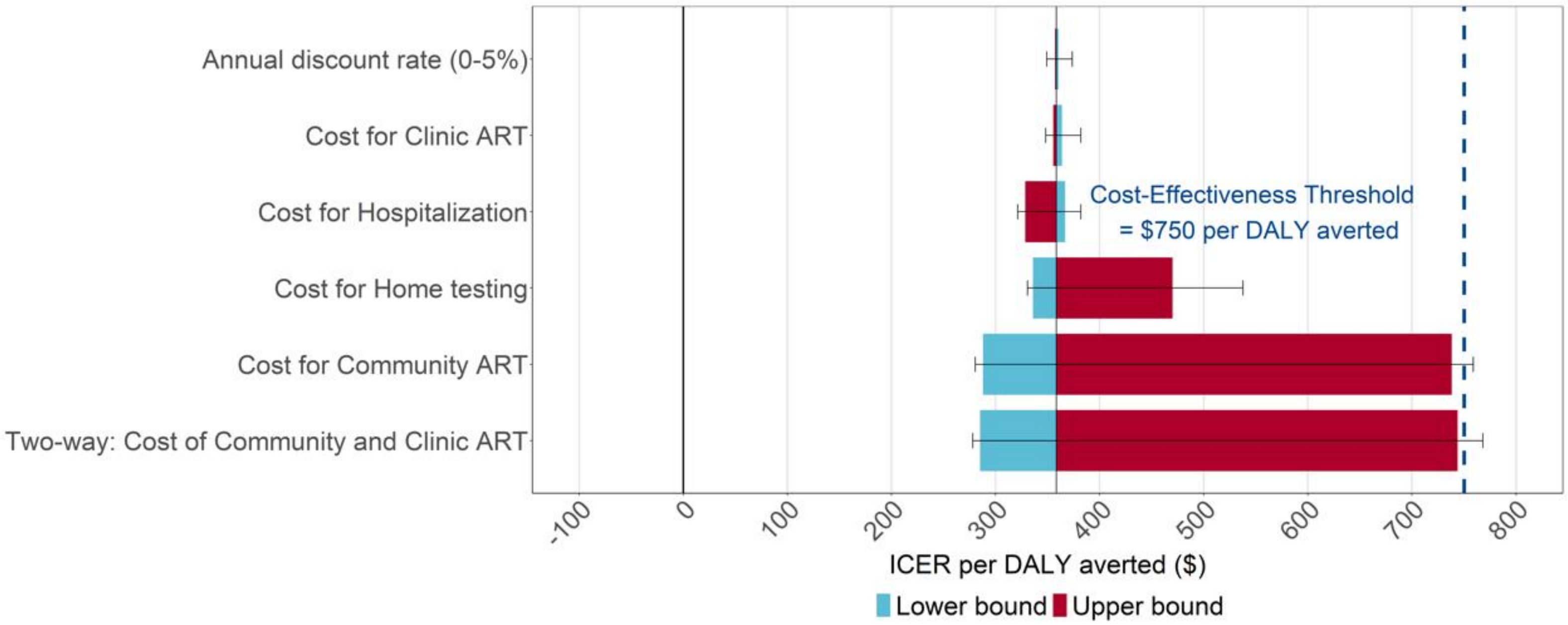

Figure BB. Tornado diagram for time horizon of 2045.

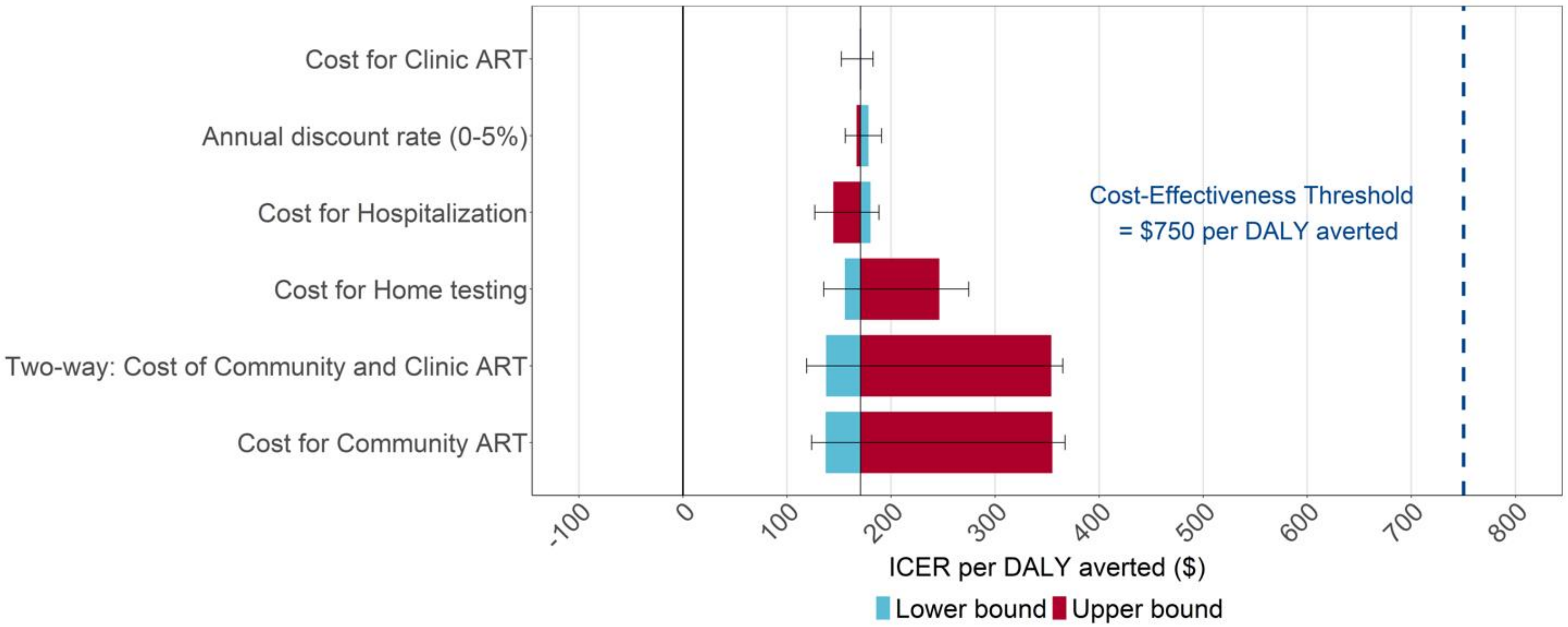

References

1. National Department of Health (NDoH) SSASS, South African Medical Research, Council (SAMRC) I. South Africa Demographic and Health Survey 2016. Pretoria, South Africa, and Rockville, Maryland, USA; 2019.
2. Simbayi LC ZK, Zungu N, Moyo S, Marinda E, Jooste S, Mabaso M, Ramlagan S, North A, van Zyl J, Mohlabane N, Dietrich C, Naidoo I, SABSSM V Team. The Fifth South African National HIV Prevalence, Incidence, Behaviour and Communications Survey, 2017. Cape Town; 2019.
3. Tan N, Sharma M, Winer R, Galloway D, Rees H, Barnabas RV. Model-estimated effectiveness of single dose 9-valent HPV vaccination for HIV-positive and HIV-negative females in South Africa. Vaccine. 2018;36(32 Pt A):4830-6.
4. Department of Economic and Social Affairs PD. World Population Prospects 2019. In: Nations U, editor. Rev. 1 ed. Online Edition2019.
5. Africa SS. Mid-year population estimates 2019. Statistical Release P0302. Pretoria, South Africa2019.
6. Moultrie TA, Hosegood V, McGrath N, Hill C, Herbst K, Newell ML. Refining the criteria for stalled fertility declines: an application to rural KwaZulu-Natal, South Africa, 1990-2005. Stud Fam Plann. 2008;39(1):39-48.
7. Moultrie TA, Timaeus IM. The South African fertility decline: Evidence from two censuses and a Demographic and Health Survey. Popul Stud (Camb). 2003;57(3):265-83.
8. Anderson RM, May RM, Ng TW, Rowley JT. Age-dependent choice of sexual partners and the transmission dynamics of HIV in Sub-Saharan Africa. Philos Trans R Soc Lond B Biol Sci. 1992;336(1277):135-55.
9. Ross A, Van der Paal L, Lubega R, Mayanja BN, Shafer LA, Whitworth J. HIV-1 disease progression and fertility: the incidence of recognized pregnancy and pregnancy outcome in Uganda. AIDS. 2004;18(5):799-804.
10. Bobat R, Coovadia H, Coutsoudis A, Moodley D. Determinants of mother-to-child transmission of human immunodeficiency virus type 1 infection in a cohort from Durban, South Africa. Pediatr Infect Dis J. 1996;15(7):604-10.
11. Horwood C, Vermaak K, Butler L, Haskins L, Phakathi S, Rollins N. Elimination of paediatric HIV in KwaZulu-Natal, South Africa: large-scale assessment of interventions for the prevention of mother-to-child transmission. Bull World Health Organ. 2012;90(3):168-75.
12. Rollins N, Little K, Mzolo S, Horwood C, Newell ML. Surveillance of mother-to-child transmission prevention programmes at immunization clinics: the case for universal screening. AIDS. 2007;21(10):1341-7.
13. Network GBoDC. Global Burden of Disease Study 2017 (GBD 2017) Results. In: (IHME) IfHMaE, editor. Seattle, United States2018.
14. Institute AHR. Africa Centre cohort data from KwaZulu-Natal, SA Surveillance data repository. Durban, South Africa.
15. Rao DW, Wheatley MM, Goodreau SM, Enns EA. Partnership dynamics in mathematical models and implications for representation of sexually transmitted infections: a review. Annals of epidemiology. 2021;59:72-80.
16. Garnett GP, Gregson S. Monitoring the course of the HIV-1 epidemic: The influence of patterns of fertility on HIV-1 prevalence estimates. Mathematical Population Studies. 2000;8:251-77.
17. Ott MQ, Barnighausen T, Tanser F, Lurie MN, Newell ML. Age-gaps in sexual partnerships: seeing beyond 'sugar daddies'. AIDS. 2011;25(6):861-3.
18. de Oliveira T, Kharsany AB, Graf T, Cawood C, Khanyile D, Grobler A, et al. Transmission networks and risk of HIV infection in KwaZulu-Natal, South Africa: a community-wide phylogenetic study. The lancet HIV. 2017;4(1):e41-e50.

19. Hubert JB, Burgard M, Dussaix E, Tamalet C, Deveau C, Le Chenadec J, et al. Natural history of serum HIV-1 RNA levels in 330 patients with a known date of infection. The SEROCO Study Group. AIDS. 2000;14(2):123-31.
20. Lodi S, Phillips A, Touloumi G, Geskus R, Meyer L, Thiebaut R, et al. Time from human immunodeficiency virus seroconversion to reaching CD4+ cell count thresholds <200, <350, and <500 Cells/mm(3): assessment of need following changes in treatment guidelines. Clinical infectious diseases : an official publication of the Infectious Diseases Society of America. 2011;53(8):817-25.
21. Lyles RH, Munoz A, Yamashita TE, Bazmi H, Detels R, Rinaldo CR, et al. Natural history of human immunodeficiency virus type 1 viremia after seroconversion and proximal to AIDS in a large cohort of homosexual men. Multicenter AIDS Cohort Study. The Journal of infectious diseases. 2000;181(3):872-80.
22. Pantazis N, Morrison C, Amornkul PN, Lewden C, Salata RA, Minga A, et al. Differences in HIV natural history among African and non-African seroconverters in Europe and seroconverters in sub-Saharan Africa. PLoS One. 2012;7(3):e32369.
23. Badri M, Lawn SD, Wood R. Short-term risk of AIDS or death in people infected with HIV-1 before antiretroviral therapy in South Africa: a longitudinal study. Lancet (London, England). 2006;368(9543):1254-9.
24. Lewden C, Gabillard D, Minga A, Ekouevi DK, Avit D, Konate I, et al. CD4-specific mortality rates among HIV-infected adults with high CD4 counts and no antiretroviral treatment in West Africa. Journal of acquired immune deficiency syndromes (1999). 2012;59(2):213-9.
25. Maduna PH, Dolan M, Kondlo L, Mabuza H, Dlamini JN, Polis M, et al. Morbidity and mortality according to latest CD4+ cell count among HIV positive individuals in South Africa who enrolled in project Phidisa. PLoS One. 2015;10(4):e0121843.
26. Adler WH, Baskar PV, Chrest FJ, Dorsey-Cooper B, Winchurch RA, Nagel JE. HIV infection and aging: mechanisms to explain the accelerated rate of progression in the older patient. Mech Ageing Dev. 1997;96(1-3):137-55.
27. Newell ML, Coovadia H, Cortina-Borja M, Rollins N, Gaillard P, Dabis F, et al. Mortality of infected and uninfected infants born to HIV-infected mothers in Africa: a pooled analysis. Lancet (London, England). 2004;364(9441):1236-43.
28. Quinn TC, Wawer MJ, Sewankambo N, Serwadda D, Li C, Wabwire-Mangen F, et al. Viral load and heterosexual transmission of human immunodeficiency virus type 1. Rakai Project Study Group. The New England journal of medicine. 2000;342(13):921-9.
29. Lingappa JR, Hughes JP, Wang RS, Baeten JM, Celum C, Gray GE, et al. Estimating the impact of plasma HIV-1 RNA reductions on heterosexual HIV-1 transmission risk. PLoS One. 2010;5(9):e12598.
30. Hollingsworth TD, Anderson RM, Fraser C. HIV-1 transmission, by stage of infection. The Journal of infectious diseases. 2008;198(5):687-93.
31. Boily MC, Baggaley RF, Wang L, Masse B, White RG, Hayes RJ, et al. Heterosexual risk of HIV-1 infection per sexual act: systematic review and meta-analysis of observational studies. The Lancet Infectious diseases. 2009;9(2):118-29.
32. Rodger AJ, Cambiano V, Bruun T, Vernazza P, Collins S, van Lunzen J, et al. Sexual Activity Without Condoms and Risk of HIV Transmission in Serodifferent Couples When the HIV-Positive Partner Is Using Suppressive Antiretroviral Therapy. Jama. 2016;316(2):171-81.
33. Eisinger RW, Dieffenbach CW, Fauci AS. HIV Viral Load and Transmissibility of HIV Infection: Undetectable Equals Untransmittable. Jama. 2019;321(5):451-2.
34. Brinkhof MW, Boulle A, Weigel R, Messou E, Mathers C, Orrell C, et al. Mortality of HIV-infected patients starting antiretroviral therapy in sub-Saharan Africa: comparison with HIV-unrelated mortality. PLoS medicine. 2009;6(4):e1000066.

35. Cornell M, Johnson LF, Wood R, Tanser F, Fox MP, Prozesky H, et al. Twelve-year mortality in adults initiating antiretroviral therapy in South Africa. Journal of the International AIDS Society. 2017;20(1):21902.
36. de Coninck Z, Hussain-Alkhateeb L, Bratt G, Ekstrom AM, Gisslen M, Petzold M, et al. Non-AIDS Mortality Is Higher Among Successfully Treated People Living with HIV Compared with Matched HIV-Negative Control Persons: A 15-Year Follow-Up Cohort Study in Sweden. AIDS patient care and STDs. 2018;32(8):297-305.
37. Lilian RR, Rees K, Mabitsi M, McIntyre JA, Struthers HE, Peters RPH. Baseline CD4 and mortality trends in the South African human immunodeficiency virus programme: Analysis of routine data. South Afr J HIV Med. 2019;20(1):963.
38. Meyer-Rath G, Johnson LF, Pillay Y, Blecher M, Brennan AT, Long L, et al. Changing the South African national antiretroviral therapy guidelines: The role of cost modelling. PLoS One. 2017;12(10):e0186557.
39. UNAIDS. South Africa2020 [Available from: https://www.unaids.org/en/regionscountries/countries/southafrica.
40. Kharsany ABM, Cawood C, Lewis L, Yende-Zuma N, Khanyile D, Puren A, et al. Trends in HIV Prevention, Treatment, and Incidence in a Hyperendemic Area of KwaZulu-Natal, South Africa. JAMA Netw Open. 2019;2(11):e1914378.
41. Takuva S, Brown AE, Pillay Y, Delpech V, Puren AJ. The continuum of HIV care in South Africa: implications for achieving the second and third UNAIDS 90-90-90 targets. AIDS. 2017;31(4):545-52.
42. Zaidi J, Grapsa E, Tanser F, Newell ML, Barnighausen T. Dramatic increase in HIV prevalence after scale-up of antiretroviral treatment. AIDS. 2013;27(14):2301-5.
43. Barnabas RV, Szpiro AA, van Rooyen H, Asiimwe S, Pillay D, Ware NC, et al. Community-based antiretroviral therapy versus standard clinic-based services for HIV in South Africa and Uganda (DO ART): a randomised trial. The Lancet Global health. 2020;8(10):e1305-e15.
44. Weller S, Davis K. Condom effectiveness in reducing heterosexual HIV transmission. Cochrane Database Syst Rev. 2002(1):CD003255.
45. Gray RH, Kigozi G, Serwadda D, Makumbi F, Watya S, Nalugoda F, et al. Male circumcision for HIV prevention in men in Rakai, Uganda: a randomised trial. Lancet (London, England). 2007;369(9562):657-66.
46. Weiss HA, Quigley MA, Hayes RJ. Male circumcision and risk of HIV infection in sub-Saharan Africa: a systematic review and meta-analysis. AIDS. 2000;14(15):2361-70.
47. Tobian AA, Kong X, Wawer MJ, Kigozi G, Gravitt PE, Serwadda D, et al. Circumcision of HIV-infected men and transmission of human papillomavirus to female partners: analyses of data from a randomised trial in Rakai, Uganda. The Lancet Infectious diseases. 2011;11(8):604-12.
48. Weiss HA, Hankins CA, Dickson K. Male circumcision and risk of HIV infection in women: a systematic review and meta-analysis. The Lancet Infectious diseases. 2009;9(11):669-77.
49. Lei JH, Liu LR, Wei Q, Yan SB, Yang L, Song TR, et al. Circumcision Status and Risk of HIV Acquisition during Heterosexual Intercourse for Both Males and Females: A Meta-Analysis. PLoS One. 2015;10(5):e0125436.
50. Connolly C, Simbayi LC, Shanmugam R, Nqeketo A. Male circumcision and its relationship to HIV infection in South Africa: results of a national survey in 2002. S Afr Med J. 2008;98(10):789-94.
51. Doyle D. Ritual male circumcision: a brief history. J R Coll Physicians Edinb. 2005;35(3):279-85.
52. HIV/AIDS WHOaJUNPo. Male circumcision: global trends and determinants of prevalence, safety and acceptability. Switzerland2007.
53. Shisana O RT, Simbayi LC, Zuma K, Jooste S, Zungu N, Labadarios D,, Onoya D ea. South African National HIV Prevalence, Incidence and Behaviour Survey, 2012. Cape Town; 2014.

54. Shisana O SL, Rehle T, Zungu NP, Zuma K, Ngogo N, Jooste S, PillayVan Wyk V, Parker W, Pezi S, Davids A, Nwanyanwu O, Dinh TH, SABSSM III Implementation, Team. South African National HIV Prevalence, Incidence, Behaviour and Communication Survey, 2008: The health of our children. Cape Town; 2010.
55. UNAIDS. Progress towards the Start Free, Stay Free, AIDS Free targets: 2020 report. 2020.
56. Sharma M, Ying R, Tarr G, Barnabas R. Systematic review and meta-analysis of community and facility-based HIV testing to address linkage to care gaps in sub-Saharan Africa. Nature. 2015;528(7580):S77-85.
57. Vandormael A, de Oliveira T, Tanser F, Barnighausen T, Herbeck JT. High percentage of undiagnosed HIV cases within a hyperendemic South African community: a population-based study. J Epidemiol Community Health. 2018;72(2):168-72.
58. Barnabas RV, van Rooyen H, Tumwesigye E, Brantley J, Baeten JM, van Heerden A, et al. Uptake of antiretroviral therapy and male circumcision after community-based HIV testing and strategies for linkage to care versus standard clinic referral: a multisite, open-label, randomised controlled trial in South Africa and Uganda. The lancet HIV. 2016;3(5):e212-20.
59. Brisson MVdV, Nicolas; Drolet, Melanie; Laprise, Jean-Francois; Boily, Marie-Claude. TECHNICAL APPENDIX HPV-ADVISE2012-2016.
60. Filippi S, Barnes CP, Cornebise J, Stumpf MPH. On optimality of kernels for approximate Bayesian computation using sequential Monte Carlo. Stat Appl Genet Mol. 2013;12(1).
61. Lenormand M, Jabot F, Deffuant G. Adaptive approximate Bayesian computation for complex models. Computation Stat. 2013;28(6):2777-96.
62. Toni T, Welch D, Strelkowa N, Ipsen A, Stumpf MPH. Approximate Bayesian computation scheme for parameter inference and model selection in dynamical systems. J R Soc Interface. 2009;6(31):187-202.
63. McDonald AC, Tergas AI, Kuhn L, Denny L, Wright TC, Jr. Distribution of Human Papillomavirus Genotypes among HIV-Positive and HIV-Negative Women in Cape Town, South Africa. Front Oncol. 2014;4:48.
64. Mbulawa ZZ, Coetzee D, Williamson AL. Human papillomavirus prevalence in South African women and men according to age and human immunodeficiency virus status. BMC infectious diseases. 2015;15:459.
65. Kuhn L, Saidu R, Boa R, Tergas A, Moodley J, Persing D, et al. Clinical evaluation of modifications to a human papillomavirus assay to optimise its utility for cervical cancer screening in low-resource settings: a diagnostic accuracy study. The Lancet Global health. 2020;8(2):e296-e304.
66. Bray F, Ferlay J, Soerjomataram I, Siegel RL, Torre LA, Jemal A. Global cancer statistics 2018: GLOBOCAN estimates of incidence and mortality worldwide for 36 cancers in 185 countries. CA Cancer J Clin. 2018;68(6):394-424.
67. Bruni L AG, Serrano B, Mena M, Gómez D, Muñoz J, Bosch FX, de Sanjosé S. Human Papillomavirus and Related Diseases in South Africa: Summary Report 17 June 2019.
68. Van Aardt MC, Dreyer G, Richter KL, Becker P. Human papillomavirus-type distribution in South African women without cytological abnormalities: a peri-urban study. Southern African Journal of Gynaecological Oncology. 2013;5(sup1):S21-S7.
69. van Aardt MC, Dreyer G, Pienaar HF, Karlsen F, Hovland S, Richter KL, et al. Unique human papillomavirus-type distribution in South African women with invasive cervical cancer and the effect of human immunodeficiency virus infection. International journal of gynecological cancer : official journal of the International Gynecological Cancer Society. 2015;25(5):919-25.
70. Van Aardt MC, Dreyer G, Snyman LC, Richter KL, Becker P, Mojaki SM. Oncogenic and incidental HPV types associated with histologically confirmed cervical intraepithelial neoplasia in HIV-positive and HIV-negative South African women. S Afr Med J. 2016;106(6).
71. Clifford GM, Rana RK, Franceschi S, Smith JS, Gough G, Pimenta JM. Human papillomavirus genotype distribution in low-grade cervical lesions: comparison by geographic region and with cervical cancer. Cancer epidemiology, biomarkers & prevention : a publication of the American Association for Cancer Research, cosponsored by the American Society of Preventive Oncology. 2005;14(5):1157-64.

72. Dartell M, Rasch V, Kahesa C, Mwaiselage J, Ngoma T, Junge J, et al. Human papillomavirus prevalence and type distribution in 3603 HIV-positive and HIV-negative women in the general population of Tanzania: the PROTECT study. Sexually transmitted diseases. 2012;39(3):201-8.
73. de Sanjose S, Quint WG, Alemany L, Geraets DT, Klaustermeier JE, Lloveras B, et al. Human papillomavirus genotype attribution in invasive cervical cancer: a retrospective cross-sectional worldwide study. Lancet Oncol. 2010;11(11):1048-56.
74. Denny L, Adewole I, Anorlu R, Dreyer G, Moodley M, Smith T, et al. Human papillomavirus prevalence and type distribution in invasive cervical cancer in sub-Saharan Africa. International journal of cancer. 2014;134(6):1389-98.
75. Beauclair R, Helleringer S, Hens N, Delva W. Age differences between sexual partners, behavioural and demographic correlates, and HIV infection on Likoma Island, Malawi. Sci Rep. 2016;6:36121.
76. Mabaso M, Mlangeni L, Makola L, Oladimeji O, Naidoo I, Naidoo Y, et al. Factors associated with age-disparate sexual partnerships among males and females in South Africa: a multinomial analysis of the 2012 national population-based household survey data. Emerg Themes Epidemiol. 2021;18(1):3.
77. Africa SS. Census 2001: Post-enumeration survey: Results and methodology. Pretoria, South Africa2004.
78. Africa SS. Census 2011: Post-enumeration Survey: Results and methodology. Pretoria, South Africa2012.
79. Africa SS. Census 2001: Primary tables KwaZulu-Natal: 1996 and 2001 compared. Pretoria, South Africa2004.
80. Africa SS. Community Survey 2016. Statistical release P0301. Pretoria, South Africa2016.
81. Vandormael A, Akullian A, Siedner M, de Oliveira T, Barnighausen T, Tanser F. Declines in HIV incidence among men and women in a South African population-based cohort. Nat Commun. 2019;10(1):5482.
82. Meyer-Rath G, van Rensburg C, Chiu C, Leuner R, Jamieson L, Cohen S. The per-patient costs of HIV services in South Africa: Systematic review and application in the South African HIV Investment Case. PLoS One. 2019;14(2):e0210497.
83. International_Monetary_Fund. World Economic Outlook 2015 [Available from: http://www.imf.org/external/pubs/ft/weo/2015/01/weodata/index.aspx
84. Shapiro AE, van Heerden A, Krows M, Sausi K, Sithole N, Schaafsma TT, et al. An implementation study of oral and blood-based HIV self-testing and linkage to care among men in rural and peri-urban KwaZulu-Natal, South Africa. Journal of the International AIDS Society. 2020;23 Suppl 2:e25514.
85. Chang W, Chamie G, Mwai D, Clark TD, Thirumurthy H, Charlebois ED, et al. Implementation and Operational Research: Cost and Efficiency of a Hybrid Mobile Multidisease Testing Approach With High HIV Testing Coverage in East Africa. Journal of acquired immune deficiency syndromes (1999). 2016;73(3):e39-e45.
86. Nichols BE, Cele R, Jamieson L, Long LC, Siwale Z, Banda P, et al. Community-based delivery of HIV treatment in Zambia: costs and outcomes. AIDS. 2021;35(2):299-306.
87. Thomas R, Probert WJM, Sauter R, Mwenge L, Singh S, Kanema S, et al. Cost and cost-effectiveness of a universal HIV testing and treatment intervention in Zambia and South Africa: evidence and projections from the HPTN 071 (PopART) trial. The Lancet Global health. 2021;9(5):e668-e80.
88. Clarke-Deelder E, Vassall A, Menzies NA. Estimators Used in Multisite Healthcare Costing Studies in Low- and Middle-Income Countries: A Systematic Review and Simulation Study. Value Health. 2019;22(10):1146-53.
89. Meyer-Rath G, Brennan AT, Fox MP, Modisenyane T, Tshabangu N, Mohapi L, et al. Rates and cost of hospitalization before and after initiation of antiretroviral therapy in urban and rural settings in South Africa. Journal of acquired immune deficiency syndromes (1999). 2013;62(3):322-8.
90. Tengs TO, Lin TH. A meta-analysis of utility estimates for HIV/AIDS. Med Decis Making. 2002;22(6):475-81.
91. Network GBoDC. Global Burden of Disease Study 2019 (GBD 2019) Disability Weights. Seattle, United States of America: Institute for Health Metrics and Evaluation (IHME); 2020.
